# County-level longitudinal clustering of COVID-19 mortality to incidence ratio in the United States

**DOI:** 10.1038/s41598-021-82384-0

**Published:** 2021-02-04

**Authors:** Nasim Vahabi, Masoud Salehi, Julio D. Duarte, Abolfazl Mollalo, George Michailidis

**Affiliations:** 1 Informatics Institute, University of Florida, Gainesville, FL USA; 2 Department of Biostatistics, College of Public Health, Iran University of Medical Sciences, Tehran, Iran; 3 Center for Pharmacogenomics, Department of Pharmacotherapy and Translational Research, College of Pharmacy, University of Florida, Gainesville, FL USA; 4 Department of Public Health and Prevention Sciences, School of Health Sciences, Baldwin Wallace University, Berea, OH USA

**Keywords:** Infectious diseases, Risk factors, Experimental models of disease

## Abstract

As of November 12, 2020, the mortality to incidence ratio (MIR) of COVID-19 was 5.8% in the US. A longitudinal model-based clustering system on the disease trajectories over time was used to identify “vulnerable” clusters of counties that would benefit from allocating additional resources by federal, state and county policymakers. County-level COVID-19 cases and deaths, together with a set of potential risk factors were collected for 3050 U.S. counties during the 1st wave of COVID-19 (Mar25–Jun3, 2020), followed by similar data for 1344 counties (in the “sunbelt” region of the country) during the 2nd wave (Jun4–Sep2, 2020), and finally for 1055 counties located broadly in the great plains region of the country during the 3rd wave (Sep3–Nov12, 2020). We used growth mixture models to identify clusters of counties exhibiting similar COVID-19 MIR growth trajectories and risk-factors over time. The analysis identifies “more vulnerable” clusters during the 1st, 2nd and 3rd waves of COVID-19. Further, tuberculosis (OR 1.3–2.1–3.2), drug use disorder (OR 1.1), hepatitis (OR 13.1), HIV/AIDS (OR 2.3), cardiomyopathy and myocarditis (OR 1.3), diabetes (OR 1.2), mesothelioma (OR 9.3) were significantly associated with increased odds of being in a more vulnerable cluster. Heart complications and cancer were the main risk factors increasing the COVID-19 MIR (range 0.08–0.52% MIR↑). We identified “more vulnerable” county-clusters exhibiting the highest COVID-19 MIR trajectories, indicating that enhancing the capacity and access to healthcare resources would be key to successfully manage COVID-19 in these clusters. These findings provide insights for public health policymakers on the groups of people and locations they need to pay particular attention while managing the COVID-19 epidemic.

## Introduction

As of Nov 2020, the total number of confirmed COVID-19 (caused by the SARS-CoV-2 virus) cases and deaths worldwide were 50,676,072 and 1,261,075, respectively. COVID-19 was first discovered in Wuhan, China, on December 31, 2019. The outbreak of the disease was declared on Jan 30, 2020, and eventually was declared as a pandemic by the World Health Organization (WHO) on Mar 11, 2020^1^. Shortly after, few countries, most notably Iran and Italy, experienced a significant increase in the number of confirmed cases and deaths^1^.

As of Nov 2020, the total number of confirmed COVID-19 cases and deaths in the United States were 9,913,553 and 237,037, respectively. The mortality rate (MR) was 71.7 per 100,000 population, and the mortality to incidence ratio was 2.4%, i.e., 2.4% of the COVID-19 confirmed cases experienced death as the outcome (U.S. population on Nov 2020 was 330.57 million) (https://usafacts.org). Within the United States, according to the Center for Disease Control and Prevention (CDC) report, the maximum number of confirmed cases and deaths were reported in Queens County in New York state and King County in Washington state, respectively. The first COVID-19 case in the United States was confirmed on Jan 19, 2020, in Washington State. Subsequently, New York City became one of the epicenters of the disease and on Mar 17, 2020, all fifty states across the United States had at least one confirmed case of COVID-19. On Mar 26, 2020, the United States became the leading country in the number of COVID-19 cases worldwide, replacing Italy that was previously in the lead of COVID-19 cases (Center for Infectious Disease Research and Policy, 2020, https://www.cidrap.umn.edu/).

The U.S. descended from the 1st peak on Apr 7, 2020, with 31,865 new cases per day to 17 230 new cases per day on Jun 8 (see the 7-day moving average graph of new cases, Mar to Nov 2020, on https://coronavirus.jhu.edu/data/new-cases and https://covidtracking.com/data/charts/us-daily-positive). During the 1st wave, most cases were concentrated in New York and other American Northeast states (https://www.worldometers.info/coronavirus/country/us/). The mitigation strategies to reduce disease transmission during this wave included shelter in place, mask-wearing, hand washing, distancing, crowd avoidance (such as restaurants) and cancelation of social activities^2^. During summer, COVID-19 cases had another spike on July 24, 2020 (74,857 new cases per day) before decreasing once more to 33 621 new cases per day (on Sep 14, 2020). During this wave (2nd wave, Jul–Sep 2020), most cases were concentrated in states in the southern US, the so-called sunbelt region. The 3rd wave started in mid-September, where cases had another massive spike in mid-November, 2020 (192,805 new cases per day). Most of the cases during this period were concentrated in states located in the great plains. During the 2nd and 3rd waves, the same mitigation measures followed. However, mask-wearing increased over time with many state and local governements issuing such orders, but strategies focusing on crowd avoidance such as in-dining options decreased^2^. Further, many Universities and Colleges offered on campus teaching options.

Studies have reported multiple risk factors for COVID-19 mainly categorized into three groups: (1) comorbidities (including chronic lung disease, heart diseases, diabetes, cancer, and chronic liver disease), (2) demographics & social factors (including age, gender, ethnicity, and smoking status), and (3) environmental factors (including temperature, humidity, and air pollution). Understanding the associated risk factors can aid in future healthcare planning on where to dedicate additional and subject-specific resources for vulnerable people and also areas. Despite numerous claims in the literature of the significant role that pre-existing conditions play, the studies to date are not conclusive given the fast-changing landscape of data and the current understanding of the disease. Moreover, to the best of our knowledge, longitudinal model-based clustering using the disease mortality pattern over time has not yet been considered in published studies. Hence, this study using an appropriate modeling framework contributes to the literature by finding relevant clusters considering disease growth trajectories. To this end, we first determined the county-level risk factors of COVID-19 MIR in the United States using a longitudinal generalized estimating equations (GEE) model. Next, we trained a latent growth mixture model (LGMM) to cluster the U.S. counties and to identify significant risk factors for each cluster separately. This longitudinal model-based clustering approach enables us to incorporate the possible heterogeneity of COVID-19 MIR growth trajectories present due to the previously mentioned factors. Note that such heterogeneity is not accounted in other simpler, but widely used models, such as the SIR (susceptible, infected, and recovered) model and its variants. Our methodology enables us to cluster different counties into distinctive subpopulations based on their similarities in COVID-19 patterns over time (Mar 25–Nov 12, 2020).

The proposed methodology aids in understanding the evolution of COVID-19 disease transmission and severity by examining MIR and developing a model-based clustering system that takes into consideration both disease patterns over time and pre-existing risk factors. Identifying disease-specific clusters of vulnerable communities and risk factors provides insights for public health policymakers on the groups of people and locations that require attention both in terms of resources and mitigation strategies. Finally, the methodology is readily applicable to other countries if similar granularity data are available.

Next, we review the primarily published evidence reporting associations between the above mentioned risk factors and COVID-19 incidence, mortality, and severity. We consider more severely impacted patients from COVID-19, those in need of requiring oxygen, hospitalization, or ventilation. A more exhaustive literature review is available in the Supplement.

### Comorbidities

#### Chronic lung diseases, CLD

COVID-19 is an acute respiratory disease that primarily affects the pulmonary alveolar epithelial cells, which can lead to respiratory failure and death^3^. There are different hypotheses about whether people with pre-existing CLD (especially chronic obstructive pulmonary disease, COPD) would be at a higher risk of infection with the SARS-CoV-2 virus and representing more severe symptoms than others.

Halpin et al.^4^ showed that the CLD prevalence among COVID-19 cases was less than the general population's estimated prevalence^4^. In a study from Italy (Mar 23, 2020), COPD was not reported for any of the patients who died from COVID-19 (n = 355, mean-age = 79.5)^5^. Similarly, in data from the US (Mar 31, 2020), chronic respiratory diseases were comorbidities in 8.5% of patients with COVID-19, compare to the Global Burden of Disease (GBD) estimate of 11.3% for the same disease^6^. Several published studies show the synergistic effect of CLD in worsening the severity of COVID-19^7–11^. Guan et al. reported more than 50% of chronic pulmonary disease presence for COVID-19 patients admitted to the ICU^12^. In a meta-analysis study on both Chinese- and English-language published articles, Zhao et al. showed that pre-existing COPD was significantly associated with a nearly fourfold higher risk of developing severe COVID-19. The association remained significant in the subgroup of patients with a death outcome or requiring ICU admission^8^. Moreover, in large case-series, they reported a higher prevalence of COPD in patients with severe presentation and worse outcomes^9^. In another meta-analysis (May 1, 2020), the reported prevalence of COPD patients was 2% in COVID-19 cases. They showed that although the COPD prevalence was low, it was significantly associated with a higher risk of more severe COVID-19 (63%) and higher mortality (60%)^13^. Brake et al. reported higher (upregulated) expression of the angiotensin-converting enzyme 2 (ACE2) in resected lung tissue from COPD patients compared to those with healthy lung function^10^. Some published evidence also indicates higher ACE2 expression in smokers compared to never smokers, which suggests that smokers can be more susceptible to infection by the SARS-CoV-2 virus^10,11^.

It is necessary to put all these findings into context and consider that people with CLD, especially past or current smokers, are more likely to have immune dysregulation. Therefore, these groups of people can be at higher risk of developing more severe symptoms out of a simple upper respiratory infection (similar to the Bhat et al. suggestion^14^).

#### Cardiovascular disease, CVD

In addition to respiratory complications, published studies are showing the impact of pre-exist CVDs on developing COVID-19 and on worsening its severity and clinical outcomes. Hendren et al. showed that COVID-19 might cause myocarditis-like syndrome and acute myocardial injury associated with reduced left ventricular ejection fraction (LVEF), which can also be complicated by heart failure^15^. A different analysis based on Chinese data showed that 8–20% of the patients hospitalized with COVID-19 had abnormal cardiac troponin I (cTnI), were also older and had more comorbid diseases^16,17^. There is also published literature suggesting that SARS-CoV-2 can infect fibroblasts and cardiomyocytes via the ACE2-pathway causing myocardial injury^18–22^. Moreover, it is shown that patients with viral myocarditis, which commonly exhibit chest pains, can mimic ventricular arrhythmia or coronary syndrome^23,24^. Historically, research has shown a significant increase in SARS patients' mortality with pre-existing CVD^25–30^.

### Demographic and social factors

#### Age

People 65 years of age and older are at significantly higher risk of experiencing COVID-19 or hospitalization and death, especially if they have pre-existing comorbidities such as CVD, DM, CLD, Hypertensive heart disease, and obesity^31,32^. Ferguson et al. reported that 27–71% of patients older than 60 years needed special care in an ICU with an infection fatality rate of about 2–9.5%^33,34^. Stang et al. discussed a potential bias in age-significance in COVID-19 patients due to overestimation caused by the limited testing capacity to more symptomatic patients. They showed that the fatality rate from COVID-19 started increasing after the age of 60 years in Italy, Spain, and the USA^35,36^. There is also a study on children with a median age of 7 years in China (April 1, 2020) in which most of the cases were male (not significant, though) with mild symptoms^37^. Note that the evidence and data to confirm whether increase in mortality is directly related to age or other confounders related to age is still rather mixed. For instance, Starke et al.^38^ showed that when adjusting for other comorbidities, there is no additional risk of death by age. Other similar studies in Austria^39^ and Italy^40^ support the insignificant effect of age on COVID-19 severity and mortality rate, after accounting for other factors. However, recent studies in the USA (New York)^41^ and Brazil (Espírito Santo)^42^ showed an increase in COVID-19 mortality (OR 6.3% in Brazil and OR 1.7 in the USA).

#### Gender

Most evidence suggests that men are infected at a higher rate than women by COVID-19 and exhibit a higher mortality rate. However, most studies showed no significant differences in infection and mortality between men and women in COVID-19 cases^3,43^. Wenham et al. indicated that although an equal number of male and female COVID-19 cases was observed, MR is different by gender. Wenham et al. also suggested that women can be at high risk of getting infected since they have more front-line interaction with communities and provide more informal care within families besides their physical and cultural differences^44,45^.

Further, selected studies report significantly different gender-distributions between male and female COVID-19 cases. For example, Zhao Y et al., using single-cell data, reported that ACE2 was upregulated in Asian males compared to women and other ethnicities, which may lead to more severe incidents of COVID-19^11,46–49^.

### Environmental factors

#### Air pollution

Exposure to air pollution and particulate matter (PM) can have a positive association with increased risk of certain viral respiratory diseases such as influenza and SARS pandemic 2003. Studies show that exposure to PM increased the MR from 2009 H1N1 and Spanish influenza^50–53^. Air pollution is also linked to cellular damage, inflammation, CVD, and CLD, which are potential comorbidities associated with COVID-19 severity^50,54–56^. Ye et al. showed that air pollution could also play a role in infectious disease transmission, although it has not been studied for COVID-19 as of May 15, 2020^57^.

Wu et al. and Mollalo et al., in nationwide studies in the USA, showed that exposure to PM increased COVID-19 mortality and severity^50,58,59^. Setti et al. reported a significant relationship between PM and experiencing COVID-19 in Italy (Jan 1, 2020)^60^.

A number of studies did not confirm the association between air pollution and COVID-19 severity, mortality, and transmission. However, they agreed that since exposure to air pollution, and PM has a link with other complications, there can be a risk factor in increasing COVID-19 MR and disease severity^61–64^.

## Methods

### Data resources

We collected county-level cumulative COVID-19 confirmed cases and death from Mar 25 to Nov 12, 2020, across the contiguous United States from *USAFacts* (usafacts.org). As explained in the introductory section, we considered Mar 25 to Jun 3 as the “1st wave”, Jun 4 to Sep 2 as the “2nd wave”, and Sep 3 to Nov 12 as the “3rd wave” of COVID-19. For the 2nd and 3rd waves, we analyzed targeted counties in the sunbelt region (including AL, AZ, AR, CA, FL, GA, KS, LA, MS, NV, NM, NC, OK, SC, TX, TN, and UT states) and the great plains region (including IA, IL, IN, KS, MI, MO, MN, ND, NE, OH, SD, and WI states), respectively. MIR, as a proxy for survival rate, is calculated by dividing the number of confirmed deaths in each county by the confirmed cases in the same county at the same time-period multiplied by 100. MIR ranges from 0 to 100%, 100% indicating the worst situation where all confirmed cases have died.

Thirty-eight potential risk factors (covariates), including county-level MR of comorbidities & disorders, demographics & social factors, and environmental factors, were retrieved from the *University of Washington Global Health Data Exchange* (http://ghdx.healthdata.org/us-data). Comorbidities and disorders include CVD, cardiomyopathy and myocarditis and myocarditis, hypertensive heart disease, peripheral vascular disease, atrial fibrillation, cerebrovascular disease, diabetes, hepatitis, HIV/AIDS, tuberculosis (TB), lower respiratory infection, interstitial lung disease and pulmonary sarcoidosis, asthma, COPD, ischemia, mesothelioma, tracheal cancer, leukemia, pancreatic cancer, rheumatic disease, drug use disorder, and alcohol use disorder. Demographics & social factors include age, female African American%, female white American%, male African American%, male white American%, Asian%, smokers%, unemployed%, income rate, food insecurity, fair/poor health, and uninsured%. Environmental factors include county population density, air quality index (AQI), temperature, and PM. A descriptive table, including all potential risk factors, is provided in Table S1).

### Analysis (descriptive methods and models)

We **first** provide summary statistics for COVID-19 data for the period under consideration. Full descriptive statistics for n = 38 potential risk factors are provided in Table S1in the Supplement.

**Second**, we applied GEE marginal approaches to model the COVID-19 MIR over time and found significant risk factors. To this end, we first used the forward-selection method to select the most relevant risk factors (covariates) among the covariates using univariate GEE models^65^, as follows:

1 $$ \left\{ {\begin{array}{*{20}l} {\mu_{ij}^{\left( 1 \right)} = \beta_{0} + \beta_{1} Time + \beta_{2\left( 1 \right)} X_{\left( 1 \right)} } \hfill & {} \hfill \\ {\mu_{ij}^{\left( 2 \right)} = \beta_{0} + \beta_{1} Time + \beta_{2\left( 2 \right)} X_{\left( 2 \right)} , } \hfill & { i = 1, \ldots I \left( {counties} \right);\quad j = 1, \ldots ,J \left( {weeks} \right).} \hfill \\ \vdots \hfill & {} \hfill \\ {\mu_{ij}^{{\left( {38} \right)}} = \beta_{0} + \beta_{1} Time + \beta_{{2\left( {38} \right)}} X_{{\left( {38} \right)}} } \hfill & {} \hfill \\ \end{array} } \right. $$

where $\mu_{ij}$ indicates the mean COVID-19 MIR for the $i^{th}$ county in week $j^{th}$, $\beta_{0}$ is the starting rate of MIR before considering the effect of any potential risk factor (intercept), ${\beta }_{1}$ and ${\beta }_{2}$ s are the effects of time and risk factors $X$ (such as Asthma) on the COVID-19 MIR. For variable selection purposes, we chose variables with (univariate) *P* value < 0.2 to be included in the final multivariate GEE model, as follows:

2 $${\mu }_{ij}={\alpha }_{0}+\sum_{p=1}^{{n}_{1}}{{\alpha }_{p}X}_{p},$$

where ${\mu }_{ij}$ indicates the overall marginal mean MIR for the ${i}^{th}$ county in the ${j}^{th}$ week. ${\alpha }_{0}$ is the intercept and ${\alpha }_{p}$ is the coefficient of the ${p}^{th}$ potential risk factor (${X}_{p}$), $p=\mathrm{1,2},\dots ,{n}_{1}$, where ${n}_{1}$ is the total number of the selected variables based on the univariate GEE model (Eq. 1). Variables with (multivariate) *P* value < 0.05 will be selected as the potential risk factors. In each marginal model, an appropriate correlation structure (with the best goodness of fit index, QIC) was utilized. Statistical analysis and visualization for this step were performed using the *geepack* R-package (https://cran.r-project.org/web/packages/geepack/).

**Third**, we evaluated COVID-19 MIR growth trajectories over the study time periods (1st, 2nd, and 3rd waves) using a latent growth model (LGM). An LGM approach considers both the mean MIR differences between counties at each time point (inter-subject) and MIR growth trajectories over time (intra-subject). Specifically, suppose ${y}_{ti}$ is the COVID-19 MIR in the ${i}^{th}$ county at time $t$; then, it can be modeled as follows^66^:

3 $$ \begin{aligned} y_{ti} & = \eta_{0i} + \eta_{1i} \lambda_{t} + \varepsilon_{ti} , \\ \eta_{0i} & = \eta_{0} + \varepsilon_{0i} , \\ \eta_{1i} & = \eta_{1} + \varepsilon_{1i} , \\ \end{aligned} $$

where ${\eta }_{0i}$ and ${\eta }_{1i}$ are two latent growth factors and ${\lambda }_{t}$ s are time scores (factor loadings); ${\varepsilon }_{ti}$ is a normally distributed error term for the ${i}^{th}$ county at time $t$; ${\eta }_{0}$ and ${\eta }_{1}$ indicate the estimated overall mean COVID-19 MIR in each county and the average rate of MIR change, respectively. We also employed a number of non-linear (quadratic) LGMs, based on a polynomial time function (quadratic or higher-order) of time scores^67^ to decrease estimation bias to account for the MIR trajectories exhibiting non-linear behavior over time. The non-linear LGM using a quadratic time function is given by:

4 $$ \begin{aligned} y_{ti} & = \eta_{0i} + \eta_{1i} \lambda_{t} + \eta_{2i} \lambda_{t}^{2} + \varepsilon_{ti} , \\ \eta_{0i} & = \eta_{0} + \varepsilon_{0i} , \\ \eta_{1i} & = \eta_{1} + \varepsilon_{1i} , \\ \eta_{2i} & = \eta_{2} + \varepsilon_{2i} , \\ \end{aligned} $$

where ${\eta }_{2}$ indicates the growth factor, which can be a concave or convex form of the COVID-19 MIR pattern over the study time periods (1st, 2nd, and 3rd waves), and ${\uplambda }_{t}^{2}$ are the squared time scores. Both linear and non-linear LGMs were applied to 1736 U.S. counties with MIR > 0, i.e., counties with at least one confirmed death between Mar 25 to Nov 12, 2020. We then used information criteria (AIC, BIC) to find the best model among linear and non-linear LGMs to determine the COVID-19 MIR changes and patterns over the study time. Smaller AIC and BIC values indicate a better fit of the underlying models. We also calculated *Moran’s I*^68^ to evaluate the spatial autocorrelation of COVID-19 MIR across the U.S. counties.

**Fourth**, we identified clusters of the U.S. counties based on the COVID-19 MIR growth trajectory over time using longitudinal LGMMs^66^, as follows:

5 $$ \begin{aligned} y_{it}^{k} & = \eta_{i0}^{k} + \eta_{i1}^{k} \lambda_{t}^{k} + \varepsilon_{it}^{k} , \\ \eta_{i0}^{k} & = \eta_{00}^{k} + \varepsilon_{i0}^{k} , \\ \eta_{i1}^{k} & = \eta_{10}^{k} + \varepsilon_{i1}^{k} , \\ \end{aligned} $$

where $k$ is the upper bound of the number of the clusters, ${\eta }_{00}^{k}$ indicates the initial COVID-19 MIR at the beginning of the study, and ${\eta }_{10}^{k}$ indicates the average rate of COVID-19 MIR change over time. To find the optimal number of clusters ($k$), we fit a series of LGMMs with different numbers of clusters of counties and conducted tests for the adequacy of the reduced models with respect to the number of clusters. Information criteria such as AIC, BIC, and a bootstrap likelihood ratio test (BLRT) were used to compare the $k$-cluster model to the $(k-1)$-clsuter model^69,70^. Also, cluster sample sizes greater than 1% of the total sample size and a relative entropy (REN) statistic greater than 0.8 were considered as the qualified latent class membership classification criteria^71^. The REN statistic for a $k$-class model is calculated as $REN(k)=1-\frac{-\sum_{i=1}^{N}\sum_{k=1}^{K}{P}_{ik}ln{P}_{ik}}{N-lnK}$, where $k$ and $i$ correspond to the number of clusters and counties, respectively, and ${P}_{ik}$ indicates the posterior probability for the ${i}^{th}$ county to be in cluster $k$. We then applied a multinomial logit model to find the significant risk factors in each cluster as follows:

6 $$ ln\frac{{p\left( {y_{i} = k} \right)}}{{p\left( {y_{i} = 0} \right)}} = \alpha_{k} + \mathop \sum \limits_{p = 1}^{{n_{1} }} \beta_{p} X_{p} , \quad k = 1, \ldots ,K \left( {cluster} \right) $$

where $y_{i}$ is a categorical variable with $K$ possible categories (indicating the cluster number), ${\alpha }_{k}$ is the intercept for cluster $k$, ${\beta }_{k}$ is a vector of regression coefficients of the ${p}^{th}$ potential risk factor (${X}_{p}$), $p=\mathrm{1,2},\dots ,{n}_{1}$, where ${n}_{1}$ is the total number of the selected variables based on the univariate GEE model (Eq. 1).

Statistical analysis for LGMMs and multinomial logit model were performed using *Mplus* v6.12 (Muthén & Muthén, CA, USA, www.statmodel.com) and the *nnet* R-package (https://cran.r-project.org/web/packages/nnet/index.html), respectively. The clusters' geographical distribution was illustrated in a color-coded geographical map using *ArcGIS 10.7* (ESRI, Redland, CA).

## Results

During the **1st wave**, the mean COVID-19 MIR in the contiguous United States significantly increased (*P* value < 0.001) from MIR = 0.8% on Mar 25 to MIR = 3.0% on April 22 (Table 1). Henceforth, the rate slightly increased (*P* value = 0.501) to MIR = 3.2% on April 29 and remained at this level until Jun 3, 2020 (Table 1). During the **2nd wave**, for the targeted counties (counties in the states of AL, AZ, AR, CA, FL, GA, KS, LA, MS, NV, NM, NC, OK, SC, TX, TN, and UT), there were two significant decreases in the mean COVID-19 MIR from Jun 25 to Jul 2 (MIR = 2.8% to MIR = 2.4%, *P* value = 0.031), and from Jul 2 to Jul 9 (MIR = 2.4% to MIR = 2.2%, *P* value = 0.043). At the beginning of the **3rd wave** (Sep 3), for the targeted counties (counties in the states of IA, IL, IN, KS, MI, MO, MN, ND, NE, OH, SD, and WI), the mean COVID-19 MIR started from MIR = 1.8% and decreased to MIR = 1.6% by Oct 15, 2020. This rate then decreased to MIR = 1.4% by the end of the wave on Nov 12, 2020 (Table 1).

**Table 1:** Descriptive statistics of COVID-19 MIR in the United States for the 1st wave (Mar 25–Jun 3, 2020; n = 3050 counties), the 2nd wave (Jun 4–Sep 2, n = 1344) and the 3rd wave (Sep 3–Nov 12, n = 1055).

Wave	Time*	COVID-19 MIR					*P* value***
		Minimum (N, %)	Maximum (N, %)	Mean (%)	SD (%)	Mean Difference (%)**	
1st	Mar 25	0.0 (2830, 92.8%)	1.0 (9, 0.3%)	0.8	6.5	*NA*	*NA*
	Apr 1	0.0 (2507, 82.2%)	1.0 (11, 0.4%)	1.6	7.5	0.7	** < 0.001**
	Apr 8	0.0 (2185, 71.6%)	1.0 (10, 0.3%)	2.1	7.9	0.5	**0.004**
	Apr 15	0.0 (1936, 63.5%)	1.0 (7, 0.2%)	2.6	6.4	0.5	**0.002**
	Apr 22	0.0 (1763, 57.8%)	1.0 (8, 0.3%)	3.0	6.4	0.4	**0.020**
	Apr 29	0.0 (1643, 53.9%)	1.0 (4, 0.1%)	3.2	5.4	0.1	0.501
	May 6	0.0 (1553, 50.9%)	0.55 (9, 0.3%)	3.2	5.1	0.08	0.600
	May 13	0.0 (1487, 48.8%)	0.50 (3, 0.1%)	3.2	5.2	0.02	0.900
	May 20	0.0 (1417, 46.4%)	1.0 (1, 0.0%)	3.2	5.1	0.02	0.900
	May 27	0.0 (1376, 45.1%)	1.0 (1, 0.0%)	3.2	5.2	− 0.00	0.989
	Jun 3	0.0 (1311, 42.9%)	1.0 (1, 0.0%)	3.2	5.0	− 0.01	0.900
2nd	Jun 4	0.0 (442, 32.9%)	0.5 (2, 0.1%)	3.3	4.6	*NA*	*NA*
	Jun 11	0.0 (426, 31.7%)	0.5 (3, 0.2%)	3.2	4.5	− 0.1	0.488
	Jun 18	0.0 (406, 30.2%)	0.5 (3, 0.2%)	3.0	4.3	− 0.2	0.267
	Jun 25	0.0 (399, 29.7%)	0.5 (3, 0.2%)	2.8	4.2	− 0.3	0.128
	Jul 2	0.0 (386, 28.7%)	0.5 (2, 0.1%)	2.4	3.7	− 0.3	**0.031**
	Jul 9	0.0 (368, 27.4%)	0.5 (2, 0.1%)	2.2	3.3	− 0.3	**0.043**
	Jul 16	0.0 (350, 26.0%)	0.5 (1, 0.1%)	2.0	2.9	− 0.2	0.074
	Jul 23	0.0 (318, 23.7%)	0.3 (1, 0.1%)	1.8	2.4	− 0.1	0.190
	Jul 30	0.0 (249, 18.5%)	0.2 (2, 0.1%)	1.9	2.4	0.1	0.292
	Aug 6	0.0 (222, 16.5%)	0.5 (1, 0.1%)	2.0	2.6	0.1	0.615
	Aug 13	0.0 (195, 14.5%)	0.5 (1, 0.1%)	2.1	2.5	0.1	0.351
	Aug 20	0.0 (181, 13.5%)	0.5 (1, 0.1%)	2.1	2.5	0.05	0.577
	Aug 27	0.0 (165, 12.3%)	0.5 (1, 0.1%)	2.2	2.4	0.1	0.476
	Sep 2	0.0 (150, 11.2%)	0.4 (1, 0.1%)	2.2	2.2	0.02	0.788
3rd	Sep 3	0.0 (320, 30.3%)	0.2 (1, 0.1%)	1.8	2.3	*NA*	*NA*
	Sep 10	0.0 (296, 28.1%)	0.3 (1, 0.1%)	1.8	2.3	0.0	0.933
	Sep 17	0.0 (284, 26.9%)	0.3 (1, 0.1%)	1.7	2.2	− 0.1	0.578
	Sep 24	0.0 (263, 24.9%)	0.3 (1, 0.1%)	1.7	2.2	0.0	0.812
	Oct 1	0.0 (235, 22.3%)	0.3 (1, 0.1%)	1.7	2.1	0.0	0.916
	Oct 8	0.0 (219, 20.8%)	0.3 (1, 0.1%)	1.7	2.0	0.0	0.736
	Oct 15	0.0 (188, 17.8%)	0.3 (1, 0.1%)	1.6	1.9	0.0	0.674
	Oct 22	0.0 (167, 15.8%)	0.2 (1, 0.1%)	1.6	1.7	0.0	0.849
	Oct 29	0.0 (144, 13.6%)	0.2 (1, 0.1%)	1.6	1.6	0.0	0.520
	Nov 5	0.0 (119, 11.3%)	0.2 (1, 0.1%)	1.5	1.4	− 0.1	0.376
	Nov 12	0.0 (105, 10.0%)	0.2 (1, 0.1%)	1.4	1.3	− 0.1	0.237

*Year of 2020.

**Mean difference between mean COVID-19 MIR at each time and the previous time.

** *P* values from the *t* test comparing mean COVID-19 MIR in each time with the previous time.

Bold values are statistically significant for *P* < 0.05.

At the beginning of the **1st wave** (Mar 25), about 93% (n = 2830) of the U.S. counties had zero confirmed death (MIR = 0%), which decreased to 42.9% (n = 1311) by the end of the 1st wave (Jun 3, 2020). This percentage at the beginning of the **2nd wave (Jun 4) for the states under consideration** was 32.9% (n = 442) and decreased to 11.2% (n = 150) by the end of this wave (Sep2, 2020). During the **3rd wave for the states under consideration**, this rate started from 30.3% (n = 320) and decreased to 10.0% (n = 105).

On Jun 3, 2020, the median population of the 3050 U.S. counties was 25 884, with Loving county in Texas having the smallest population (n = 169) and Los Angeles County in California the largest one (n = 1,039,107). Queens County in New York state had the maximum number of confirmed cases at the beginning of the study on Mar 25 (n = 6,420), while Cook County in Illinois had the maximum confirmed cases (n = 80,204) at the end of the 1st wave on Jun 3, 2020; whereas the maximum number of confirmed death was reported in King County in Washington state on Mar 25 (n = 100) and in Kings County in New York state on Jun 3 (n = 6,774). On Jun 4 (the beginning of the **2nd wave for the states under consideration)**, Los Angeles County in CA had the maximum number of both confirmed cases (n = 59,650) and deaths (n = 2,531). This county had the maximum number of both confirmed cases (n = 243,935) and deaths (n = 5,878) at the end of the 2nd wave (Sep 2), as well. During the **3rd wave for the states under consideration**, Cook County in IL had the maximum number of cases (n = 128,012 on Sep 3 and n = 227,425 on Nov12) and deaths (n = 5,080 on Sep 3 and n = 5,667 on Nov 12).

Based on the univariate variable selection method (Table 2), some potential risk factors were excluded from the final analysis. The description table of the potential risk factors is provided in Table S1 in the Supplement.

**Table 2:** Univariate GEE variable selection results. COVID-19 MIR risk factors based on univariate longitudinal GEE models (Mar 25 to Nov 12, 2020, USA).

Variable	1st wave		2nd wave		3rd wave	
	Est. (%)	*P* value*	Est. (%)	*P* value	Est. (%)	*P* value
Time	0.21	** < 0.001**	0.03	** < 0.001**	− 0.03	** < 0.001**
**Comorbidities and disorders**						
CVD	0.004	**0.003**	0.005	** < 0.001**	0.01	** < 0.001**
Cardiomyopathy and myocarditis	0.21	** < 0.001**	0.13	** < 0.001**	0.12	** < 0.001**
Hypertensive heart disease	0.06	** < 0.001**	0.05	** < 0.001**	0.05	** < 0.001**
Peripheral vascular disease	0.42	**0.008**	0.29	**0.010**	0.37	** < 0.001**
Atrial fibrillation	− 0.14	** < 0.001**	− 0.20	** < 0.001**	0.01	0.788
Cerebrovascular disease	0.01	**0.100**	0.01	**0.073**	0.01	**0.055**
Diabetes	0.08	** < 0.001**	0.07	**0.001**	0.08	** < 0.001**
Hepatitis	0.72	**0.158**	− 0.31	**0.146**	4.54	** < 0.001**
HIV/AIDS	0.23	**0.022**	0.17	**0.046**	0.83	** < 0.001**
TB	2.02	** < 0.001**	3.45	** < 0.001**	0.38	**0.196**
Lower respiratory infection	0.02	**0.026**	0.03	** < 0.001**	0.02	** < 0.001**
Interstitial lung disease and pulmonary sarcoidosis	0.23	** < 0.001**	0.04	0.544	0.19	** < 0.001**
Asthma	0.005	0.980	0.93	** < 0.001**	− 0.60	**0.003**
COPD	0.002	0.703	0.002	0.626	0.03	** < 0.001**
Ischemia	0.002	**0.074**	0.004	**0.030**	0.01	** < 0.001**
Mesothelioma	0.83	**0.002**	− 0.44	**0.096**	0.72	**0.003**
Tracheal cancer	0.02	** < 0.001**	0.01	**0.144**	0.02	** < 0.001**
Leukemia	0.08	0.402	− 0.05	0.619	0.12	**0.086**
Pancreatic cancer	0.48	** < 0.001**	0.28	** < 0.001**	0.35	** < 0.001**
Rheumatic disease	0.02	0.774	0.42	** < 0.001**	− 0.10	0.307
Drug use disorder	0.06	** < 0.001**	− 0.01	0.306	0.08	** < 0.001**
Alcohol use disorder	− 0.08	** < 0.001**	0.03	0.344	− 0.03	**0.137**
**Demographics and social**						
Age	− 0.34	0.230	0.07	**0.039**	0.0001	0.952
Female-AA%	3.82	** < 0.001**	3.58	** < 0.001**	5.68	** < 0.001**
Female-WA%	− 3.21	** < 0.001**	− 3.80	** < 0.001**	− 0.66	**0.143**
Male-AA%	3.86	** < 0.001**	3.71	** < 0.001**	4.38	** < 0.001**
Male-WA%	− 3.26	** < 0.001**	− 3.92	** < 0.001**	− 0.41	0.367
Asian%	0.13	** < 0.001**	− 0.03	**0.012**	0.02	0.444
Smokers%	0.07	**0.004**	0.09	** < 0.001**	0.07	** < 0.001**
Unemployed%	0.18	** < 0.001**	0.24	** < 0.001**	0.21	** < 0.001**
Income rate	0.40	** < 0.001**	0.66	** < 0.001**	0.13	**0.128**
Uninsured%	− 0.04	**0.008**	0.06	**0.004**	− 0.03	**0.012**
Food insecurity	0.08	** < 0.001**	0.13	** < 0.001**	0.07	** < 0.001**
Fair/poor health	0.03	**0.124**	0.08	** < 0.001**	0.05	** < 0.001**
**Environmental**						
Population density	0.004	** < 0.001**	0.0002	**0.121**	0.0001	** < 0.001**
AQI	0.10	** < 0.001**	0.03	**0.028**	0.04	** < 0.001**
Temperature	0.04	** < 0.001**	0.005	** < 0.001**	0.06	** < 0.001**
PM	0.38	** < 0.001**	0.11	**0.050**	0.12	**0.002**

* *P* value < 0.2 is considered as significant.

Bold values are statistically significant for *P* < 0.05.

Results of the final multivariate GEE model for the **1st wave** (Table 3) showed significant positive associations between COVID-19 MIR and cardiomyopathy and myocarditis (β = 0.15%, *P* value < 0.001), hypertensive heart disease (β = 0.11%, *P* value = 0.001), peripheral vascular disease (β = 0.31%, *P* value = 0.038), cerebrovascular disease (β = 0.07%, *P* value = 0.034), ischemia (β = 0.08%, *P* value = 0.017), mesothelioma (β = 0.58%, *P* value = 0.031), pancreatic cancer (β = 0.52%, *P* value < 0.001), drug use disorder (β = 0.08%, *P* value < 0.001), and smokers% (β = 0.11%, *P* value = 0.019). Whereas, there were negative associations between COVID-19 MIR and CVD (β =  − 0.08%, *P* value = 0.011), tracheal cancer (β =  − 0.03%, *P* value < 0.001), alcohol use disorder (β =  − 0.17%, *P* value = 0.002), and fair/poor health (β =  − 0.09%, *P* value = 0.024).

**Table 3:** Multivariate GEE mo**del’s results. COVID-19 MIR risk factors based on a multivariate longitudinal GEE model (Mar 25 to Nov 12, 2020, USA).

Variable	1st wave		2nd wave		3rd wave	
	Est. (%)	*P* value*	Est. (%)	*P* value	Est. (%)	*P* value
Time	0.01	0.501	− 0.09	** < 0.001**	− 0.03	** < 0.001**
**Comorbidities and disorders**						
CVD	− 0.08	**0.011**	− 0.06	**0.036**	0.01	0.768
Cardiomyopathy and myocarditis	0.15	** < 0.001**	0.12	**0.004**	0.00	0.865
Hypertensive heart disease	0.11	**0.001**	0.09	**0.005**	0.02	0.461
Peripheral vascular disease	0.31	**0.038**	0.13	0.321	− 0.07	0.717
Atrial fibrillation	0.00	0.961	− 0.04	0.418	–	–
Cerebrovascular disease	0.07	**0.034**	0.07	**0.025**	− 0.01	0.595
Diabetes	0.02	0.514	− 0.01	0.671	0.04	**0.044**
Hepatitis	− 0.27	0.629	− 0.15	0.704	− 3.33	**0.021**
HIV/AIDS	0.04	0.497	0.09	**0.020**	0.36	0.264
TB	− 0.30	0.666	− 0.30	0.684	0.05	0.951
Lower respiratory infection	0.00	0.976	0.01	0.135	0.01	0.237
Interstitial lung disease and pulmonary sarcoidosis	0.06	0.487	–	–	0.15	**0.046**
Asthma	–	–	− 0.75	**0.011**	− 0.65	**0.029**
COPD	–	–	–	–	0.00	0.890
Ischemia	0.08	**0.017**	0.06	**0.035**	0.00	0.994
Mesothelioma	0.58	**0.031**	− 0.03	0.915	0.32	0.236
Tracheal cancer	− 0.03	** < 0.001**	− 0.02	**0.022**	− 0.02	0.091
Leukemia	–	–	–	–	− 0.03	0.784
Pancreatic cancer	0.52	** < 0.001**	0.13	0.120	0.19	0.061
Rheumatic disease	–	–	0.42	** < 0.001**	–	–
Drug use disorder	0.08	** < 0.001**	−	−	0.02	0.214
Alcohol use disorder	− 0.17	**0.002**	−	−	− 0.08	**0.030**
**Demographics and social**						
Age	–	–	0.12	** < 0.001**	–	–
Female-AA%	12.70	0.241	− 16.20	0.199	6.85	**0.004**
Female-WA%	7.59	0.398	− 23.20	0.095	0.29	0.796
Male-AA%	− 15.90	0.165	15.40	0.243	− 6.88	** < 0.001**
Male-WA%	− 10.90	0.254	20.40	0.165	–	–
Asian%	0.01	0.814	0.02	0.436	–	–
Smokers%	0.11	**0.019**	0.01	0.783	0.08	**0.035**
Unemployed%	0.05	0.466	0.09	0.080	0.12	0.108
Income rate	0.02	0.891	0.13	0.468	0.02	0.559
Uninsured%	0.00	0.864	0.08	**0.002**	0.00	0.840
Food insecurity	0.02	0.580	0.04	0.297	− 0.03	0.324
Fair/poor health	− 0.09	**0.024**	− 0.06	0.074	− 0.09	**0.016**
**Environmental**						
Population density	0.00	0.196	0.0003	**0.011**	0.0001	** < 0.001**
AQI	0.05	0.720	− 0.20	0.072	0.11	0.057
Temperature	0.01	0.501	0.02	0.287	0.03	0.110
PM	0.03	0.953	0.63	0.131	− 0.49	**0.015**

*P value < 0.05 is considered as significant.

Bold values are statistically significant for *P* < 0.05.

During the **2nd wave**, there were positive associations between COVID-19 MIR and cardiomyopathy and myocarditis (β = 0.12%, *P* value = 0.004 ), hypertensive heart disease (β = 0.09%, *P* value = 0.005), cerebrovascular disease (β = 0.07%, *P* value = 0.025), HIV/AIDS (β = 0.09%, *P* value = 0.020), ischemia (β = 0.06%, *P* value = 0.035), rheumatic disease (β = 0.42%, *P* value < 0.001), age (β = 0.12%, *P* value < 0.001), uninsured% (β = 0.08%, *P* value = 0.002), and population density (β = 0.0003%, *P* value = 0.011). Whereas, there were negative associations between COVID-19 MIR and CVD (β =  − 0.06%, *P* value = 0.036), asthma (β =  − 0.75%, *P* value = 0.011), and tracheal cancer (β =  − 0.02%, *P* value = 0.022).

During the **3rd wave**, there were positive associations between COVID-19 MIR and diabetes (β = 0.04%, *P* value = 0.044), interstitial lung disease & pulmonary sarcoidosis (β = 0.15%, *P* value = 0.046), female-AA% (β = 6.85%, *P* value = 0.004), smokers% (β = 0.08%, *P* value = 0.035), and population density (β = 0.0001%, *P* value < 0.001). Whereas, there were negative associations between COVID-19 MIR and hepatitis (β =  − 3.31%, *P* value = 0.021), asthma (β =  − 0.65%, *P* value = 0.029), alcohol use disorder (β =  − 0.08%, *P* value = 0.030), male-AA% (β =  − 6.88%, *P* value < 0.001), fair/poor health (β =  − 0.09%, *P* value = 0.016), and PM (β =  − 0.49%, *P* value = 0.015).

The effect of time on the COVID-19 MIR was significant and negative for both the 2nd (β =  − 0.09, *P* value < 0.001) and the 3rd (β =  − 0.03, *P* value < 0.001) waves, suggesting that the use of longitudinal (repeated measures) approaches instead of cross-sectional studies are more suitable to evaluate the growth trajectory of COVID-19 MIR over time.

**Figure 1:**
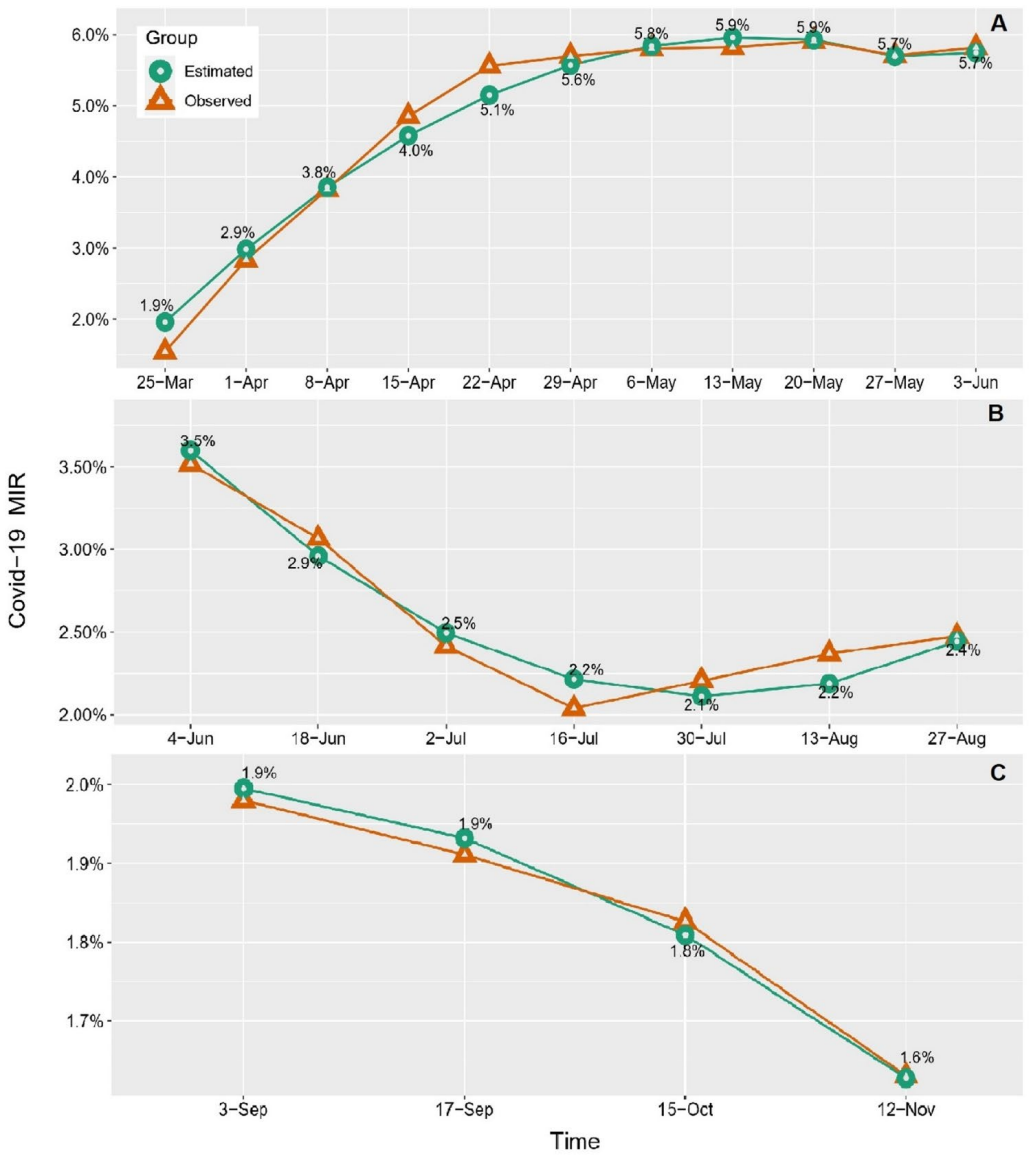
Overall growth trajectories of observed and estimated COVID-19 MIR for the (**A)**. 1st wave, (**B)** 2nd wave, and (**C)** 3rd wave. Green lines indicate the estimated MIR trajectories using an LGM model (linear and non-linear). Orange lines indicate the observed mean MIR.

Tables S2–S4 show the full results based on the LGMs. Based on the information criteria, a non-linear LGM with a quadratic term exhibited a better fit than the linear LGM. Figure 1 shows the overall COVID-19 MIR non-linear growth trajectories for all three waves. The overall growth trajectory of the estimated mean COVID-19 MIR for 1736 U.S. counties (with MIR > 0) during the **1st wave** showed a sharp increase from MIR = 1.9% on Mar 25 to MIR = 5.6% on April 29. Henceforth, the rate slightly increased to MIR = 5.9% on May 20 and then slightly decreased to MIR = 5.7% till Jun 3, 2020 (Fig. 1A, Table S2). During the **2nd wave for the states under consideration**, the estimated mean COVID-19 MIR showed a sharp decrease from MIR = 3.5% on Jun 4 to MIR = 2.1% on Jul 30. Hereafter, the rate slightly increased to MIR = 2.4% till Aug 27, 2020 (Fig. 1B, Table S3). During the **3rd wave for the states under consideration**, the mean COVID-19 MIR started from MIR = 1.9% on Sep 3 and decreased to MIR = 1.6% till Nov 12, 2020 (Fig. 1C, Table S4). Note that for the targeted counties (great plains) during the 3rd wave, the mean COVID-19 MIR was already elevated, therefore, we observe a constant decreas in the growth trajectory. Moreover, the 3rd wave is still in progress at the time that marks the end of our observation period (Nov 2020).

A clustered pattern of COVID-19 MIR across the U.S. is confirmed by *Moran’s I* statistics (**1st wave**: MIR-*Morans’I* = 0.46, *P* value < 0.001; **2nd wave**: MIR-*Morans’I* = 0.38, *P* value < 0.001; **3rd wave**: MIR-*Morans’I* = 0.41, *P* value < 0.001).

**Figure 2:**
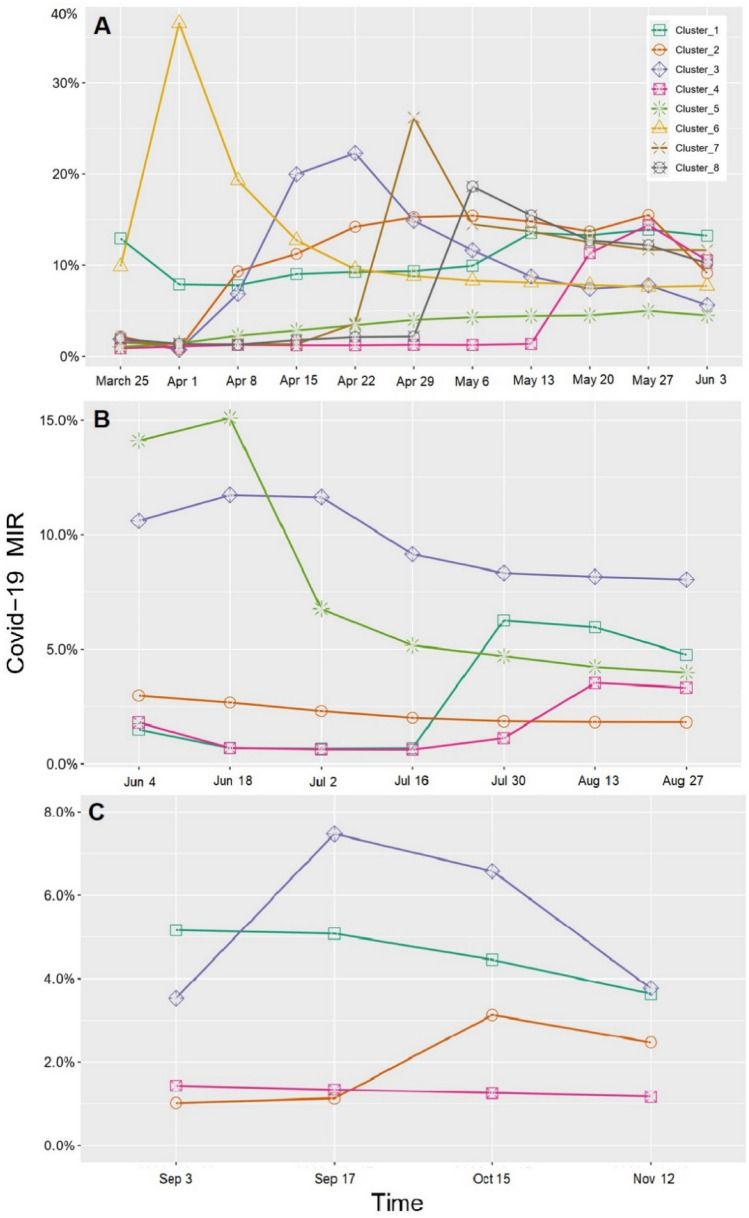
Estimated mean COVID-19 MIR growth trajectories for (**A)** 8 clusters of the U.S. counties during the 1st wave (Mar 25–Jun 3, 2020), (**B)** 5 clusters of the targeted U.S. counties (sunbelt region) during the 2nd wave (Jun 4–Sep 2, 2020), and C. 4 cluster of the targeted U.S. counties (great plains) during the 3rd wave (Sep 3–Nov 12, 2020).

Based on the LGMM results, an 8-cluster non-linear model for the 1st wave, a 5-cluster non-linear model for the 2nd wave, and a 4-cluster non-linear model for the 3rd wave were selected as the best models to find clusters of the U.S. counties. Detailed results for the LGMM models are provided in tables S5–S9. Table 4 and Fig. 2 show the detailed MIR information over time (factor loadings are reported in Table S6).

**Table 4:** GLMM clustering results. Clustering (based on COVID-19 MIR > 0) of the 1736 counties during the 1st wave (Mar 25–Jun 3, 2020), 1344 targeted counties (sunbelt region) during the 2nd wave (Jun 4–Sep 2, 2020), and 1055 targeted counties (great plains region) during the 3rd wave (Sep 3–Nov 12, 2020), USA.

Wave	Cluster	Cluster size N (%)	Intercept*		Slope**	
			Mean (SE)	*P* value	Mean (SE)	*P* value
1st	0	1314 (43.1%)	0% (0%)	*NA*	0% (0%)	*NA*
	1	52 (1.7%)	12.9% (3.1%)	< 0.001	− 1.0% (0.6%)	0.122
	2	74 (2.4%)	2.2% (0.8%)	0.010	3.5% (1.0%)	< 0.001
	3	66 (2.1%)	1.9% (0.9%)	0.027	2.8% (0.4%)	< 0.001
	4	39 (1.3%)	0.9% (0.5%)	0.089	2.0% (0.4%)	< 0.001
	5	1406 (46.1%)	1.0% (0.3%)	< 0.001	− 3.0% (0.5%)	< 0.001
	6	64 (2.1%)	9.8% (3.0%)	0.001	3.4% (0.7%)	< 0.001
	7	12 (0.4%)	1.5% (1.3%)	0.236	− 3.1% (0.5%)	< 0.001
	8	23 (0.8%)	1.9% (1.3%)	0.127	− 4.2% (0.0%)	* − *
2nd	0	156 (11.6%)	0% (0%)	*NA*	0% (0%)	*NA*
	1	32 (2.4%)	1.5% (0.3%)	< 0.001	10.6% (4.5%)	0.018
	2	1035 (77.0%)	3.0% (0.2%)	< 0.001	12.5% (4.3%)	0.004
	3	43 (3.2%)	10.6% (1.8%)	< 0.001	20.7% (12.7%)	0.102
	4	59 (4.4%)	1.8% (0.3%)	< 0.001	16.3% (6.5%)	0.012
	5	19 (1.4%)	14.1% (4.5%)	0.002	74.7% (0.0%)	* − *
3rd	0	111 (10.5%)	0% (0%)	*NA*	0% (0%)	*NA*
	1	125 (11.8%)	5.2% (0.2%)	< 0.001	− 3.7% (0.5%)	< 0.001
	2	47 (4.5%)	1.0% (0.6%)	0.082	3.1% (1.8%)	0.088
	3	11 (1.0%)	3.5% (1.1%)	0.002	− 20.0% (3.2%)	< 0.001
	4	761 (72.2%)	1.4% (0.1%)	< 0.001	− 0.9% (0.3%)	0.001

* Intercept indicates the estimated mean MIR of COVID-19 at the beginning of the wave, for each cluster.

** Slope indicates the overall change of MIR of COVID-19 during each wave, for each cluster.

Details of the nine clusters (including a cluster of counties with zero MIR) during the **1st wave** are as follows: **Cluster 0** contains 1314 counties with zero confirmed death from COVID-19 (i.e., MIR = 0) during the study time (1st wave).

**Cluster 1,** with 52 counties from 28 different states, had the highest MIR at the beginning of the study (intercept = 12.9% ± 3.1%) compare to other clusters (Table 4). This cluster continued having the highest MIR at the end of the study, on Jun 3, 2020 (Table S7, MIR = 13.2%). IA (Audubon, Floyd, and Guthrie counties), IL (Carroll, Clinton, and Jasper counties), NC (McDowell, Moore, Orange, and Polk counties), OK (Cotton, Le Flore, Mayes counties), and VA (Northumberland, Page, and Scott counties) were the most frequent states present in this cluster. Within this cluster, McHenry (ND), Crowley (CO), Terrell (GA), and Shelby (KY) counties had the highest COVID-19 MIR. COVID-19 MIR growth trajectory for the counties in this cluster showed a 5% decrease from Mar 25 (MIR = 12.9%) to April 1 (MIR = 7.9%) and stayed steady (flat) till April 8, 2020. From here, the rate slightly increased to MIR = 9% and stayed at this level till May 6, and thereafter, had another increase to MIR = 13.2% on Jun 3, 2020.

**Cluster 2** includes 74 counties from 27 different states. MI (Delta, Grand Traverse, Iosco, Lapeer, Oscoda, and Wexford counties), and WI (Adams, Bayfield, Buffalo, Clark, Door, Grant, and Marquette counties) were the most frequent states present in this cluster. Within this cluster, Winona (MN), Emmons (ND), and Lyon (KY) counties had the highest COVID-19 MIR. COVID-19 MIR growth trajectory for the counties in this cluster showed a 1.4% decrease from Mar 25 (MIR = 2.2%) to April 1 (MIR = 0.8%). From here, the rate slightly increased to MIR = 15.4% till May 6. From here till May 20, the rate slightly decreased to MIR = 13.7%), and again increased to MIR = 15.5% till May 27. Hereafter, the rate decreased to MIR = 9.1% till the end of the 1st wave (Jun 3, 2020).

**Cluster 3** includes 66 counties from 21 different states. IL (Bond, Boone, Ford, Jackson, and Tazewell counties), KY (Sumner, Grant, Laurel, Lincoln, McLean, Meade, and Pike counties), NC (Craven, Hertford, Jones, Rockingham, Wilkes, and Yadkin counties), TN (Carter Hamblen Hamilton, Macon, and Obion counties), and TX (Cherokee, Crosby, Grimes, Hale, Howard, Kleberg, Medina, and Wise counties) were the most frequent states present in this cluster. Within this cluster, Hamilton (TN), Benton (OR), Appanoose (IA), Crosby (TX), and Dickinson (MI) counties had the highest COVID-19 MIR. COVID-19 MIR growth trajectory for the counties in this cluster showed a 1.2% decrease from Mar 25 (MIR = 1.9%) to April 1 (MIR = 0.7%). From here, the rate increased to MIR = 22.3% on April 22 and then decreased to MIR = 5.6% by the end of the 1st wave on Jun 3, 2020.

**Cluster 4** includes 39 counties from 21 different states. MN (Brown, Itasca, and Kanabec counties), TX (Fisher, Harrison, Jackson, Lamar, Panola, Red River, Walker, and Wood counties), and VA (Brunswick, Campbell, and Northampton counties) were the most frequent states present in this cluster. Within this cluster, Beadle (SD), Panola (TX), Brown (MN), and Wyoming (PA) counties had the highest COVID-19 MIR. COVID-19 MIR growth trajectory for the counties in this cluster showed a 0.3% increase from Mar 25 (MIR = 0.8%) to April 1 (MIR = 1.1%) and stayed steady (flat) till May 13, 2020. From here, the rate sharply increased to MIR = 14.4% till May 27, and thereafter, slightly decreased to MIR = 10.5% till Jun 3, 2020.

**Cluster 5** includes 1406 counties from 45 different states. GA (including 117 counties), TX (including 85 counties), MS (including 69 counties), IN (including 63 counties), NC (including 62 counties), AL (including 54 counties), FL (including 53 counties), OH (including 51 counties), PA (including 50 counties), LA (including 49 counties), NY (including 49 counties), MI (including 46 counties), and IL (including 40 counties) were the most frequent states present in this cluster. Within this cluster, Pennington (SD), Dade (GA), Oglethorpe (GA), Marquette (MI), and Chaffee (CO) counties had the highest COVID-19 MIR. COVID-19 MIR growth trajectory for the counties in this cluster showed a slight increase from Mar 25 (MIR = 1.0%) to May 27 (MIR = 5.0%) and thereafter, had a slight decrease to MIR = 4.5% till the end of the 1st wave (Jun 3, 2020).

**Cluster 6** with 64 counties (from 28 different states) had the second-highest MIR at the beginning of the study (intercept = 9.8% ± 3.0%) compare to other clusters. However, on Jun 3, it had the third-lowest MIR compare to other clusters. GA (with seven counties), KY (with four counties), MI (with five counties), OH (with six counties), and VA (with six counties) are the most frequent states in this cluster. Iron (WI), Gallia (OH), Bourbon (KY), and Missaukee (MI) had the highest COVID-19 MIR trajectories within this cluster. COVID-19 MIR growth trajectory had a sharp increase from MIR = 9.8% on Mar 25 to MIR = 36.0% on April 1, 2020. Then. The rate had a sharp decrease to MIR = 9.5% till April 22 and continued decreasing with a gentle slope till Jun 3, 2020 (MIR = 7.7%).

**Cluster 7** includes 12 counties from 11 different states. TX (Lavaca and Barbour counties) was the most frequent state present in this cluster. Within this cluster, Catron (NM) county had the highest COVID-19 MIR. COVID-19 MIR growth trajectory for the counties in this cluster was MIR = 1.5% on Mar 25 and stayed steady (flat) till April 15. From here, the rate had a sharp increase to MIR = 26.2% till April 29, but thereafter, it had a sharp decrease to MIR = 14.5% till May 6, 2020. This rate then had a slight decrease to MIR = 11.6% till the end of the 1st wave (Jun 3, 2020).

**Cluster 8** includes 23 counties from 13 different states. OH (Highland, Perry, and Putnam counties), and TX (Comanche, Hansford, Hartley, and Martin counties) were the most frequent states present in this cluster. Within this cluster, Shasta (CA), Clare (MI), Jackson (KY), Mahnomen (MN), Carlisle (KY), Comanche (TX), and Martin (TX) counties had the highest COVID-19 MIR. COVID-19 MIR growth trajectory for the counties in this cluster was MIR = 1.9% on Mar 25 and stayed steady (flat) till April 29, 2020. From here, the rate had a sharp increase to MIR = 18.6% till May 6, but thereafter, it had a sharp decrease to MIR = 12.7% till May 20, 2020. From here, this rate had a slight decrease to MIR = 12.2% till the end of the 1st wave (Jun 3, 2020).

Details of the six clusters (including the cluster of counties with zero MIR) during the **2nd wave** are as follows: **Cluster 0** contains 156 counties with zero confirmed death from COVID-19 during the 2nd wave (i.e., MIR = 0). **Cluster 1,** with 32 counties from 7 different states (AR, GA, LA, MS, NM, SC, and TX), had the lowest MIR at the beginning of the 2nd wave (Intercept = 1.5% ± 0.3%). However, by the end of the 2nd wave (Sep 2, 2020), it had the second-highest MIR (MIR = 4.8%) compare to other clusters (with the maximum increase in COVID-19 MIR of 3.3%**↑**, Table S8). TX (Aransas, Atascosa, Bandera, Blanco, Burleson, Dickens, Duval, Fayette, Gillespie, Goliad, Grimes, Guadalupe, Hudspeth, Kenedy, Liberty, Marion, Medina, Newton, Sabine, San Jacinto, Stephens, Throckmorton, Upton, Wharton, and Zavala counties) was the most frequent state present in this cluster. Within this cluster, Blanco (TX), Sabine (TX), Marion (TX), and Throckmorton (TX) counties had the highest COVID-19 MIR. COVID-19 MIR growth trajectory for the counties in this cluster showed a 0.9% decrease from Jun 4 (MIR = 1.5%) to Jun 18 (MIR = 0.6%) and stayed steady (flat) till Jul 16, 2020. Hereafter, the rate sharply increased to MIR = 6.3% till Jul 30, it slightly decreased to MIR = 4.8% till Aug 27, 2020.

**Cluster 2** includes 1035 counties from 17 different states. TX (156 counties), GA (131 counties), NC (89 counties), and TN (87 counties) were the most frequent states present in this cluster. Within this cluster, Crosby (TX), Pearl River (MS), and Stonewall (TX) counties had the highest COVID-19 MIR. COVID-19 MIR growth trajectory for the counties in this cluster was MIR = 3.0% at the beginning of the 2nd wave (Jul 4) and steadily decreased to MIR = 1.8% till Aug 27, 2020.

**Cluster 3,** with 43 counties from 11 different states, had the second-highest MIR at the beginning of the 2nd wave (intercept = 10.6% ± 1.8%) compare to other clusters (Table 4). However, on Sep 2 (end of the 2nd wave), it had the highest MIR (MIR = 8.0%) compare to other clusters. TX (Briscoe, Coke, Culberson, Floyd, Hall, Lamb, Lynn, Oldham, Reagan, Red River, San Augustine, and Washington counties), and GA (Butts, Early, Hancock, Mitchell, Randolph, Sumter, Terrell, Turner, Upson, and Wilcox counties) were the most frequent states present in this cluster. Within this cluster, Catron (NM), Hall (TX), and Morton (KS) counties had the highest COVID-19 MIR. COVID-19 MIR growth trajectory for the counties in this cluster showed a 1.0% increase from Jun 4 (MIR = 10.6%) to Jul 2 (MIR = 11.6%). From here, the rate decreased to MIR = 8.3% till Jul 30 and stayed steady till Aug 27, 2020.

**Cluster 4** includes 59 counties from 15 different states. TX (25 counties), GA (Candler, Glascock, Hart, Laurens, Warren, and Wheeler counties), and KS (Cloud, Jewell, Nemaha, Stafford, Stanton, and Trego counties) were the most frequent states present in this cluster. Within this cluster, Matagorda (TX), Lee (TX), Lowndes (MS), Newton (AR), and Trego (KS) counties had the highest COVID-19 MIR. COVID-19 MIR growth trajectory for the counties in this cluster showed a 1.1% decrease from Jun 4 (MIR = 1.8%) to Jun 18 (MIR = 0.7%) and stayed steady (flat) till Jul 16, 2020. From here, the rate increased to MIR = 3.5% till Aug 13, and thereafter, slightly decreased to MIR = 3.3% till Aug 27, 2020.

**Cluster 5,** with 19 counties from 9 different states, had the highest MIR at the beginning of the study (intercept = 14.1% ± 4.5%) compare to other clusters (Table 4). However, on Aug 27, it had the third-lowest MIR compare to other clusters (Table S7, MIR = 4.0%). TX (Brown, Callahan, Fisher, Hood, Martin, and Palo Pinto counties) and OK (Cotton, Delaware, Kiowa, and Latimer counties were the most frequent states present in this cluster. Within this cluster, Fisher (TX), Cotton (OK), and Jenkins (GA) counties had the highest COVID-19 MIR. COVID-19 MIR growth trajectory for the counties in this cluster showed a 1.0% increase from Jun 4 (MIR = 14.1%) to Jun 18 (MIR = 15.1%) but thereafter, had a sharp decrease to MIR = 5.2% till Jul 16, 2020. This rate slightly decreased to MIR = 4.0% till Aug 27, 2020.

Details of the five clusters (including the cluster of counties with zero MIR) during the **3rd wave** are as follows: **Cluster 0** contains 111 counties with zero confirmed death from COVID-19 during the 3rd wave (i.e., MIR = 0). **Cluster 1,** with 125 counties from 11 different states, had the highest MIR at the beginning of the 3rd wave (intercept = 5.2% ± 0.2%). However, by the end of the 3rd wave (Nov 12, 2020), it had the second-highest MIR (MIR = 3.6%) compare to other clusters (also, with the maximum decrease in COVID-19 MIR of 1.6%↓, Table S9). IN (25 counties) and MI (25 counties) were the most frequent states present in this cluster. Within this cluster, Morton (KS) and Monroe (OH) counties had the highest COVID-19 MIR. COVID-19 MIR growth trajectory for the counties in this cluster showed a 1.6% decrease from Sep 3, 2020 (MIR = 5.2%) to Nov 12, 2020 (MIR = 3.6%).

**Cluster 2** with 47 counties from 12 different states had the lowest MIR at the beginning of the 3rd wave (intercept = 1.0% ± 0.6%) compare to other clusters (Tables 4 and S9). However, at the end of the 3rd wave (Nov 12, 2020), it had the third-highest MIR (MIR = 2.5%) with the highest increase in COVID-19 MIR over time. ND (Bottineau, Bowman, Divide, Emmons, McHenry, Morton Nelson, Renville, Sargent, and Sheridan counties), KS (Cheyenne, Decatur, Kingman, Lane, Lyon, Ness, Rooks, Russell, and Wilson counties), and IL (Clay, Edgar, Fayette, Greene, Hamilton, Marshall, and Wabash counties) were the most frequent states present in this cluster. Within this cluster, Jackson (SD), Bottineau (ND), and Ness (KS) counties had the highest COVID-19 MIR. COVID-19 MIR growth trajectory for the counties in this cluster was MIR = 1.0% at the beginning of the 3rd wave (Sep 3) and stayed steady (flat) till Sep 17, 2020. Hereafter, this rate had a sharp increase to MIR = 3.1% till Oct 15. From here, the rate decreased to MIR = 2.5% till Nov 12, 2020.

**Cluster 3,** with 11 counties from 6 different states (IL, KS, MO, NE, ND, OH), had the second-highest MIR at the beginning of the 3rd wave (intercept = 3.5% ± 1.1%) compare to other clusters (Table 4). However, on Nov 12 (end of the 3rd wave), it had the highest MIR (MIR = 3.8%) compare to other clusters. NE (Cherry, Dundy, and Perkins counties) was the most frequent state present in this cluster. Within this cluster, and Perkins (NE) counties had the highest COVID-19 MIR. COVID-19 MIR growth trajectory for the counties in this cluster showed a sharp increase (3.9%↑) from MIR = 3.5% on Sep 3 to MIR = 7.4% on Sep 17. From here, the rate decreased to MIR = 2.5% till Nov 12, 2020.

**Cluster 4** includes 761 counties from 12 different states. MO (99 counties), IA (83 counties), IL (75 counties), and MN (74 counties) were the most frequent states present in this cluster. Within this cluster, Phillips (KS) and Renville (MN) counties had the highest COVID-19 MIR. COVID-19 MIR growth trajectory for the counties in this cluster was MIR = 1.4% at the beginning of the 3rd wave and decreased to MIR = 1.2% till the end of the wave (on Nov 12, 2020).

More information about the COVID-19 MIR estimation at both the beginning and the end of each wave, the amount of increase (or decrease) in this rate, and each cluster's rank are presented in tables S7–S9. One important point in Table S7 is that during the **1st wave**, counties in cluster 4 (MIR: 0.8% → 10.5%) and cluster 7 (MIR: 1.5% → 11.6%) had the highest increase in COVID-19 MIR from Mar 25 to Jun 3, 2020. During the **2nd wave**, counties in cluster 1 (MIR: 1.5% → 4.8%) had the highest increase in COVID-19 MIR; whereas, counties in cluster 5 (MIR: 14.1% → 4.0%) had the highest decrease in this rate from Mar 25 to Jun 3, 2020 (Table S8). During the **3rd wave**, counties in cluster 2 (MIR: 1.0% → 2.5%) had the highest increase in this rate from Sep 3 to Nov 12, 2020 (Table S9). Counties in cluster 1 (MIR: 5.2% → 3.6%) had the highest decrease in COVID-19 MIR; however, it had the second-highest COVID-19 MIR compare to other clusters.

Tables 5, 6 and 7 show the significant risk factors in each cluster during the 1st, 2nd, and 3rd waves, respectively. To find the odds ratios (ORs), we used cluster 0 as the baseline (with MIR = 0) and compared all other clusters to it. The full results of the multinomial logit models are provided in the Supplement (Tables S10–S12).

**Table 5:** 1st Wave (Mar 25–Jun 3, 2020): significant risk factors and their odds ratios in each cluster compare to cluster 0 (counties with MIR = 0). Blank spots indicate the insignificant risk factors.

Variable	Cluster							
	1	2	3	4	5	6	7	8
**Comorbidities and disorders**								
CVD					1.1*		0.7	
Cardiomyopathy and myocarditis								
Hypertensive heart disease							1.4	
Peripheral vascular disease					0.5			
Atrial fibrillation					0.8			
Cerebrovascular disease					0.9		1.4	
Diabetes				1.1				
Hepatitis	0.3	2.1	0.5	4.8	10.7	0.1	1.1	1.6
HIV/AIDS								
TB	0.7		2.3	0.6		1.3	1.5	
Lower respiratory infection	0.9							
Interstitial lung disease and pulmonary sarcoidosis								
Ischemia					0.9		1.3	
Mesothelioma	0.2	2.1		3.3			0.8	3.7
Tracheal cancer								
Pancreatic cancer		1.5					1.7	
Drug use disorder			1.1			1.1	1.1	
Alcohol use disorder			0.8					
**Demographics and social**								
Female-AA%		2.0	2.1	1.7	17.9	3.0	0.9	
Female-WA%	0.2	0.1	0.1	0.3	0.1	0.1	0.5	0.4
Male-AA%		2.5	1.6		5.0	3.0	0.8	0.8
Male-WA%	0.2	0.1	0.1	0.3	0.4	0.1	0.5	
Asian%		0.5			1.3			
Smokers%								
Unemployed%								
Income rate							0.3	
Uninsured%		1.1			1.1			
Food insecurity				1.1	0.9			
Fair/poor health		0.8			0.9	0.8		
**Environmental**								
Population density	1.01	1.02	1.01		1.02	1.01		
AQI					1.7		0.8	
Temperature				0.9				
PM					0.1		1.6	0.7

*For instance, OR 1.1 means that 1% increase in CVD MR is associated with a 10% increase in the relative log odds of being in cluster 5 vs. cluster 0 (MIR = 0).

**Table 6:** 2nd Wave (Jun 4–Sep 2, 2020): significant risk factors and their odds ratios in each cluster compare to cluster 0 (counties with MIR = 0). Blank spots indicate the insignificant risk factors.

Variable	Cluster				
	1	2	3	4	5
**Comorbidities and disorders**					
CVD					
Cardiomyopathy and myocarditis					
Hypertensive heart disease					
Peripheral vascular disease					
Atrial fibrillation					
Cerebrovascular					
Diabetes		1.1*			
Hepatitis	13.1	53.1		0.2	13.9
HIV/AIDS	2.3		2.9		2.2
Tuberculosis	2.1	44.5	0.2		1.8
Asthma		0.3	0.1		
Lower respiratory infection					
Ischemia					
Mesothelioma					
Tracheal cancer					
Pancreatic cancer					
Rheumatic disease			2.5		
**Demographics and social**					
Age	0.9	0.8		0.9	0.8
Female-AA%		101.1	0.4	0.2	0.1
Female-WA%		0.1			
Male-AA%			2.9	0.2	0.2
Male-WA%		6.3	0.3		
Asian%					
Smokers%					
Unemployed%	1.5	1.3		1.6	1.5
Income Rate					
Uninsured%					
Food insecurity		0.9			
Fair/poor health					
**Environmental**					
Population density		1.01	1.01	1.01	1.01
AQI			0.4		
Temperature	1.2	1.1	1.1	1.1	
PM			11.7		

*For instance, OR 1.1 means that 1% increase in diabetes MR is associated with a 10% increase in the relative log odds of being in cluster 2 vs. cluster 0 (MIR = 0).

**Table 7:** 3rd Wave (Sep 3–Nov 12, 2020): significant risk factors and their odds ratios in each cluster compare to cluster 0 (counties with MIR = 0). Blank spots indicate the insignificant risk factors.

Variable	Cluster			
	1	2	3	4
**Comorbidities and disorders**				
CVD				
Cardiomyopathy and myocarditis	1.3			1.2
Hypertensive heart disease				
Peripheral vascular disease				
Cerebrovascular				
Diabetes	1.2*			
Hepatitis	0.5	0.1	0.6	10,099.2**
HIV/AIDS		4.0	78.8	
Tuberculosis	3.2	33.6	7.0	31.4
Lower respiratory infection		1.1	1.1	
Interstitial lung disease and pulmonary sarcoidosis		0.5		
Asthma			0.4	
COPD				
Ischemia				
Mesothelioma	9.3	20.5	3.6	8.7
Tracheal cancer	0.9			
Leukemia				
Pancreatic cancer				
Drug use disorder		0.7		
Alcohol use disorder	0.6			
**Demographics and social**				
Female-AA%	33.4	0.1	0.4	22.9
Female-WA%	0.03	0.02	0.1	0.001
Male-AA%	0.1	0.1	0.1	1.3
Smokers%	1.3			
Unemployed%				
Income Rate				
Uninsured%				
Food insecurity	0.8			
Fair/poor health				
**Environmental**				
Population density	1.02			1.02
AQI		0.7		1.4
Temperature			0.8	
PM			0.6	0.3

*For instance, OR 1.2 means that 1% increase in diabetes MR is associated with a 20% increase in the relative log odds of being in cluster 1 vs. cluster 0 (MIR = 0).

**Due to the sparsity of hepatitis mortality rate in these particular counties (during the 3rd wave), the odds ratio estimation of hepatitis is not reliable. One way around this issue is to categorize the hepatitis MR and use the categorical version of this variable in the multinomial model. However, we decided to avoide this approach to stay consistent with the rest of the results.

For the **1st wave**, hypertensive heart disease (OR 1.4), cerebrovascular disease (OR 1.4), hepatitis (OR 1.1), TB (OR 1.5), ischemia (OR 1.3), pancreatic cancer (OR 1.7), drug use disorder (OR 1.1), and PM (OR 1.6) are significantly associated exhibiting a 40%, 40%, 10%, 50%, 30%, 70%, 10%, and 60% increase in the relative log-odds of being in **“vulnerable cluster 7” vs. cluster 0**, respectively (Tables 5 and S10). Population density (OR 1.01) is significantly associated with a 1% increase in the relative log-odds of being in **cluster 1 vs. cluster 0**. Hepatitis (OR 2.1), mesothelioma (OR 2.1), pancreatic cancer (OR 1.5), female AA% (OR 2.0), male-AA% (OR 2.5), uninsured% (OR 1.1), and population density (OR 1.02) are significantly associated with 110%, 110%, 50%, 100%, 150%, 10%, and 2% increase in the relative log-odds of being in **cluster 2 vs. cluster 0**, respectively. TB (OR 2.3), drug use disorder (OR 1.1), female AA% (OR 2.1), male AA% (OR 1.6), and population density (OR 1.01) are significantly associated with 130%, 10%, 40%, 110%, 60%, and 1% increase in the relative log-odds of being in **cluster 3 vs. cluster 0**, respectively. Diabetes (OR 1.1), hepatitis (OR 4.8), mesothelioma (OR 3.3), female-AA% (OR 1.7), and food insecurity (OR 1.1) are significantly associated with 10%, 380%, 230%, 70%, and 10% increase in the relative log-odds of being in **cluster 4 vs. cluster 0**, respectively. CVD (OR 1.1), hepatitis (OR 10.7), female-AA% (OR 17.9), male-AA% (OR 5.0), Asian% (OR 1.3), uninsured% (OR 1.1), population density (OR 1.02), and AQI (OR 1.7) are significantly associated with an increase in the relative log-odds of being in **cluster 5 vs. cluster 0**. Drug use disorder (OR 1.1), female AA% (OR 3.0), male AA% (OR 3.0), and population density (OR 0.01) are significantly associated with 10%, 200%, 200%, and 2% increase in the relative log-odds of being in **cluster 6 vs. cluster 0**, respectively. Hepatitis (OR 1.6), and mesothelioma (OR 3.7) are significantly associated with 60% and 270% increase in the relative log-odds of being in **cluster 8 vs. cluster 0**. Table S10 contains the detailed output of the multinomial logit model for all potential risk factors in each cluster separately.

For the **2nd wave**, hepatitis (OR 13.1), HIV/AIDS (OR 2.3), TB (OR 2.1), unemployed% (OR 1.5), and temperature (OR 1.2) are significantly associated with a 1210%, 130%, 110%, 50%, and 20% increase in the relative log-odds of being in **“vulnerable cluster 1” vs. cluster 0**, respectively (Tables 6 and S11). Diabetes (OR 1.1), hepatitis (OR 53.1), TB (OR 44.5), female AA% (OR 101.1), male WA% (OR 6.3), unemployed% (OR 1.1), population density (OR 1.01), and temperature (OR 1.1) are significantly associated with an increase in the relative log-odds of being in **cluster 2 vs. cluster 0**. HIV/AIDS (OR 2.9), rheumatic disease (OR 2.5), male AA% (OR 3.0), population density (OR 1.01), temperature (OR 1.1), and PM (11.7) are significantly associated with a 190%, 150%, 200%, 1%, 10%, and 1070% increase in the relative log-odds of being in **cluster 3 vs. cluster 0**, respectively. Unemployed% (OR 1.6), population density (OR 1.01), and temperature (OR 1.1) are significantly associated with a 60%, 1%, and 10% increase in the relative log-odds of being in **cluster 4 vs. cluster 0**. Hepatitis (OR 13.9), HIV/AIDS (OR 2.2), TB (OR 1.8), unemployed% (OR 1.5), and population density (OR 1.01) are significantly associated with a 1290%, 120%, 80%, 50%, and 1% increase in the relative log-odds of being in **cluster 5 vs. cluster 0**.

For the **3rd wave**, cardiomyopathy and myocarditis (OR 1.3), diabetes (OR 1.2), TB (OR 3.2), mesothelioma (OR 9.3), female AA% (OR 33.4), smokers% (OR 1.3), and population density (OR 1.02) are significantly associated exhibiting an increase in the relative log-odds of being in **“vulnerable cluster 1” vs. cluster 0** (Tables 7 and S12). HIV/AIDS (OR 4.0), TB (OR 33.6), Lower respiratory infection (OR 1.1), and mesothelioma (OR 20.5) are significantly associated with an increased relative log-odds of being in **cluster 2 vs. cluster 0**. HIV/AIDS (OR 78.8), TB (OR 7.0), Lower respiratory infection (OR 1.1), and mesothelioma (OR 3.6) are significantly associated with an increased relative log-odds of being in **cluster 3 vs. cluster 0**. Cardiomyopathy and myocarditis (OR 1.2), TB (OR 31.4), mesothelioma (OR 8.7), female AA% (OR 22.9), male AA% (OR 1.3), population density (OR 1.02), and AQI (OR 1.4) are significantly associated with an increase in the relative log-odds of being in **cluster 4 vs. cluster 0.**

**Figure 3:**
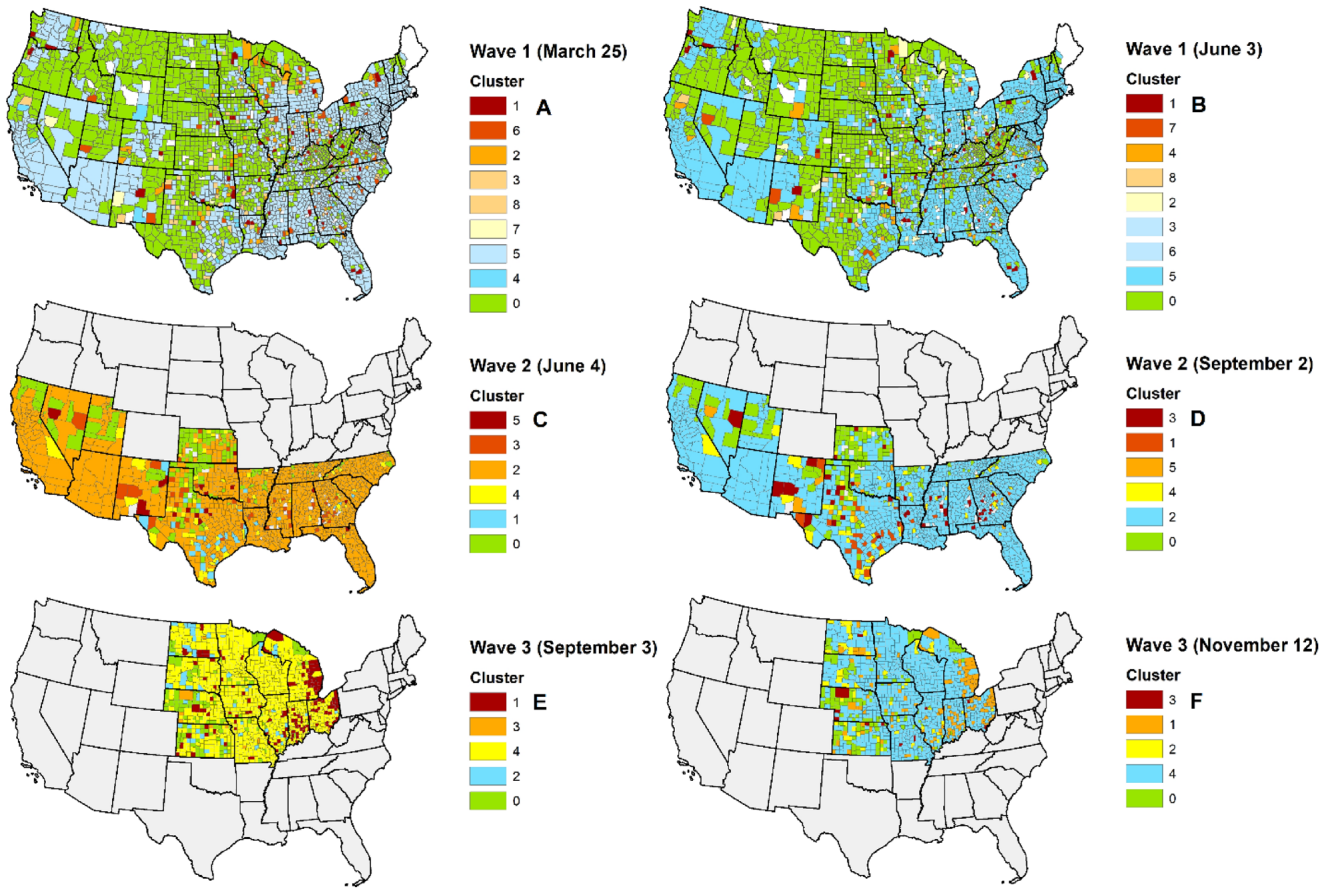
Geographical distribution of the clusters of U.S. counties based on the estimated COVID-19 MIR growth trajectories at the: (**A)** beginning of the1st wave (Mar 25, 2020), (**B)** end of the 1st wave (Jun 3, 2020), (**C)** beginning of the 2nd wave (Jun 4, 2020), (**D)** end of the 2nd wave (Sep 2, 2020), (**E)** beginning of the 3rd wave (Sep 3, 2020), and (**F)** end of the 3rd wave (Nov 12, 2020). Red color indicates the highest MIR, blue color indicates the lowest MIR, and green color shows the counties with MIR = 0.

Figure 3 shows the geographical distribution of the clusters of the contiguous United States during the 1st (Mar 25–Jun 3, 2020), 2nd (Jun 4–Sep 2, 2020), and 3rd (Sep 3–Nov 12, 2020) waves, based on the estimated COVID-19 MIR growth trajectory over time.

## Discussion

This study investigated the county-level COVID-19 confirmed cases and death from Mar 25 to Nov 12, 2020, in a longitudinal fashion in the contiguous United States. We considered Mar 25 to Jun 3 as the “1st wave”, Jun 4 to Sep 2 as the “2nd wave”, and Sep 3 to Nov 12 as the “3rd wave” of COVID-19. We assessed the growth trajectories of COVID-19 MIR and found the county-level clusters of the contiguous United States with similarities in COVID-19 MIR growth trajectory over time. We also considered the effects of different county-level potential risk factors on MIR (for each wave), including comorbidities & disorders, demographics & social factors, and environmental factors. We selected MIR as a measure of mortality since it also considers the number of confirmed cases to adjust the mortality rates. However, the estimates of all COVID-19 epidemiological-measures (i.e., incidence, prevalence, and mortality rates) are subject to bias due to the imprecise number of affected (confirmed) cases, especially those with mild or no disease symptoms. Moreover, there are not enough studies presenting and discussing county level risk factors, especially pre-existing comorbidities, with COVID-19 incidence and mortality.

We found nine, six and five clusters of U.S. counties (including a cluster of counties with zero MIR) based on the COVID-19 MIR pattern (growth trajectory) using a longitudinal LGMM in the 1st, 2nd and 3rd waves, respectively. All counties in the same cluster have a similar COVID-19 MIR growth pattern over the study time. This approach considered both spatial and temporal heterogeneities in COVID-19 MIR due to pre-existing comorbidities, environmental factors, and demographics. We also identified significant risk factors associated with the identified clusters using a multinomial logit model. It is shown that different age and sex distributions in the U.S. counties impact differentially COVID-19 mortality and severity^72,73^. Race is also a factor that leads to heterogeneity. For instance, it has been reported African Americans having a higher risk of getting infected, experiencing more severe COVID-19 and death^74^. Further, note that about 43% of the northern and central U.S. counties did not experience death from COVID-19 until Jun 3.

**During the 1st wave,** nearly 116 counties in clusters 1 and 6 had the highest mean COVID-19 MIR at the beginning of the study on Mar 25, 2020. On Jun 3, 2020, cluster 1 still had the highest mean COVID-19 MIR (MIR = 13.2%), while counties in cluster 6 improved to the third lowest (excluding the cluster with MIR = 0). Counties in cluster 7 had a low level of COVID-19 MIR at the beginning of the study on Mar 25 (MIR = 1.5%). However, they had a very dramatic increase (10.1%↑) in COVID-19 MIR till Jun 3, 2020 (MIR = 11.6%). Cluster 7 became the one with the second-highest COVID-19 MIR at the end of the 1st wave on Jun 3, 2020. Based on these clustering results, we considered clusters 1 and 7 as the so-called **“more vulnerable”** clusters of counties requiring a more concerted effort and stronger mitigation strategies to control disease mortality. Cluster 7 includes the following counties: Marion (KS), Seward (NE), Churchil (NV), Catron (MN), Crater (OK), Benton (TN), Gonzales (TX), Lavaca (TX), and Barbour (WV). Most frequent states in cluster 1 were IA (Audubon, Floyd, and Guthrie counties), IL (Carroll, Clinton, and Jasper counties), NC (McDowell, Moore, Orange, and Polk counties), OK (Cotton, Le Flore, Mayes counties), and VA (Northumberland, Page, and Scott counties). In states where the majority of “more vulnerable” clusters (1 and 7) were during the first wave, there were no state-wide face-mask mandates, which might cause an increase in COVID-19 incidence and subsequently in COVID-19 MIR^75,76^. For instance, OK does not have any state mandate for public mask-wearing to date. A partial mask-wearing rule was announced in IA in Nov 2020 (for Iowans age 2 and up in indoor public places). Therefore, different face-mask mandates can be one reason for having higher COVID-19 MIR in these vulnerable clusters and be further mitigated by issuing state-wide full face-covering mandates.

TB (OR 1.3) and drug use disorder (OR 1.1) are two significant comorbidities associated with a 30% and 10% increase in the odds of being in cluster 7 vs. cluster 0. Among the demographic and environmental factors, male-WA% (OR 1.8) and PM (OR 1.1) are significantly associated with an 80% and 10% increase in the relative log-odds of being in cluster 7 vs. cluster 0. Therefore, protecting subjects with TB and drug use disorder and managing the ${\mathrm{PM}}_{2.5}$ level of the air (a mixture of solid particles and liquid droplets found in the air, such as dust, dirt, or smoke) can help ameliorate the COVID-19 mortality in these counties. Moreover, more than 80% of the counties in clusters 1 and 7 were rural areas based on the U.S. Census Bureau definition (https://www.census.gov/programs-surveys/geography/guidance/geo-areas/urban-rural.html). Lack of access to health and critical care infrastructure and more limited resources, in general, may be responsible for higher COVID-19 MIR. Therefore, addressing these factors would be beneficial in the long run for managing the epidemic.

**During the 2nd wave**, nearly 62 counties in clusters 3 (MIR = 10.6%) and 5 (MIR = 14.1%) had the highest mean COVID-19 MIR at the beginning of the wave on Jun 4, 2020. However, on Sep 2, 2020, cluster 3 had the highest mean COVID-19 MIR (MIR = 4.8%), while counties in cluster 5 improved to the third lowest (MIR = 4.0%). Counties in cluster 1 had the lowest level of COVID-19 MIR at the beginning of the 2nd wave on Jun 4 (MIR = 1.5%), but experienced a dramatic increase (3.3%↑) in COVID-19 MIR till Sep 2, 2020 (MIR = 4.8%), and therefore became the highest COVID-19 MIR at the end of the 2nd wave. Based on the clustering result (as of Sep 2, 2020), we considered cluster 1 as the so-called **“more vulnerable”** cluster of counties requiring more attention to control disease mortality. TX (Aransas, Atascosa, Bandera, Blanco, Burleson, Dickens, Duval, Fayette, Gillespie, Goliad, Grimes, Guadalupe, Hudspeth, Kenedy, Liberty, Marion, Medina, Newton, Sabine, San Jacinto, Stephens, Throckmorton, Upton, Wharton, and Zavala counties) was the most frequent state present in this cluster. Cluster 1 includes the following counties: Marion (KS), Seward (NE), Churchil (NV), Catron (MN), Crater (OK), Benton (TN), Gonzales (TX), Lavaca (TX), and Barbour (WV). Moreover, Blanco (TX), Sabine (TX), Marion (TX), and Throckmorton (TX) counties had the highest COVID-19 MIR. Only in mid-July (middle of the 2nd wave), the TX governor signed an executive order requiring residents (> 10 yo) to wear a face mask in public (state-wide), yet nearly 80 counties have opted out of the order, and others are not enforcing it. Therefore, the difference between face-mask mandates can also be one reason for having higher COVID-19 MIR in cluster 1.

Hepatitis (OR 13.1), HIV/AIDS (OR 2.3), and TB (OR 2.1) are three significant comorbidities that are associated with an increase in the odds of being in cluster 1 vs. cluster 0. Among the demographic and environmental factors, unemployed% (OR 1.5) and temperature (OR 1.2) are significantly associated with a 50% and 20% increase in the relative log-odds of being in cluster 1 vs. cluster 0 (tables S10–S12). Therefore, protecting subjects with hepatitis, HIV/AIDS, and TB and managing the unemployment rate can help ameliorate the COVID-19 mortality in these counties. The effect of temperature, however, could be due to other confounding variables. For instance, when the weather is cold, people spend more time together indoors. Therefore, informing the residents of these counties about distancing and mask-wearing may help to improve the COVID-19 MIR. Moreover, about 60% of the counties in cluster 1 were rural areas based on the U.S. Census Bureau definition (https://www.census.gov). Similar to the conlucsion for the 1st wave, lack of access to health and critical care infrastructure and more limited resources, in general, may be responsible for higher COVID-19 MIR.

**During the 3rd wave**, 125 counties in cluster 1 (MIR = 5.2%) had the highest mean COVID-19 MIR at the beginning of the wave on Jun 4, 2020. Although the mean COVID-19 MIR of the counties in cluster 1 decreased (MIR = 3.6%) by the end of the wave, this cluster remained the second-highest compared to other clusters. Based on the clustering result (as of Nov 12, 2020), we considered cluster 1 as the so-called **“more vulnerable”** cluster of counties requiring more attention to control disease mortality. IN (Bartholomew, Boone, Carroll, Daviess, Dearborn, Decatur, Floyd, Franklin, Greene, Hancock, Hendricks, Howard, Jennings, Johnson, Lawrence, Madison, Montgomery, Morgan, Newton, Ohio, Orange, Perry, Pike, Pulaski, and Tipton counties) and MI (Alcona, Alpena, Arenac, Bay, Clare, Crawford, Genesee, Gogebic, Gratiot, Hillsdale, Iosco, Jackson, Keweenaw, Lapeer, Macomb, Muskegon, Oakland, Ogemaw, Otsego, Saginaw, St. Clair, Sanilac, Shiawassee, Tuscola, and Wayne counties) were two most frequent states present in this cluster. Moreover, Morton (KS) and Monroe (OH) counties had the highest COVID-19 MIR. Regarding the face-covering rules in the two most frequent states represented by the counties in cluster 1, in MI mask-wearing order was issued only in Oct 2020 (for people age 5 and up, in most public places). The IN governor ordered mask-wearing (for Hoosiers age 8 and up, in indoor and outdoor public spaces) only at the beginning of Aug (middle of the 2nd wave). Therefore, having inadequate/no rules for face covering in these states can cause a worse COVID-19 MIR trend.

Cardiomyopathy and myocarditis (OR 1.3), diabetes (OR 1.2), TB (OR 3.2), mesothelioma (OR 9.3) are four significant comorbidities that are associated with an increase in the odds of being in cluster 1 vs. cluster 0. Among the demographic and environmental factors, female AA% (OR 33.4), smokers% (OR 1.3), and population density (OR 1.02) are significantly associated with increased relative log-odds of being in cluster 1 vs. cluster 0 (tables S10–S12). Therefore, protecting subjects with diabetes, TB, mesothelioma and cardiomyopathy and myocarditis, and smoking history can help ameliorate COVID-19 mortality in these counties. The effect of population density, however, could be complicated and due to other confounding variables. At the beginning of the COVID-19 pandemic, dense (urban) areas around the world such as New York (USA), Madrid (Spain), Milan (Italy), London (UK), and Tehran (Iran) were identified as disease hotspots. In our analysis, nearly 40% of the counties in cluster 1 (during the 3rd wave) were urban areas based on the U.S. Census Bureau definition (https://www.census.gov). One reason that may explain the effect of population density on disease mortality/spread could be that large cities are mostly connected with many other locations^77^. Crowding and transport infrastructure quality are conducive for the spread of the disease^78^. Therefore, addressing these factors and continuously informing residents about social distancing, mask-wearing, and self-isolation (and household quarantine) would be beneficial in the long run for managing the epidemic in this region.

Amongst the comorbidities, we found a significant positive association between COVID-19 MIR and heart diseases, including cardiomyopathy and myocarditis (0.15% MIR↑ in the 1st wave, and 0.12% MIR↑ in the 2nd wave), hypertensive heart disease (0.11% MIR↑ in the 1st wave, and 0.09% MIR↑ in the 2nd wave), peripheral vascular disease (0.31% MIR↑ in the 1st wave), and cerebrovascular disease (0.07% MIR↑ in the 1st wave, and 0.07% MIR↑ in the 2nd wave). This finding is in accordance with recent studies on the topic, even though its etiology remains uncertain. This can be due to antiviral drugs (as a treatment of COVID-19), which can cause different cardiovascular disorders (such as cardiac insufficiency and arrhythmia)^79^. Moreover, most of the patients with pre-existing heart disorders use renin–angiotensin–aldosterone system (RAAS) blockers, which are suggested to increase the COVID-19 severity and MR^80,81^. Additionally, SARS-CoV-2 infection can act as a precipitating factor that worsens the cardiac insufficiency and leads to death in patients with pre-existing heart complications^79^. Cardiovascular diseases can also increase the COVID-19 severity and MR via aggravating pneumonia^79^. Historically, it is shown that patients with pre-existing heart and lung diseases had a higher mortality rate from SARS^18,25–30^. The same findings have been reported in China^16,17,82^ and the United Kingdom^83^. Lippi et al. showed that about 20% of hospitalized COVID-19 cases had heart complications, as well^17^. A meta-analysis with 46,248 confirmed COVID-19 cases showed that patients with severe disease were more likely to have CVD (odds ratio = 3.4) and hypertensive heart disease (odds ratio = 2.4)^84^. Recent studies have reported ACE2 as the coreceptor for the coronavirus in patients with different complications as well as heart and lung disorders compared with healthy individuals^30,85^. There is also evidence showing the critical role of the ACE2 and its peptides in the inflammatory^86,87^ and oxidative organ activities^88,89^, which are significant triggers in the initiation and progression of cardiovascular disease, cardiac hypertrophy, lung complications, and acute pancreatitis.

We did not find a significant positive association between most of the respiratory diseases (including COPD, Asthma, and lower respiratory infection) and COVID-19 MIR, which is consistent with the Halpin et al. study^4^, Onder et al. in Italy (Mar 2020)^5^, and the *CDC* report of health conditions’ prevalence in the USA (April 2020)^6^. We only found a positive association between interstitial lung disease and pulmonary sarcoidosis during the 3rd wave. One possible explanation might be that having CLD causes a different immune response, which eventually protects against infection from SARS-CoV2^4^. However, this is not supported by other publications showing a significant association between COPD and an increased COVID-19 MR. Another possibility is that treatments and therapies used by patients with CLD can protect against COVID-19 as well (for instance, topical intra-nasal sprays^90^ and mPGES-1^91,92^), or that CLD treatments can reduce symptoms and hence affecting COVID-19 diagnosis^4^. Notably, the *Chinese CDC* (http://www.chinacdc.cn/en/) has reported a 6.3% COVID-19 case-fatality rate for cases with pre-existing chronic respiratory diseases.

Besides heart diseases, we found significant positive associations between COVID-19 MIR and cancer, including mesothelioma (0.58% MIR↑ in the 1st wave) and pancreatic (0.51% MIR↑ in the 1st wave) in the United States. Typically, patients with cancer are known to be at higher risk for community respiratory viruses (such as influenza and coronaviruses) due to their suppressed immune system and poor physiological baseline^93–95^. Based on a descriptive study from Wuhan, China (Mar 2020), the incidence of COVID-19 patients with pre-existing cancer was about 1%, which is five times higher than the general cancer incidence in China^64^. In a report of 72 314 cases from the *Chinese CDC* (Mar 2020), the COVID-19 case fatality for cancer patients was 3.5% higher than those without cancer^96^. In another report from Italy (April 2020), the prevalence of pre-existing cancer among COVID-19 death was 16.5%^5^. Du et al., in a multi-omics study, indicated an indirect connection between the ACE2 pathway and cancer via Transforming Growth Factor Beta 1, *TGFB1,* association with colorectal cancer^97,98^.

Our findings also indicated that demographics and social factors at the county level, such as mean age, drug use disorders, smokers%, uninsured%, and population density, significantly increased COVID-19 MIR by 0.12%, 0.08%, 0.11%, 0.08%, and 0.0003%, respectively. One possible explanation might be that uninsured patients or patients with drug use disorder, especially in the areas with more health disparities, are less likely to seek medical care^99,100^. Moreover, drug use disorders can result in increased inflammation of multiple organ systems, particularly lungs, which may lead to respiratory failure. In turn, it can directly contribute to the elevated mortality rate of COVID-19 among confirmed cases. Marsden et al. showed that people with opioid use disorder have a higher prevalence of co-occurrence of health problems, subsequently leading to an increased rate of COVID-19^101^. Regarding the effect of population density on disease mortality/spread, one reason could be that the large cities are mostly connected with many other locations^77^; plus, crowding is conducive to the spread of the disease.

This study has several limitations. **First**, the mortality and MIR estimates from the current COVID-19 related data are biased since most of the individuals with mild or no symptoms have not been tested for COVID-19 in most of the counties. Moreover, the COVID-19 reporting system appears to differ regionally, which introduces further inaccuracies in the available data. For example, for a small number of counties, we found MIR = 100%, which is an unlikely event and can be due to an incomplete disease recording system. Timely sharing of information and collaboration between organizations and governors can partly solve this problem. There also needs to be additional testing and follow-ups to have higher quality data, especially for younger individuals with mild symptoms. Recent data (CDC Jun 19, 2020^102^) showed that more young people are testing positive for COVID-19 in the United States. **Second**, the reporting of disease data is mostly based on ICD9/10 codes, which can be fairly inaccurate^103^. **Third**, the analysis was based on county-level data. It would be beneficial to analyze individual-level and multi-countries data to gain deeper insights into the impact of risk factors on COVID-19 progression. **Fourth**, some of the counties, especially in Maine, were excluded from the study because some of the environmental factors such as climate and air pollution were not directly available. **Fifth**, different testing strategies (especially among health-care workers), re-opening, self-isolation, physical distancing, and mask policies can act as cofounders in the analysis of COVID-19 MIR.

**In summary**, accounting for heterogeneity in both risk factors and COVID-19 mortality patterns over time leads to a more informative clustering system, which can then be leveraged in managing the epidemic by identifying and informing groups of people at higher risk and also in managing healthcare resources (access to facilities, ICUs, vaccination, etc.) more judiciously. Findings of this study suggest that counties in clusters 1 and 7 (in the 1st wave), cluster 1 (in both 2nd and 3rd waves) experience higher COVID-19 MIR growth trajectories over time and are facing more challenges due to the prevalence of rural counties (60–80%), and different face-covering rules/mandates in managing the disease. Further, heart complications and cancer were statistically significant pre-existing comorbidities related to COVID-19 MIR across the U.S. TB, drug use disorder, HIV/AIDS, diabetes, and hepatitis were explicitly associated with an increased chance of being in a more “vulnerable” cluster.

## Supplementary Information

Supplementary Information

## Data Availability

All datasets used in the current study are publicly available (sources are mentioned in Table S1). Datasets generated during the study are available from the corresponding author.
