# Supplementary material for: County-level longitudinal clustering of COVID-19 mortality to incidence ratio in the United States
Source: Sci Rep. 2021 Feb 4;11:3088. doi: 10.1038/s41598-021-82384-0 (PMC7862666; doi:10.1038/s41598-021-82384-0)
Supplement: Supplementary file 1 — Supplementary Information [file 41598_2021_82384_MOESM1_ESM.docx]

**Supplementary Materials**

**County-Level Longitudinal Clustering of COVID-19 Mortality to Incidence Ratio in the United States**

Nasim Vahabi^1*^, Masoud Salehi^2*^, Julio D. Duarte^3^, Abolfazl Mollalo^4^, George Michailidis^1**^

1. Informatics Institute, University of Florida, Gainesville, FL, USA
2. Department of Biostatistics, College of Public Health, Iran University of Medical Sciences, Tehran, Iran
3. Center for Pharmacogenomics, Department of Pharmacotherapy and Translational Research, College of Pharmacy, University of Florida, Gainesville, FL, USA
4. Department of Public Health and Prevention Sciences, School of Health Sciences, Baldwin Wallace University, Berea, OH, USA

*: Co-first author

**: Correspondence author

[gmichail@ufl.edu](mailto:gmichail@ufl.edu)

**SUPPLEMENTARY LITERATURE REVIEW**

**Comorbidities:**

**Chronic lung diseases, CLD:** COVID-19 is an acute respiratory disease that primarily affects the pulmonary alveolar epithelial cells, which can lead to respiratory failure and death^[1]^. There are different hypotheses about whether people with pre-existing CLD (especially chronic obstructive pulmonary disease, COPD) would be at higher risk of infection with the SARS-CoV-2 virus representing more severe symptoms than others.

Halpin et al. showed that the CLD prevalence among COVID-19 cases was less than the estimated prevalence in the general population^[2]^. In a study from Italy (March 23, 2020), COPD was not reported for any of the patients who died from COVID-19 (n=355, mean-age=79.5)^[3]^. Similarly, in data in the USA (March 31, 2020), chronic respiratory diseases were comorbidities in 8.5% of patients with COVID-19, compared to the GBD estimate of 11.3% for the same disease^[4]^. Halpin et al. have discussed three of the possible factors leading to the lower prevalence of COPD (and Asthma) in COVID-19 patients^[2]^.

There are also a number of published studies showing the synergistic effect of CLD in worsening the severity of COVID-19. Guan et al. reported more than 50% of chronic pulmonary disease for COVID-19 patients who were also admitted to ICU^[5]^. In a meta-analysis study on both Chinese- and English-language published articles, Zhao et al. showed that pre-existing COPD was significantly associated with a nearly 4-fold higher risk of developing severe COVID-19. The association remained significant in the subgroup of patients with death or ICU-required patients^[6]^. Moreover, in large case-series, they reported a higher prevalence of COPD in patients with severe presentation and worse outcomes^[7]^. In another meta-analysis (May 1, 2020), the reported prevalence of COPD patients was 2% in COVID-19 cases. They showed that although the COPD prevalence was low, it was significantly associated with a higher risk of more severe COVID-19 (63%), and higher mortality (60%)^[8]^. Brake et al. reported higher (upregulated) expression of the angiotensin-converting enzyme 2 (ACE2) in resected lung tissue from COPD patients compared to those with healthy lung function^[9]^. Some published evidence also indicates higher ACE2 expression in smokers compared to never smokers, which suggests that smokers can be more susceptible to infection by the SARS-CoV-2 virus^[9,10]^.

It is necessary to put all these findings into context and consider that people with CLD, especially past or current smokers are more likely to have immune dysregulation. Therefore, these groups of people can be at higher risk of developing more severe symptoms out of a simple upper respiratory infection (similar to Bhat et al. suggestion^[11]^).

**Cardiovascular disease, CVD:** In addition to respiratory complications, published studies are showing the impact of pre-exist CVDs on developing COVID-19 and on worsening its severity and clinical outcomes. Hendren et al. showed that COVID-19 might cause myocarditis-like syndrome and acute myocardial injury associated with reduced left ventricular ejection fraction (LVEF), which can also be complicated by heart failure^[12]^. A different analysis in China showed that 8%-20% of the patients hospitalized with COVID-19 had abnormal cardiac troponin I (cTnI) who were also older and had more comorbid diseases^[13,14]^. There are also published literature (not fully proven though) showing that SARS-CoV-2 can infect fibroblasts and cardiomyocytes via the ACE2-pathway causing myocardial injury^[15-19]^. Moreover, it is shown that patients with viral myocarditis, which commonly follows with chest pain can mimic a ventricular arrhythmia or coronary syndrome^[20,21]^. Historically, researches have shown a significant increase in mortality for SARS patients with pre-existing CVD^[22-27]^.

**Diabetes mellitus, DM:** Historically, DM has been reported as a significant risk factor for higher mortality in patients infected with SARS-coronavirus, MERS-CoV, and H1N1^[28-31]^. The DM prevalence among COVID-19 cases is reported higher than the estimated national prevalence^[2]^.

Most of the published studies have shown a significant association between DM and increased COVID-19 disease severity and MR. For example, in a meta-analysis by Jain and Yuan to estimate the effect of a set of comorbidities on COVID-19 severity in China (March 16, 2020), they showed that diabetes (16.8%) and hypertensive heart disease (25.4%) were the most prevalent comorbidities in confirmed cases^[32]^. In data from Italy, diabetes was reported in 20.3% of COVID-19 deaths^[2,3]^. Moreover, different studies in China showed a higher prevalence of DM among patients with more severe COVID-19^[7,33,34]^.

Pal et al. and Guo et al. discussed few explanations for the association between pre-existing DM and COVID-19 disease severity, including innate immunity and exaggerated proinflammatory cytokine response, notably interleukin (IL)-1, IL-6 and tumor necrosis factor (TNF)-a^[35-39]^. They also mentioned the plausible role of ACE2 in the association between DM and COVID-19 since it plays an essential anti-inflammatory and anti-oxidant role protecting the lung against ARDS^[40]^, as it is proven for 2014 H5N1 infection^[41]^. Furthermore, the reduced expression of ACE2 has been reported for COVID-19 patients with pre-exist DM, possibly due to glycosylation^[40-42]^.

There are few studies suggesting the idea of the potential role of DPP4 inhibition in COVID-19- patients with pre-existing DM. DPP4 enzyme is a transmembrane glycoprotein, expressed in the immune cells and plays a vital role in glucose/insulin metabolism, and can potentially increase the inflammation in DM individuals via both catalytic and noncatalytic mechanisms. Therefore, they discussed the possibility of DPP4 as a potential target to prevent and/or reduce the risk of respiratory complications that may add to the COVID-19 infection^[43-47]^. In contrast, studies are reporting a non-significant effect of pre-existing DM on COVID-19 disease severity^[48]^.

**Chronic liver disease:** The mechanism of COVID-19 in liver disease development remains poorly understood. Therefore, there are not many studies discussing the association between pre-existing liver disease and COVID-19 occurrence and or severity. In a study in Shanghai, China, about 51% of COVID-19 patients had abnormal liver function (LF). Of those, about 30% had pre-existing liver disease, including chronic hepatitis B and C^[49]^. In contrast, Chen et al. reported a similar rate of abnormal LF for both groups of COVID-19 patients with and without pre-existing liver disease in Wuhan, China^[50]^. There are also hypotheses regarding the connection between ACE2 and intestinal inflammation as a cause of experiencing diarrhea in COVID-19 patients, although its viability is not confirmed yet^[51-53]^.

**Demographic & Social Factors:**

**Age:** People of 65 years of age and older are at significantly higher risk of experiencing COVID-19 or for hospitalization and death, especially if they have pre-existing comorbidities such as CVD, DM, CLD, Hypertensive heart disease, and obesity^[54,55]^. Ferguson et al. reported that 27%-71% of patients older than 60 years needed especial care in an ICU with an infection fatality rate of about 2%-9.5%^[56,57]^. Stang et al. discussed a probable bias in age-significance in COVID-19 patients due to overestimation caused by the limited testing capacity to more symptomatic patients. They showed that the fatality rate from COVID-19 started increasing after age 60 years in Italy, Spain, and the USA (as of April 20, 2020)^[58,59]^. There is also a study on children with a median age of 7 years in China (April 1, 2020) in which most of the cases were male (not significant though) with mild symptoms^[60]^.

Although, there is still not enough evidence and/or data to confirm whether this increase in mortality is directly related to age or other comorbidities that are not considered yet in the analysis.

**Gender:** Most evidence suggests that men are infected at a higher rate than women by COVID-19 and exhibit a higher mortality rate. However, most studies showed no significant differences in infection and mortality between men and women COVID-19 cases^[1,61]^. Wenham et al. indicated that although an equal number of male and female COVID-19 cases was observed, there seem to be different MR by gender. They also suggested that women can be in high risk of getting infected, since they have more front-line interaction with communities, and provide more informal care within families besides their physical and cultural differences^[62,63]^.

Further, selected studies report significantly different gender-distributions between male and female COVID-19 cases. For example, Zhao Y et al., using single-cell data, reported that ACE2 was upregulated in Asian males compared to women and other ethnicities which may lead to more severe incidents of COVID-19^[10,64,65]^.

**Smoking status:** Historically, smokers are at higher risk of getting respiratory complications (such as influenza and tuberculosis) besides pulmonary infections, due to lung-damage caused by smoking^[65-68]^. Many publications reported a significant relationship between smoking-status and COVID-19 prevalence and disease severity^[6,7,9,64,69,70]^. Specifically, Zhao Q et al. showed 2-fold increase in COVID-19 severity for smokers^[6]^. Alqahtani et al. reported about 1.5-fold increase in COVID-19 severity and 40% higher MR for current-smokers^[8]^. Wang et al. and Zhao Y, et al. showed upregulation of ACE2 in smokers compared to non-smoker COVID-19 patients^[10,64,71]^. However, it should also be considered that most of the COVID-19 related studies are from China that exhibits a high rate of male-smokers, especially in rural areas (about 50%)^[9,69]^. In contrast, some studies did not confirm a significant association between smoking-status and COVID-19 disease severity^[72]^.

In summary, most of the deaths from COVID-19 were in male-smokers from older age groups and those with underlying conditions such as chronic respiratory disease, cancer, hypertensive heart disease, diabetes, or cardiovascular disease.

**Environmental Factors:**

**Temperature & Humidity:** Data from the early stage of the outbreak in China (during winter) mostly showed that a higher temperature was associated with lower COVID-19 incidence^[73-76]^. Wang J et al. (March 9, 2020) and Qi et al. (April 19, 2020), reported reduced transmission of COVID-19 in higher temperature and humidity^[73,77,78]^.

On the other hand, Juni et al. (January 1, 2020), and Sobur et al. (March 28, 2020) did not confirm any significant relationship between daily temperature and COVID-19 incidence^[79,80]^. s

**Air pollution:** Exposure to air pollution and particulate matter (PM) can have a positive association with increased risk of certain viral respiratory diseases such as influenza and SARS pandemic 2003. Studies are showing that exposure to PM increased the MR from 2009 H1N1 and Spanish influenza^[81-84]^. Air pollution is also linked to cellular damage, inflammation, CVD, and CLD, which are potential comorbidities associated with COVID-19 severity^[81,85-87]^. Ye et al. showed that air pollution could also play a role in infectious disease transmission, although it has not been studied for COVID-19 as of May 15, 2020^[88]^.

Wu et al. and Mollalo et al., in nationwide studies in the USA, showed that exposure to PM increased COVID-19 mortality and severity^[81,89,90]^. Setti et al. reported a significant relationship between PM and experiencing COVID-19 in Italy (January 1, 2020)^[91]^.

Several studies did not confirm the association between air pollution and COVID-19 severity, mortality, and transmission. However, they agreed that since exposure to air pollution and PM has a link with other complications, there can be a risk factor in increasing COVID-19 MR and disease severity^[50,92-94]^.

**Table S1.** Descriptive statistics of comorbidities & disorders, demographics & social factors, and environmental factors, across the contiguous United States (2018-2019)

|  | **Variable** | **Mean±SD or %(N)** | **Min (county, state)** | **Max (county, state)** | **Description** | **Source** |
| --- | --- | --- | --- | --- | --- | --- |
|  | Diabetes | 10.5±3.5 | 2.2 (Teton, WY) | 28.7 (Jackson, AR) | Mortality rates from comorbidities and disorders in 100,000 population | US Diabetes Surveillance System, USDSS (<https://gis.cdc.gov/grasp/diabetes/diabetesatlas.html>) |
| **Comorbidities & Disorders**  (MR in 100,000 population) | CVD | 278±51.6 | 76 (Pitkin, CO) | 545.2 (Franklin Parish, LA) |  | USAFacts (usafacts.org) |
|  | Cardiomyopathy  & myocarditis | 7.90±3.07 | 2.40 (Kings, NY) | 34.7 (La Salle Parish, LA) |  |  |
|  | Hypertensive heart disease | 10.2±7.84 | 1.64 (Summit, CO) | 113.87 (Panola, MS) |  |  |
|  | Peripheral vascular disease | 2.68±1.23 | 0.8 (Queens, NY) | 23.11 (McKean, PA) |  |  |
|  | Atrial fibrillation | 7.5 ±1.84 | 1.67 (Kings, NY) | 44.02 (Carroll, MO) |  |  |
|  | Cerebrovascular disease | 53.4±11.33 | 14.50 (Summit, CO) | 134.64 (Angelina, TX) |  |  |
|  | Hepatitis | 0.22±0.16 | 0.06 (Lincoln, SD) | 5.24 (Union, FL) |  |  |
|  | HIV/AIDS | 1.3±1.93 | 0.15 (St. Croix, WI) | 64.8 (Union, FL) |  |  |
|  | TB | 0.2±0.16 | 0.06 (Daggett, UT) | 3.51 (Oglala Lakota, SD) |  |  |
|  | Lower respiratory infection | 30.35±10.14 | 7.0 (Collier, FL) | 87.7 (East Feliciana Parish, LA) |  |  |
|  | Interstitial lung disease  & pulmonary sarcoidosis | 5.56 ±1.10 | 2.75 (Kings, NY) | 14.93 (Oglala Lakota, SD) |  |  |
|  | Asthma | 1.25±0.38 | 0.49 (Newport, RI) | 4.12 (Holmes, MS) |  |  |
|  | COPD | 55.6±16.14 | 9.90 (Summit, CO) | 152.29 (Magoffin, KY) |  |  |
|  | Ischemia | 174.3±47.17 | 34.50 (Pitkin, CO) | 439.77 (Franklin Parish, LA) |  |  |
|  | Mesothelioma | 0.96±0.43 | 0.4 (Kings, NY) | 4.9 (Carlton, MN) |  |  |
|  | Tracheal cancer | 62.53±17.76 | 10.7 (Summit, CO) | 231.21 (Union, FL) |  |  |
|  | Leukemia | 9.55±0.77 | 4.17 (Summit, CO) | 16.55 (Union, FL) |  |  |
|  | Pancreatic cancer | 12.83±1.48 | 5 (Summit, CO) | 22.75 (Union, FL) |  |  |
|  | Rheumatic disease | 3.43±0.89 | 1.1 (Queens, NY) | 8.9 (Lowndes, AL) |  |  |
|  | Drug use disorder | 9.9±6.07 | 1.62 (Sioux, IA) | 57.15 (Rio Arriba, NM) |  |  |
|  | Alcohol use disorder | 2.9±2.22 | 0.63 (Hidalgo, TX) | 38.8 (Todd, SD) |  |  |
| **Demographics & Social**  (Frequency in total population) | Age (year) | 38.2±7.9 | 28.08 (Chattahoochee, GA) | 60.56 (Sumter, FL) | Median age in years | US Census Bureau Population Estimates, 2018 (census.gov) |
|  | Female-AA% | 0.09% (0.15) | 0.00% (Wibaux, MT) | 0.87% (Claiborne, MS) | Percentage of female African American |  |
|  | Female-WA% | 0.85% (0.16) | 0.51% (Oglala Lakota, SD) | 0.99% (Garfield, NE) | Percentage of female white American |  |
|  | Male-AA% | 0.09% (0.14) | 0.00% (Alpine, CA) | 0.85% (Claiborne, MS) | Percentage of male African American |  |
|  | Male-WA% | 0.85% (0.15) | 0.60% ((Oglala Lakota, SD) | 0.99% (McPherson, NE) | Percentage of male white American |  |
|  | Asian% | 1.4% (2.37) | 0.00% (King, TX) | 36.50% (Santa Clara, CA) | Percentage of Asian |  |
|  | Smokers% | 17.8% (3.6) | 7.00% (Utah, UT) | 41.00% (Oglala Lakota, SD) | Percentage of current smokers |  |
|  | Unemployed% | 5.2% (1.82) | 1.70% (Baca, CO) | 23.50% (Imperial, CA) | Percentage of unemployment | Bureau of Labor Statistics ([bls.gov](https://www.bls.gov/)) |
|  | Income rate | 4.52% (0.72) | 2.70% (Borden, TX) | 8.80% (Monroe, AL) | Ratio of household income at the 80th percentile to income at the 20th percentile | Small Area Income and Poverty Estimates, American Community Survey, five-year Estimates (census.gov) |
|  | Uninsured% | 12.0% (5.02) | 2.00% (Norfolk, MA) | 33.00% (Collingsworth, TX) | Percentage of uninsured | Small Area Health Insurance Estimates (census.gov) |
|  | Food insecurity | 14.2±4.2 | 3 (Grant, KS) | 38 (Jefferson, MS) | Food Environment Index (2018) | Map the Meal Gap  (<https://map.feedingamerica.org/>) |
|  | Fair/Poor health | 17.5±4.7 | 8 (Douglas, CO) | 41 (Starr, TX) | Percentage of adults that report fair or poor health (2018) | Behavioral Risk Factor Surveillance System, BRFSS  (<https://www.cdc.gov/brfss/>) |
| **Environmental** | Population density | 173.0±739.4 | 0.2 (Kings, NY) | 27154.2 (Loving, TX) | Population density per square miles | US Census Bureau Population Estimates, 2018 (census.gov) |
|  | AQI | 27.8±6.00 | 7.95 (Teton, WY) | 54.5 (Neosho, KS) | Air quality index | US Environmental Protection Agency, EPA ([epa.gov](https://www.epa.gov/)) |
|  | Temperature (°C) | 38.9±9.88 | 13.38 (Lake, MN) | 71.69 (Monroe, FL) | Weather (temperature) | National Oceanic and Atmospheric Administration, NOAA ([noaa.gov](https://www.noaa.gov/)) |
|  | PM ($\mu g/m^{3})$ | 6.8±1.56 | 1.77 (Teton, WY) | 17.15 (Neosho, KS) | Particulate matter 2.5 | US Environmental Protection Agency, EPA ([epa.gov](https://www.epa.gov/)) |

**Table S2.** **1^st^ wave (March 25 – Jun 3, 2020).** Parameter estimates and fit indices for linear and quadratic LGMs using COVID-19 MIR. Estimated intercepts show the initial COVID-19 mortality to incidence (MIR) at the beginning of the wave on March 25. Estimated slopes indicate the COVID-19 growth trajectories (pattern) over time from March 25 to June 3, 2020, USA

| **Estimates/Fit** | **Parameter/Statistics** | **linear LGM** | **Non-linear LGM** | |
| --- | --- | --- | --- | --- |
|  |  | Mean (SE) | Mean (SE) | |
| **Estimate** | Intercept | 0.040 (0.004)^*^ | 0.020 (0.030)^*^ | |
|  | Slope | 0.025 (0.005)^*^ | 0.110 (0.011)^*^ | |
|  | Quadratic Slope^**^ | *NA* | -0.075 (0.009)^*^ | |
| **Factor Loading** |  | slope | slope | Quadratic Slope |
|  | 1^st^ | 0.00 | 0.00 | 0.00 |
|  | 2^nd^ | 0.10 | 0.10 | 0.01 |
|  | 3^rd^ | 0.20 | 0.20 | 0.04 |
|  | 4^th^ | 0.30 | 0.30 | 0.09 |
|  | 5t^h^ | 0.40 | 0.40 | 0.16 |
|  | 6t^h^ | 0.50 | 0.50 | 0.25 |
|  | 7^th^ | 0.60 | 0.60 | 0.36 |
|  | 8^th^ | 0.70 | 0.70 | 0.49 |
|  | 9^th^ | 0.80 | 0.80 | 0.64 |
|  | 10^th^ | 0.90 | 0.90 | 0.81 |
|  | 11^th^ | 1.00 | 1.00 | 1.00 |
| **Fit Index** | AIC | -50555.3 | -53369.9 | |
| ^* Significant with P-values less than 0.05.^ | | | | |

**Table S3.** **2^nd^ wave (Jun 4 – Sep 2, 2020).** Parameter estimates and fit indices for linear and quadratic LGMs using COVID-19 MIR. Estimated intercepts show the initial COVID-19 mortality to incidence (MIR) at the beginning of the wave on Jun 4. Estimated slopes indicate the COVID-19 growth trajectories (pattern) over time from Jun 4 to Sep 2, 2020, USA

| **Estimates/Fit** | **Parameter/Statistics** | **linear LGM** | **Non-linear LGM** | |
| --- | --- | --- | --- | --- |
|  |  | Mean (SE) | Mean (SE) | |
| **Estimate** | Intercept | 0.029 (0.002)^*^ | 0.036 (0.002)^*^ | |
|  | Slope | -0.010 (0.005)^*^ | -0.073 (0.007)^*^ | |
|  | Quadratic Slope | *NA* | 0.090 (0.008)^*^ | |
| **Factor Loading** |  | slope | slope | Quadratic Slope |
|  | 1^st^ | 0.00 | 0.00 | 0.00 |
|  | 2^nd^ | 0.10 | 0.10 | 0.01 |
|  | 3^rd^ | 0.20 | 0.20 | 0.04 |
|  | 4^th^ | 0.30 | 0.30 | 0.09 |
|  | 5t^h^ | 0.40 | 0.40 | 0.16 |
|  | 6t^h^ | 0.50 | 0.50 | 0.25 |
|  | 7^th^ | 0.60 | 0.60 | 0.36 |
| **Fit Index** | AIC | -42524.4 | -43653.7 | |
| ^* Significant with P-values less than 0.05.^ | | | | |

**Table S4.** **3^rd^ wave (Sep 3 – Nov 12, 2020).** Parameter estimates and fit indices for linear and quadratic LGMs using COVID-19 MIR. Estimated intercepts show the initial COVID-19 mortality to incidence (MIR) at the beginning of the wave on Sep 3. Estimated slopes indicate the COVID-19 growth trajectories (pattern) over time from Sep 3 to Nov 12, 2020, USA

| **Estimates/Fit** | **Parameter/Statistics** | **linear LGM** | **Non-linear LGM** | |
| --- | --- | --- | --- | --- |
|  |  | Mean (SE) | Mean (SE) | |
| **Estimate** | Intercept | 0.020 (0.001) | 0.020 (0.001) | |
|  | Slope | -0.012 (0.002) | -0.003 (0.004) | |
|  | Quadratic Slope | *NA* | -0.030 (0.011) | |
| **Factor Loading** |  | slope | slope | Quadratic Slope |
|  | 1^st^ | 0.0 | 0.0 | 0.00 |
|  | 2^nd^ | 0.1 | 0.1 | 0.01 |
|  | 3^rd^ | 0.2 | 0.2 | 0.04 |
|  | 4^th^ | 0.3 | 0.3 | 0.09 |
| **Fit Index** | AIC | -23336.8 | -23764.6 | |
| ^* Significant with P-values less than 0.05.^ | | | | |

**Table S5.** The goodness of fit indices for the latent growth mixture models (LGMMs) to find the optimal number of the clusters based on the COVID-19 MIR in each wave. 1^st^ wave (March 25 – Jun 3, 2020) includes 1736 counties (with MIR>0), 2^nd^ wave (Jun 4 - Sep 2, 2020) includes 1188 counties (with MIR>0) in the sunbelt region, and 3^rd^ wave (Sep 3 – Nov 12, 2020) includes 944 counties (with MIR>0) in the great plains region, USA

| **Wave** | **Model** | **Cluster Sample-size^*^** | **AIC** | **BLRT P-value** |
| --- | --- | --- | --- | --- |
| **1^st^** | 1 cluster | 1736 | -54871.4 | *NA* |
|  | 2 cluster | (225, 1511)^*^ | -58691.2 | < 0.001 |
|  | 3 cluster | (93, 127, 1516) | -59237.4 | < 0.001 |
|  | 4 cluster | (193, 80, 7, 1289) | -60294.9 | < 0.001 |
|  | 5 cluster | (9, 1181, 207, 268, 71) | -61555.3 | < 0.001 |
|  | 6 cluster | (26, 28, 1372, 101, 5, 207) | -63502.5 | < 0.001 |
|  | 7 cluster | (1094, 6, 102, 374, 5, 30, 125) | -64010.5 | 0.001 |
|  | **8 cluster** | **(52, 74, 66, 39, 1406, 64, 12, 23)** | **-64292.3** | **0.010** |
|  | 9 cluster | (5, 61, 1209, 10, 201, 37, 93, 12, 108) | -64901.8 | 0.091 |
| **2^nd^** | 1 cluster | 1344 | 44928.1 | *NA* |
|  | 2 cluster | (1129,59) | -46180.0 | < 0.001 |
|  | 3 cluster | (53, 1046, 89) | -47810.9 | < 0.001 |
|  | 4 cluster | (33, 72, 1057, 26) | -48706.1 | < 0.001 |
|  | 5 cluster | **(32, 1035, 43, 59, 19)** | -49787.7 | < 0.001 |
|  | 6 cluster | (43, 60, 3, 1030, 19, 33) | -50113.3 | < 0.001 |
| **3^rd^** | 1 cluster | 1055 | -17113.9 | *NA* |
|  | 2 cluster | (187, 757) | -18259.5 | < 0.001 |
|  | 3 cluster | (759, 148, 37) | -18534.6 | < 0.001 |
|  | 4 cluster | (125, 47, 11, 761) | -24913.5 | < 0.001 |
|  | 5 cluster | (75, 91, 18, 9, 751) | -18817.1 | < 0.001 |
| ^* Number of the counties in each cluster. For instance, (225, 1511) indicates: (Number of the counties in cluster1, Number of the counties in cluster2) in a 2-cluster model.^ | | | | |

To identify counties with similar COVID-19 MIR growth trajectories over time, we fitted a series of LGMMs with different cluster numbers (Table S3) for each wave.

In the **1^st^ wave**, the BLRT suggested that each successive model above the 1-cluster model provided statistical improvement in the fit (e.g., a four-cluster model was better than a three-cluster one, which was better than a two-cluster model, etc.) up to a 9-cluster model (BLRT P-value<0.1). This result is consistent with the information criteria as well. The cluster sample sizes, however, suggested that models with 8 clusters had an adequate cluster sample size (>1% population in each cluster). Therefore, an 8-cluster non-linear LGMM was selected as the best model to find clusters of the U.S. counties.

In the **2^nd^ wave**, the BLRT showed that each successive model above the 1-cluster model provided statistical improvement in the fit up to a 6-cluster model. This result consistent with the information criteria as well. The cluster sample size, however, suggested that models with 5 clusters had an adequate cluster sample size (>1% population in each cluster). Therefore, a 5-cluster non-linear LGMM was selected as the best model to find clusters of the U.S. counties in the sunbelt region during the 2^nd^ wave (Jun 4 to Sep 2, 2020).

In the **3^rd^ wave**, both information criteria and cluster sample size suggested that each successive model above the 1-cluster model provided statistical improvement in the fit up to a 4 model. Therefore, a 4-cluster non-linear LGMM was selected as the best model to find clusters of the U.S. counties in the great plains region during the 3^rd^ wave (Sep 3 to Nov 12, 2020).

**Table S6. GLMM clustering results**. Clustering (based on COVID-19 MIR>0) of the 1736 counties during the 1^st^ wave (March 25 – Jun 3, 2020), 1344 targeted counties (sunbelt region) during the 2^nd^ wave (Jun 4 - Sep 2, 2020), and 1055 targeted counties (great plains region) during the 3^rd^ wave (Sep 3 – Nov 12, 2020), USA (as a complementary for Table 4)

| **Wave** | **Cluster** | **Intercept^*^** | | **Slope^**^** | | **Factor Loadings** |
| --- | --- | --- | --- | --- | --- | --- |
|  |  | Mean (SE) | P-value | Mean (SE) | P-value | (March 25, April 1, April 8, April 15, April 22, April 29, May 6, May 13, May20, May 27, June 3) |
| **1^st^** | **0** | 0%(0%) | *NA* | 0% (0%) | *NA* | *NA* |
|  | **1** | 12.9%(3.1%) | <0.001 | -1.0%(0.6%) | 0.122 | 0, 5.13, 5.21, 3.97, 3.74, 3.65, 3.05, -0.61, -0.35, -0.31, -0.29 |
|  | **2** | 2.2%(0.8%) | 0.010 | 3.5%(1.0%) | <0.001 | 0, -0.38, 2.01, 2.55, 3.39, 3.68, 3.73, 3.55, 3.24, 0.12, 1.96 |
|  | **3** | 1.9%(0.9%) | 0.027 | 2.8%(0.4%) | <0.001 | 0, -0.42, 1.78, 6.49, 7.33, 4.66, 3.50, 2.46, 1.97, 0.09, 1.34 |
|  | **4** | 0.9%(0.5%) | 0.089 | 2.0%(0.4%) | <0.001 | 0, -0.09, -0.18, -0.16, -0.16, -0.19, -0.18, -0.24, -5.23, 3.07, -4.83 |
|  | **5** | 1.0%(0.3%) | <0.001 | -3.0%(0.5%) | <0.001 | 0, -0.13, -0.41, -0.60, -0.80, -1.00, -1.14, -1.17, -1.21, -1.16 |
|  | **6** | 9.8%(3.0%) | 0.001 | 3.4%(0.7%) | <0.001 | 0, 7.78, 2.76, 0.84, -0.09, -0.30, -0.45, -0.51, -0.59, -0.54, -0.62 |
|  | **7** | 1.5%(1.3%) | 0.236 | -3.1%(0.5%) | <0.001 | 0, 0.07, 0.06, 0.06, -0.68, -8.05, -4.23, -3.97, -3.59, -3.24, -3.31 |
|  | **8** | 1.9%(1.3%) | 0.127 | -4.2%(0.0%) | *NA* | 0, 0.13, 0.15, 0.03, -0.04, -0.06, -3.97, -3.21, -2.56, -2.34, -1.97 |
|  | **Cluster** | **Intercept** | | **Slope** | | **Factor Loadings**  (Jun 4, Jun 18, Jul 2, Jul 16, Jul 30, Aug 13, Aug 27) |
|  |  | Mean (SE) | P-value | Mean (SE) | P-value |  |
| **2^nd^** | **0** | 0%(0%) | NA | 0%(0%) | *NA* | *NA* |
|  | **1** | 1.5%(0.3%) | <0.001 | 10.6%(4.5%) | 0.155 | 0, -0.07, -0.08, -0.08, 0.45, 0.42, 0.31 |
|  | **2** | 3.0%(0.2%) | <0.001 | 12.5%(4.3%) | 0.0014 | 0, -0.02, -0.05, -0.08, -0.09, -0.09, -0.09 |
|  | **3** | 10.6%(1.8%) | <0.001 | 20.7%(12.7%) | 0.102 | 0, 0.05, 0.05, -0.07, -0.11, -0.12, -0.12 |
|  | **4** | 1.8%(0.3%) | <0.001 | 16.3%(6.5%) | 0.012 | 0, -0.07, -0.07, -0.07, -0.04, 0.11, 0.09 |
|  | **5** | 14.1%(4.5%) | <0.001 | 74.7%(0.0%) | *NA* | 0, 0.01, -0.10, -0.12, -0.13, -0.13, -0.13 |
|  | **Cluster** | **Intercept** | | **Slope** | | **Factor Loadings**  (Sep 3, Sep 17, Oct 15, Nov 12) |
|  |  | Mean (SE) | P-value | Mean (SE) | P-value |  |
| **3^rd^** | **0** | 0%(0%) | *NA* | 0%(0%) | *NA* | *NA* |
|  | **1** | 5.2%(0.2%) | <0.001 | -3.7%(0.5%) | <0.001 | 0, 0.02, 0.19, 0.41 |
|  | **2** | 1.0%(0.6%) | 0.082 | 3.1%(1.8%) | 0.088 | 0, 0.04, 0.68, 0.47 |
|  | **3** | 3.5%(1.1%) | 0.002 | -20.0%(3.2%) | <0.001 | 0, -0.20, -0.15, -0.01 |
|  | **4** | 1.4% (0.1%) | <0.001 | -0.9%(0.3%) | 0.001 | 0, 0.11, 0.20, 0.30 |
| ^* Intercept indicates the estimated mean COVID-19 MIR at the beginning of the study on March 25, 2020, for each cluster.^  ^** Slope indicates the overall change of COVID-19 MIR over the study time, for each cluster.^ | | | | | | |

**Table S7. 1^st^ Wave (March 25 to June 3, 2020, USA):** estimation of COVID-19 MIR and the rank of each cluster based on these rates. Estimations are based on the LGMM with 8 cluster

| **Cluster Number** | COVID-19 MIR on March 25 | Cluster Rank  (March 25) | Change in COVID-19 MIR  During the 1^st^ wave | COVID-19 MIR on June 3 | Cluster Rank  (June 3) |
| --- | --- | --- | --- | --- | --- |
| **1** | 12.9% | 1^*^ | 0.3% ↑ | 13.2% | 1 |
| **2** | 2.2% | 3 | 6.9% ↑ | 9.1% | 5 |
| **3** | 1.9% | 4 | 3.7% ↑ | 5.6% | 7 |
| **4** | 0.8% | 7 | 9.7% ↑ | 10.5% | 3 |
| **5** | 1.0% | 6 | 3.5% ↑ | 4.5% | 8 |
| **6** | 9.8% | 2 | 2.1% ↓ | 7.7% | 6 |
| **7** | 1.5% | 5 | **10.1% ↑** | 11.6% | 2 |
| **8** | 1.9% | 4 | 8.3%↑ | 10.2% | 4 |
| ^* Rank #1 means the worst cluster with the highest COVID-19 MIR.^ | | | | | |

**Table S8. 2^nd^ wave (Jun 4 to Sep 2, 2020, USA):** estimation of COVID-19 MIR and the rank of each cluster based on these rates. Estimations are based on the LGMM with 5 cluster

| **Cluster Number** | COVID-19 MIR on Jun 4 | Cluster Rank  (Jun 4) | Change in COVID-19 MIR  During the 2^nd^ wave | COVID-19 MIR on Sep 2 | Cluster Rank  (Sep 2) |
| --- | --- | --- | --- | --- | --- |
| **1** | 1.5% | 5 | **3.3% ↑** | 4.8% | 2 |
| **2** | 3.0% | 3 | 1.2% ↓ | 1.8% | 5 |
| **3** | 10.6% | 2 | 2.6% ↓ | 8.0% | 1 |
| **4** | 1.8% | 4 | 1.5% ↑ | 3.3% | 4 |
| **5** | 14.1% | 1 | 10.1% ↓ | 4.0% | 3 |
| ^* Rank #1 means the worst cluster with the highest COVID-19 MIR.^ | | | | | |

**Table S9. 3^rd^ wave (Sep 3 - Nov 12, 2020, USA):** estimation of COVID-19 MIR and the rank of each cluster based on these rates. Estimations are based on the LGMM with 4 cluster

| **Cluster Number** | COVID-19 MIR on Sep 3 | Cluster Rank  (Sep 3) | Change in COVID-19 MIR  During the 3^rd^ wave | COVID-19 MIR on Nov 12 | Cluster Rank  (Nov 12) |
| --- | --- | --- | --- | --- | --- |
| **1** | 5.2% | 1 | 1.6% ↓ | 3.6% | 2 |
| **2** | 1.0% | 4 | **1.5% ↑** | 2.5% | 3 |
| **3** | 3.5% | 2 | 0.3% ↑ | 3.8% | 1 |
| **4** | 1.4% | 3 | 0.2% ↓ | 1.2% | 4 |
| ^* Rank #1 means the worst cluster with the highest COVID-19 MIR.^ | | | | | |

**Table S10.** **1^st^ Wave (March 25 to June 3, 2020, USA):** results from multinomial logit model for all clusters. The estimated odds ratios (ORs) show the association of each risk factor with increasing/decreasing the relative log odds of being in each cluster compare to cluster 0 (MIR=0)

|  | **Variable** | **cluster** | **Est.** | **SD** | **OR** | **P-value** |
| --- | --- | --- | --- | --- | --- | --- |
| **Comorbidities & Disorders** | CVD | 1 | 0.025 | 0.062 | 1.025 | 0.689 |
|  |  | 2 | -0.085 | 0.058 | 0.918 | 0.140 |
|  |  | 3 | -0.053 | 0.057 | 0.949 | 0.352 |
|  |  | 4 | -0.003 | 0.063 | 0.997 | 0.960 |
|  |  | 5 | 0.072 | 0.025 | 1.075 | **0.004** |
|  |  | 6 | 0.03 | 0.052 | 1.03 | 0.561 |
|  |  | 7 | -0.285 | 0.135 | 0.752 | **0.035** |
|  |  | 8 | -0.072 | 0.072 | 0.93 | 0.316 |
|  | Cardiomyopathy & myocarditis | 1 | 0.003 | 0.089 | 1.003 | 0.974 |
|  |  | 2 | 0.079 | 0.079 | 1.082 | 0.318 |
|  |  | 3 | 0.081 | 0.075 | 1.084 | 0.282 |
|  |  | 4 | 0.117 | 0.08 | 1.124 | 0.142 |
|  |  | 5 | -0.001 | 0.035 | 0.999 | 0.980 |
|  |  | 6 | -0.052 | 0.073 | 0.949 | 0.475 |
|  |  | 7 | 0.1 | 0.212 | 1.105 | 0.639 |
|  |  | 8 | 0.18 | 0.093 | 1.198 | 0.053 |
|  | Hypertensive heart disease | 1 | 0.015 | 0.064 | 1.015 | 0.818 |
|  |  | 2 | 0.092 | 0.06 | 1.097 | 0.126 |
|  |  | 3 | 0.066 | 0.059 | 1.068 | 0.268 |
|  |  | 4 | 0.023 | 0.068 | 1.024 | 0.732 |
|  |  | 5 | -0.02 | 0.027 | 0.98 | 0.452 |
|  |  | 6 | 0.009 | 0.054 | 1.009 | 0.864 |
|  |  | 7 | 0.314 | 0.14 | 1.369 | **0.025** |
|  |  | 8 | 0.121 | 0.076 | 1.129 | 0.110 |
|  | Peripheral vascular disease | 1 | 0.227 | 0.148 | 1.255 | 0.126 |
|  |  | 2 | 0.183 | 0.212 | 1.201 | 0.389 |
|  |  | 3 | 0.235 | 0.175 | 1.264 | 0.181 |
|  |  | 4 | 0.024 | 0.276 | 1.025 | 0.93 |
|  |  | 5 | -0.704 | 0.123 | 0.495 | **<0.001** |
|  |  | 6 | 0.212 | 0.138 | 1.236 | 0.125 |
|  |  | 7 | 0.499 | 0.471 | 1.648 | 0.289 |
|  |  | 8 | -0.148 | 0.359 | 0.863 | 0.681 |
|  | Atrial fibrillation | 1 | 0.028 | 0.09 | 1.028 | 0.759 |
|  |  | 2 | 0.044 | 0.111 | 1.045 | 0.689 |
|  |  | 3 | 0.025 | 0.111 | 1.025 | 0.823 |
|  |  | 4 | -0.063 | 0.124 | 0.939 | 0.608 |
|  |  | 5 | -0.202 | 0.05 | 0.817 | **<0.001** |
|  |  | 6 | -0.043 | 0.102 | 0.958 | 0.676 |
|  |  | 7 | 0.286 | 0.26 | 1.331 | 0.271 |
|  |  | 8 | 0.121 | 0.119 | 1.128 | 0.309 |
|  | Cerebrovascular | 1 | -0.05 | 0.067 | 0.951 | 0.453 |
|  |  | 2 | 0.089 | 0.061 | 1.093 | 0.145 |
|  |  | 3 | 0.065 | 0.06 | 1.067 | 0.274 |
|  |  | 4 | -0.001 | 0.068 | 0.999 | 0.994 |
|  |  | 5 | -0.09 | 0.027 | 0.914 | **0.001** |
|  |  | 6 | -0.034 | 0.055 | 0.966 | 0.532 |
|  |  | 7 | 0.309 | 0.14 | 1.362 | **0.027** |
|  |  | 8 | 0.08 | 0.077 | 1.083 | 0.298 |
|  | Diabetes | 1 | 0.046 | 0.044 | 1.047 | 0.293 |
|  |  | 2 | 0.015 | 0.041 | 1.015 | 0.715 |
|  |  | 3 | -0.021 | 0.041 | 0.979 | 0.606 |
|  |  | 4 | 0.103 | 0.044 | 1.108 | **0.019** |
|  |  | 5 | 0.018 | 0.019 | 1.018 | 0.351 |
|  |  | 6 | -0.053 | 0.041 | 0.948 | 0.197 |
|  |  | 7 | 0.026 | 0.07 | 1.026 | 0.714 |
|  |  | 8 | -0.019 | 0.056 | 0.982 | 0.741 |
|  | Hepatitis | 1 | -1.147 | 0.097 | 0.318 | **<0.001** |
|  |  | 2 | 0.727 | 0.098 | 2.069 | **<0.001** |
|  |  | 3 | -0.595 | 0.12 | 0.552 | **<0.001** |
|  |  | 4 | 1.575 | 0.165 | 4.831 | **<0.001** |
|  |  | 5 | 2.368 | 0.621 | 10.678 | **<0.001** |
|  |  | 6 | -1.89 | 0.078 | 0.151 | **<0.001** |
|  |  | 7 | 0.089 | 0.019 | 1.093 | **<0.001** |
|  |  | 8 | 0.455 | 0.093 | 1.576 | **<0.001** |
|  | HIV/AIDS | 1 | 0.157 | 0.207 | 1.17 | 0.448 |
|  |  | 2 | -0.044 | 0.169 | 0.957 | 0.793 |
|  |  | 3 | -0.019 | 0.158 | 0.981 | 0.903 |
|  |  | 4 | 0.022 | 0.217 | 1.023 | 0.918 |
|  |  | 5 | 0.044 | 0.101 | 1.045 | 0.663 |
|  |  | 6 | 0.188 | 0.14 | 1.207 | 0.177 |
|  |  | 7 | 0.082 | 0.384 | 1.086 | 0.831 |
|  |  | 8 | -0.004 | 0.305 | 0.996 | 0.990 |
|  | Tuberculosis | 1 | -0.369 | 0.071 | 0.691 | **<0.001** |
|  |  | 2 | 0.043 | 0.072 | 1.044 | 0.549 |
|  |  | 3 | 0.848 | 0.075 | 2.335 | **<0.001** |
|  |  | 4 | -0.541 | 0.041 | 0.582 | **<0.001** |
|  |  | 5 | 0.135 | 0.64 | 1.145 | 0.832 |
|  |  | 6 | 0.275 | 0.1 | 1.317 | **0.006** |
|  |  | 7 | 0.442 | 0.042 | 1.555 | **<0.001** |
|  |  | 8 | 0.225 | 0.226 | 1.253 | 0.319 |
|  | Lower respiratory infection | 1 | -0.075 | 0.024 | 0.928 | **0.002** |
|  |  | 2 | -0.017 | 0.018 | 0.983 | 0.351 |
|  |  | 3 | 0.001 | 0.017 | 1.001 | 0.968 |
|  |  | 4 | -0.016 | 0.021 | 0.984 | 0.427 |
|  |  | 5 | -0.012 | 0.008 | 0.988 | 0.130 |
|  |  | 6 | -0.011 | 0.017 | 0.989 | 0.527 |
|  |  | 7 | 0.016 | 0.032 | 1.016 | 0.614 |
|  |  | 8 | 0.031 | 0.019 | 1.032 | 0.097 |
|  | Interstitial lung disease & pulmonary sarcoidosis | 1 | -0.256 | 0.17 | 0.774 | 0.133 |
|  |  | 2 | -0.042 | 0.134 | 0.959 | 0.751 |
|  |  | 3 | -0.044 | 0.132 | 0.957 | 0.737 |
|  |  | 4 | -0.182 | 0.164 | 0.833 | 0.268 |
|  |  | 5 | 0.102 | 0.062 | 1.108 | 0.102 |
|  |  | 6 | 0.116 | 0.123 | 1.124 | 0.344 |
|  |  | 7 | -0.038 | 0.28 | 0.962 | 0.891 |
|  |  | 8 | 0.069 | 0.167 | 1.072 | 0.677 |
|  | Ischemia | 1 | -0.025 | 0.061 | 0.975 | 0.683 |
|  |  | 2 | 0.086 | 0.057 | 1.09 | 0.133 |
|  |  | 3 | 0.045 | 0.057 | 1.046 | 0.432 |
|  |  | 4 | 0.002 | 0.063 | 1.002 | 0.971 |
|  |  | 5 | -0.073 | 0.025 | 0.93 | **0.004** |
|  |  | 6 | -0.031 | 0.052 | 0.97 | 0.553 |
|  |  | 7 | 0.277 | 0.135 | 1.319 | **0.041** |
|  |  | 8 | 0.074 | 0.072 | 1.077 | 0.302 |
|  | Mesothelioma | 1 | -1.616 | 0.766 | 0.199 | **0.035** |
|  |  | 2 | 0.759 | 0.365 | 2.136 | **0.037** |
|  |  | 3 | 0.436 | 0.452 | 1.547 | 0.335 |
|  |  | 4 | 1.2 | 0.409 | 3.319 | **0.003** |
|  |  | 5 | -0.015 | 0.236 | 0.985 | 0.948 |
|  |  | 6 | -0.17 | 0.474 | 0.844 | 0.72 |
|  |  | 7 | -0.186 | 0.041 | 0.83 | **<0.001** |
|  |  | 8 | 1.298 | 0.407 | 3.66 | **0.001** |
|  | Tracheal cancer | 1 | 0.012 | 0.016 | 1.012 | 0.428 |
|  |  | 2 | -0.022 | 0.014 | 0.978 | 0.129 |
|  |  | 3 | 0.003 | 0.013 | 1.003 | 0.843 |
|  |  | 4 | 0.016 | 0.016 | 1.017 | 0.302 |
|  |  | 5 | 0.006 | 0.007 | 1.006 | 0.400 |
|  |  | 6 | 0.003 | 0.013 | 1.003 | 0.837 |
|  |  | 7 | 0 | 0.028 | 1 | 0.987 |
|  |  | 8 | -0.01 | 0.018 | 0.99 | 0.596 |
|  | Pancreatic cancer | 1 | 0.164 | 0.16 | 1.178 | 0.306 |
|  |  | 2 | 0.406 | 0.129 | 1.501 | **0.002** |
|  |  | 3 | 0.121 | 0.132 | 1.129 | 0.358 |
|  |  | 4 | -0.094 | 0.158 | 0.91 | 0.551 |
|  |  | 5 | 0.081 | 0.074 | 1.084 | 0.274 |
|  |  | 6 | 0.07 | 0.128 | 1.072 | 0.588 |
|  |  | 7 | 0.514 | 0.255 | 1.671 | **0.044** |
|  |  | 8 | -0.128 | 0.187 | 0.88 | 0.496 |
|  | Drug use disorder | 1 | 0.024 | 0.034 | 1.024 | 0.483 |
|  |  | 2 | 0.054 | 0.03 | 1.055 | 0.073 |
|  |  | 3 | 0.078 | 0.025 | 1.081 | **0.002** |
|  |  | 4 | -0.006 | 0.035 | 0.994 | 0.869 |
|  |  | 5 | 0.01 | 0.014 | 1.01 | 0.476 |
|  |  | 6 | 0.077 | 0.026 | 1.08 | **0.003** |
|  |  | 7 | 0.094 | 0.047 | 1.099 | **0.044** |
|  |  | 8 | 0.032 | 0.034 | 1.033 | 0.338 |
|  | Alcohol use disorder | 1 | -0.1 | 0.123 | 0.905 | 0.414 |
|  |  | 2 | -0.173 | 0.091 | 0.841 | 0.057 |
|  |  | 3 | -0.208 | 0.102 | 0.812 | **0.041** |
|  |  | 4 | -0.099 | 0.102 | 0.906 | 0.334 |
|  |  | 5 | -0.006 | 0.035 | 0.994 | 0.857 |
|  |  | 6 | -0.114 | 0.095 | 0.892 | 0.228 |
|  |  | 7 | -0.011 | 0.122 | 0.989 | 0.925 |
|  |  | 8 | -0.073 | 0.079 | 0.929 | 0.353 |
| **Demographics & Social** | Female-AA% | 1 | -0.29 | 0.215 | 0.749 | 0.177 |
|  |  | 2 | 0.722 | 0.207 | 2.058 | **<0.001** |
|  |  | 3 | 0.726 | 0.221 | 2.066 | **0.001** |
|  |  | 4 | 0.537 | 0.211 | 1.711 | **0.011** |
|  |  | 5 | 2.883 | 0.485 | 17.863 | **<0.001** |
|  |  | 6 | 1.096 | 0.218 | 2.991 | **<0.001** |
|  |  | 7 | -0.081 | 0.029 | 0.922 | **0.006** |
|  |  | 8 | -0.052 | 0.107 | 0.949 | 0.628 |
|  | Female-WA% | 1 | -1.543 | 0.492 | 0.214 | **0.002** |
|  |  | 2 | -2.746 | 0.434 | 0.064 | **<0.001** |
|  |  | 3 | -2.363 | 0.428 | 0.094 | **<0.001** |
|  |  | 4 | -1.27 | 0.46 | 0.281 | **0.006** |
|  |  | 5 | -2.227 | 0.251 | 0.108 | **<0.001** |
|  |  | 6 | -2.415 | 0.429 | 0.089 | **<0.001** |
|  |  | 7 | -0.769 | 0.049 | 0.463 | **<0.001** |
|  |  | 8 | -1.008 | 0.488 | 0.365 | **0.039** |
|  | Male-AA% | 1 | -0.178 | 0.209 | 0.837 | 0.393 |
|  |  | 2 | 0.939 | 0.198 | 2.557 | **<0.001** |
|  |  | 3 | 0.447 | 0.209 | 1.564 | **0.032** |
|  |  | 4 | 0.395 | 0.209 | 1.484 | 0.058 |
|  |  | 5 | 1.617 | 0.486 | 5.038 | **0.001** |
|  |  | 6 | 1.104 | 0.211 | 3.016 | **<0.001** |
|  |  | 7 | -0.18 | 0.029 | 0.835 | **<0.001** |
|  |  | 8 | -0.242 | 0.108 | 0.785 | **0.025** |
|  | Male-WA% | 1 | -1.682 | 0.486 | 0.186 | **0.001** |
|  |  | 2 | -2.944 | 0.424 | 0.053 | **<0.001** |
|  |  | 3 | -2.113 | 0.415 | 0.121 | **<0.001** |
|  |  | 4 | -1.176 | 0.456 | 0.309 | **0.01** |
|  |  | 5 | -0.976 | 0.256 | 0.377 | **<0.001** |
|  |  | 6 | -2.363 | 0.422 | 0.094 | **<0.001** |
|  |  | 7 | -0.662 | 0.049 | 0.516 | **<0.001** |
|  |  | 8 | -0.811 | 0.484 | 0.444 | 0.094 |
|  | Asian% | 1 | 0.248 | 0.152 | 1.281 | 0.103 |
|  |  | 2 | -0.582 | 0.277 | 0.559 | **0.036** |
|  |  | 3 | 0.042 | 0.163 | 1.043 | 0.796 |
|  |  | 4 | 0.267 | 0.14 | 1.306 | 0.057 |
|  |  | 5 | 0.254 | 0.073 | 1.289 | **0.001** |
|  |  | 6 | 0.049 | 0.162 | 1.051 | 0.761 |
|  |  | 7 | 0.248 | 0.238 | 1.281 | 0.298 |
|  |  | 8 | -0.206 | 0.306 | 0.814 | 0.502 |
|  | Smokers% | 1 | 0.022 | 0.083 | 1.022 | 0.790 |
|  |  | 2 | 0.095 | 0.072 | 1.1 | 0.184 |
|  |  | 3 | -0.049 | 0.07 | 0.953 | 0.489 |
|  |  | 4 | -0.144 | 0.083 | 0.866 | 0.084 |
|  |  | 5 | 0.016 | 0.034 | 1.016 | 0.644 |
|  |  | 6 | 0.08 | 0.073 | 1.084 | 0.270 |
|  |  | 7 | 0.081 | 0.132 | 1.084 | 0.540 |
|  |  | 8 | -0.002 | 0.083 | 0.998 | 0.985 |
|  | Unemployed% | 1 | 0.051 | 0.111 | 1.052 | 0.649 |
|  |  | 2 | 0.013 | 0.103 | 1.013 | 0.899 |
|  |  | 3 | -0.166 | 0.102 | 0.847 | 0.103 |
|  |  | 4 | 0.116 | 0.084 | 1.123 | 0.165 |
|  |  | 5 | 0.065 | 0.044 | 1.067 | 0.139 |
|  |  | 6 | 0.03 | 0.103 | 1.03 | 0.772 |
|  |  | 7 | -0.127 | 0.177 | 0.881 | 0.474 |
|  |  | 8 | -0.121 | 0.127 | 0.886 | 0.339 |
|  | Income Rate | 1 | 0.127 | 0.237 | 1.135 | 0.592 |
|  |  | 2 | 0.142 | 0.217 | 1.153 | 0.512 |
|  |  | 3 | 0.108 | 0.204 | 1.115 | 0.595 |
|  |  | 4 | -0.018 | 0.235 | 0.982 | 0.938 |
|  |  | 5 | 0.047 | 0.104 | 1.049 | 0.647 |
|  |  | 6 | -0.011 | 0.217 | 0.989 | 0.958 |
|  |  | 7 | -1.068 | 0.416 | 0.344 | **0.010** |
|  |  | 8 | 0.014 | 0.266 | 1.015 | 0.957 |
|  | Uninsured% | 1 | 0.04 | 0.038 | 1.041 | 0.296 |
|  |  | 2 | 0.084 | 0.036 | 1.088 | **0.018** |
|  |  | 3 | 0.059 | 0.033 | 1.061 | 0.076 |
|  |  | 4 | 0.005 | 0.041 | 1.005 | 0.911 |
|  |  | 5 | 0.052 | 0.016 | 1.053 | **0.001** |
|  |  | 6 | 0.01 | 0.038 | 1.01 | 0.795 |
|  |  | 7 | 0.034 | 0.051 | 1.035 | 0.508 |
|  |  | 8 | 0.034 | 0.041 | 1.035 | 0.411 |
|  | Food insecurity | 1 | -0.061 | 0.06 | 0.941 | 0.313 |
|  |  | 2 | 0.021 | 0.057 | 1.022 | 0.708 |
|  |  | 3 | 0.007 | 0.052 | 1.007 | 0.901 |
|  |  | 4 | 0.137 | 0.062 | 1.147 | **0.027** |
|  |  | 5 | -0.146 | 0.026 | 0.864 | **<0.001** |
|  |  | 6 | -0.013 | 0.06 | 0.987 | 0.828 |
|  |  | 7 | 0.033 | 0.087 | 1.034 | 0.704 |
|  |  | 8 | 0.05 | 0.064 | 1.051 | 0.439 |
|  | Fair/Poor health | 1 | -0.091 | 0.071 | 0.913 | 0.202 |
|  |  | 2 | -0.207 | 0.072 | 0.813 | **0.004** |
|  |  | 3 | 0.044 | 0.055 | 1.045 | 0.429 |
|  |  | 4 | -0.016 | 0.075 | 0.984 | 0.829 |
|  |  | 5 | -0.086 | 0.029 | 0.918 | **0.003** |
|  |  | 6 | -0.236 | 0.075 | 0.79 | **0.002** |
|  |  | 7 | -0.016 | 0.099 | 0.984 | 0.873 |
|  |  | 8 | -0.006 | 0.076 | 0.994 | 0.940 |
| **Environmental** | Population density | 1 | 0.01 | 0.003 | 1.01 | **0.004** |
|  |  | 2 | 0.017 | 0.002 | 1.017 | **<0.001** |
|  |  | 3 | 0.015 | 0.002 | 1.015 | **<0.001** |
|  |  | 4 | 0.001 | 0.005 | 1.001 | 0.806 |
|  |  | 5 | 0.02 | 0.002 | 1.02 | **<0.001** |
|  |  | 6 | 0.015 | 0.002 | 1.015 | **<0.001** |
|  |  | 7 | -0.021 | 0.015 | 0.979 | 0.150 |
|  |  | 8 | 0.007 | 0.005 | 1.007 | 0.146 |
|  | AQI | 1 | 0.113 | 0.219 | 1.12 | 0.605 |
|  |  | 2 | -0.084 | 0.116 | 0.919 | 0.470 |
|  |  | 3 | 0.048 | 0.171 | 1.049 | 0.780 |
|  |  | 4 | 0.233 | 0.236 | 1.263 | 0.323 |
|  |  | 5 | 0.561 | 0.136 | 1.752 | **<0.001** |
|  |  | 6 | 0.033 | 0.144 | 1.034 | 0.818 |
|  |  | 7 | -0.221 | 0.053 | 0.802 | **<0.001** |
|  |  | 8 | 0.076 | 0.04 | 1.079 | 0.059 |
|  | Temperature | 1 | 0.033 | 0.026 | 1.034 | 0.198 |
|  |  | 2 | -0.025 | 0.024 | 0.975 | 0.303 |
|  |  | 3 | -0.012 | 0.023 | 0.988 | 0.594 |
|  |  | 4 | -0.069 | 0.028 | 0.933 | **0.013** |
|  |  | 5 | -0.006 | 0.012 | 0.994 | 0.607 |
|  |  | 6 | 0.02 | 0.024 | 1.021 | 0.389 |
|  |  | 7 | 0.072 | 0.042 | 1.075 | 0.086 |
|  |  | 8 | -0.045 | 0.032 | 0.956 | 0.160 |
|  | PM | 1 | -0.307 | 0.825 | 0.736 | 0.710 |
|  |  | 2 | 0.515 | 0.4 | 1.674 | 0.198 |
|  |  | 3 | 0.039 | 0.632 | 1.04 | 0.950 |
|  |  | 4 | -0.737 | 0.9 | 0.478 | 0.413 |
|  |  | 5 | -1.94 | 0.526 | 0.144 | **<0.001** |
|  |  | 6 | 0.057 | 0.524 | 1.059 | 0.913 |
|  |  | 7 | 0.491 | 0.028 | 1.635 | **<0.001** |
|  |  | 8 | -0.385 | 0.132 | 0.68 | **0.004** |

**Table S11.** **2^nd^ wave (Jun 4 to Sep 2, 2020, USA):** results from multinomial logit model for all clusters. The estimated odds ratios (ORs) show the association of each risk factor with increasing/decreasing the relative log odds of being in each cluster compare to cluster 0 (MIR=0)

|  | Variable | Cluster | Est. | SD | OR | P-value |
| --- | --- | --- | --- | --- | --- | --- |
| **Comorbidities & Disorders** | CVD | 1 | -0.064 | 0.090 | 0.938 | 0.476 |
|  |  | 2 | -0.074 | 0.060 | 0.928 | 0.214 |
|  |  | 3 | -0.121 | 0.095 | 0.886 | 0.205 |
|  |  | 4 | -0.003 | 0.077 | 0.997 | 0.964 |
|  |  | 5 | 0.016 | 0.088 | 1.017 | 0.852 |
|  | Cardiomyopathy & myocarditis | 1 | 0.024 | 0.140 | 1.024 | 0.866 |
|  |  | 2 | 0.017 | 0.088 | 1.017 | 0.849 |
|  |  | 3 | 0.109 | 0.134 | 1.115 | 0.418 |
|  |  | 4 | -0.138 | 0.123 | 0.871 | 0.263 |
|  |  | 5 | -0.058 | 0.151 | 0.944 | 0.702 |
|  | Hypertensive heart disease | 1 | 0.008 | 0.102 | 1.008 | 0.936 |
|  |  | 2 | 0.075 | 0.065 | 1.078 | 0.244 |
|  |  | 3 | 0.174 | 0.099 | 1.191 | 0.079 |
|  |  | 4 | -0.028 | 0.085 | 0.972 | 0.741 |
|  |  | 5 | -0.017 | 0.098 | 0.983 | 0.864 |
|  | Peripheral vascular disease | 1 | 0.403 | 0.430 | 1.496 | 0.349 |
|  |  | 2 | -0.237 | 0.312 | 0.789 | 0.447 |
|  |  | 3 | 0.518 | 0.422 | 1.678 | 0.220 |
|  |  | 4 | 0.510 | 0.383 | 1.665 | 0.183 |
|  |  | 5 | 0.087 | 0.533 | 1.090 | 0.871 |
|  | Atrial fibrillation | 1 | -0.042 | 0.211 | 0.959 | 0.842 |
|  |  | 2 | 0.010 | 0.116 | 1.010 | 0.929 |
|  |  | 3 | 0.076 | 0.191 | 1.079 | 0.690 |
|  |  | 4 | -0.092 | 0.167 | 0.912 | 0.581 |
|  |  | 5 | -0.364 | 0.235 | 0.695 | 0.121 |
|  | Cerebrovascular | 1 | 0.121 | 0.095 | 1.129 | 0.204 |
|  |  | 2 | 0.111 | 0.064 | 1.117 | 0.085 |
|  |  | 3 | 0.169 | 0.102 | 1.184 | 0.100 |
|  |  | 4 | 0.027 | 0.084 | 1.027 | 0.747 |
|  |  | 5 | 0.007 | 0.096 | 1.007 | 0.945 |
|  | Diabetes | 1 | 0.101 | 0.064 | 1.107 | 0.114 |
|  |  | **2** | **0.082** | **0.039** | **1.085** | **0.034** |
|  |  | 3 | 0.059 | 0.057 | 1.061 | 0.303 |
|  |  | 4 | 0.062 | 0.052 | 1.064 | 0.238 |
|  |  | 5 | 0.010 | 0.077 | 1.010 | 0.892 |
|  | Hepatitis | **1** | **2.572** | **0.512** | **13.093** | **<0.001** |
|  |  | **2** | **3.973** | **0.789** | **53.122** | **<0.001** |
|  |  | 3 | -0.394 | 0.367 | 0.674 | 0.282 |
|  |  | **4** | **-1.467** | **0.341** | **0.231** | **<0.001** |
|  |  | **5** | **2.630** | **0.403** | **13.876** | **<0.001** |
|  | HIV/AIDS | **1** | **0.831** | **0.336** | **2.295** | **0.013** |
|  |  | 2 | 0.584 | 0.327 | 1.794 | 0.074 |
|  |  | **3** | **1.056** | **0.332** | **2.875** | **0.001** |
|  |  | 4 | 0.652 | 0.358 | 1.920 | 0.068 |
|  |  | **5** | **0.778** | **0.344** | **2.177** | **0.024** |
|  | Tuberculosis | **1** | **0.762** | **0.288** | **2.143** | **0.008** |
|  |  | **2** | **3.795** | **1.131** | **44.472** | **0.001** |
|  |  | **3** | **-1.512** | **0.379** | **0.220** | **<0.001** |
|  |  | 4 | 0.393 | 0.318 | 1.482 | 0.216 |
|  |  | **5** | **0.566** | **0.255** | **1.760** | **0.026** |
|  | Asthma | 1 | -1.542 | 0.951 | 0.214 | 0.105 |
|  |  | **2** | **-1.179** | **0.575** | **0.308** | **0.040** |
|  |  | **3** | **-2.284** | **0.798** | **0.102** | **0.004** |
|  |  | 4 | 0.772 | 0.731 | 2.164 | 0.291 |
|  |  | 5 | -0.563 | 0.944 | 0.570 | 0.551 |
|  | Lower respiratory infection | 1 | -0.021 | 0.031 | 0.979 | 0.504 |
|  |  | 2 | -0.019 | 0.015 | 0.981 | 0.215 |
|  |  | 3 | 0.016 | 0.024 | 1.016 | 0.514 |
|  |  | 4 | 0.015 | 0.020 | 1.015 | 0.463 |
|  |  | 5 | -0.031 | 0.034 | 0.970 | 0.368 |
|  | Ischemia | 1 | 0.057 | 0.089 | 1.059 | 0.520 |
|  |  | 2 | 0.064 | 0.059 | 1.066 | 0.281 |
|  |  | 3 | 0.112 | 0.095 | 1.119 | 0.237 |
|  |  | 4 | -0.009 | 0.077 | 0.991 | 0.907 |
|  |  | 5 | -0.018 | 0.087 | 0.982 | 0.836 |
|  | Mesothelioma | 1 | -0.310 | 0.985 | 0.733 | 0.753 |
|  |  | 2 | 0.335 | 0.561 | 1.399 | 0.550 |
|  |  | 3 | -0.329 | 1.062 | 0.720 | 0.757 |
|  |  | 4 | -0.188 | 0.923 | 0.829 | 0.839 |
|  |  | 5 | -1.273 | 0.655 | 0.280 | 0.052 |
|  | Tracheal cancer | 1 | 0.003 | 0.027 | 1.003 | 0.920 |
|  |  | 2 | 0.024 | 0.014 | 1.025 | 0.092 |
|  |  | 3 | -0.018 | 0.023 | 0.982 | 0.436 |
|  |  | 4 | -0.002 | 0.021 | 0.998 | 0.934 |
|  |  | 5 | -0.031 | 0.030 | 0.969 | 0.300 |
|  | Pancreatic cancer | 1 | -0.285 | 0.268 | 0.752 | 0.288 |
|  |  | 2 | -0.143 | 0.145 | 0.867 | 0.325 |
|  |  | 3 | 0.064 | 0.217 | 1.066 | 0.770 |
|  |  | 4 | 0.193 | 0.200 | 1.213 | 0.334 |
|  |  | 5 | 0.176 | 0.285 | 1.192 | 0.537 |
|  | Rheumatic disease | 1 | 0.716 | 0.421 | 2.045 | 0.089 |
|  |  | 2 | 0.264 | 0.267 | 1.303 | 0.322 |
|  |  | **3** | **0.900** | **0.350** | **2.459** | **0.010** |
|  |  | 4 | -0.013 | 0.352 | 0.987 | 0.971 |
|  |  | 5 | 0.066 | 0.511 | 1.068 | 0.898 |
| **Demographics & Social** | Age | **1** | **-0.128** | **0.054** | **0.880** | **0.019** |
|  |  | **2** | **-0.202** | **0.034** | **0.817** | **<0.001** |
|  |  | 3 | -0.022 | 0.050 | 0.978 | 0.656 |
|  |  | **4** | **-0.124** | **0.047** | **0.883** | **0.008** |
|  |  | **5** | **-0.164** | **0.068** | **0.849** | **0.016** |
|  | Female-AA% | 1 | -0.660 | 0.381 | 0.517 | 0.083 |
|  |  | **2** | **4.616** | **0.655** | **101.137** | **<0.001** |
|  |  | **3** | **-0.896** | **0.359** | **0.408** | **0.012** |
|  |  | **4** | **-1.674** | **0.383** | **0.188** | **<0.001** |
|  |  | **5** | **-2.538** | **0.314** | **0.079** | **<0.001** |
|  | Female-WA% | 1 | -0.298 | 0.536 | 0.743 | 0.578 |
|  |  | **2** | **-2.848** | **0.541** | **0.058** | **<0.001** |
|  |  | 3 | 0.424 | 0.502 | 1.528 | 0.398 |
|  |  | 4 | -0.471 | 0.538 | 0.624 | 0.381 |
|  |  | 5 | 0.435 | 0.564 | 1.545 | 0.441 |
|  | Male-AA% | 1 | -0.388 | 0.355 | 0.678 | 0.274 |
|  |  | 2 | -0.670 | 0.717 | 0.512 | 0.350 |
|  |  | **3** | **1.074** | **0.342** | **2.927** | **0.002** |
|  |  | **4** | **-1.698** | **0.370** | **0.183** | **<0.001** |
|  |  | **5** | **-1.438** | **0.291** | **0.237** | **<0.001** |
|  | Male-WA% | 1 | -0.622 | 0.506 | 0.537 | 0.219 |
|  |  | **2** | **1.836** | **0.490** | **6.268** | **<0.001** |
|  |  | **3** | **-1.309** | **0.477** | **0.270** | **0.006** |
|  |  | 4 | -0.575 | 0.515 | 0.562 | 0.264 |
|  |  | 5 | -0.569 | 0.517 | 0.566 | 0.271 |
|  | Asian% | 1 | -0.142 | 0.347 | 0.868 | 0.683 |
|  |  | 2 | -0.030 | 0.180 | 0.970 | 0.866 |
|  |  | 3 | 0.109 | 0.247 | 1.115 | 0.660 |
|  |  | 4 | -0.010 | 0.254 | 0.990 | 0.967 |
|  |  | 5 | 0.027 | 0.335 | 1.027 | 0.936 |
|  | Smokers% | 1 | -0.253 | 0.155 | 0.777 | 0.102 |
|  |  | 2 | 0.066 | 0.080 | 1.068 | 0.406 |
|  |  | 3 | -0.145 | 0.126 | 0.865 | 0.248 |
|  |  | 4 | -0.141 | 0.115 | 0.868 | 0.220 |
|  |  | 5 | 0.286 | 0.156 | 1.331 | 0.066 |
|  | Unemployed% | **1** | **0.422** | **0.144** | **1.525** | **0.003** |
|  |  | **2** | **0.292** | **0.099** | **1.339** | **0.003** |
|  |  | 3 | 0.110 | 0.154 | 1.116 | 0.475 |
|  |  | **4** | **0.461** | **0.124** | **1.585** | **<0.001** |
|  |  | **5** | **0.427** | **0.175** | **1.532** | **0.015** |
|  | Income Rate | 1 | 0.375 | 0.341 | 1.456 | 0.271 |
|  |  | 2 | 0.196 | 0.196 | 1.216 | 0.319 |
|  |  | 3 | 0.295 | 0.274 | 1.343 | 0.281 |
|  |  | 4 | 0.085 | 0.273 | 1.089 | 0.755 |
|  |  | 5 | 0.216 | 0.394 | 1.241 | 0.584 |
|  | Uninsured% | 1 | 0.055 | 0.055 | 1.056 | 0.319 |
|  |  | 2 | 0.002 | 0.029 | 1.002 | 0.954 |
|  |  | 3 | 0.060 | 0.046 | 1.062 | 0.193 |
|  |  | 4 | 0.012 | 0.043 | 1.012 | 0.783 |
|  |  | 5 | 0.103 | 0.064 | 1.108 | 0.109 |
|  | Food insecurity | 1 | 0.037 | 0.086 | 1.038 | 0.667 |
|  |  | **2** | **-0.098** | **0.050** | **0.907** | **0.048** |
|  |  | 3 | 0.082 | 0.078 | 1.085 | 0.297 |
|  |  | 4 | -0.038 | 0.069 | 0.963 | 0.583 |
|  |  | 5 | 0.085 | 0.099 | 1.089 | 0.389 |
|  | Fair/Poor health | 1 | -0.017 | 0.102 | 0.983 | 0.869 |
|  |  | 2 | -0.062 | 0.058 | 0.940 | 0.287 |
|  |  | 3 | 0.118 | 0.087 | 1.125 | 0.178 |
|  |  | 4 | 0.038 | 0.081 | 1.038 | 0.641 |
|  |  | 5 | -0.240 | 0.130 | 0.787 | 0.064 |
| **Environmental** | Population density | 1 | 0.010 | 0.005 | 1.010 | 0.064 |
|  |  | **2** | **0.015** | **0.004** | **1.015** | **0.001** |
|  |  | **3** | **0.011** | **0.005** | **1.011** | **0.029** |
|  |  | **4** | **0.010** | **0.005** | **1.011** | **0.040** |
|  |  | **5** | **0.011** | **0.006** | **1.011** | **0.050** |
|  | AQI | 1 | 0.004 | 0.108 | 1.004 | 0.973 |
|  |  | 2 | -0.010 | 0.142 | 0.990 | 0.943 |
|  |  | **3** | **-0.816** | **0.198** | **0.442** | **<0.001** |
|  |  | 4 | 0.233 | 0.341 | 1.262 | 0.495 |
|  |  | 5 | 0.387 | 0.342 | 1.473 | 0.258 |
|  | Temperature | **1** | **0.159** | **0.044** | **1.172** | **<0.001** |
|  |  | **2** | **0.070** | **0.029** | **1.072** | **0.015** |
|  |  | **3** | **0.090** | **0.044** | **1.095** | **0.039** |
|  |  | **4** | **0.111** | **0.039** | **1.118** | **0.005** |
|  |  | 5 | -0.028 | 0.058 | 0.972 | 0.627 |
|  | PM | 1 | -0.137 | 0.370 | 0.872 | 0.711 |
|  |  | 2 | -0.103 | 0.477 | 0.902 | 0.829 |
|  |  | **3** | **2.461** | **0.615** | **11.715** | **<0.001** |
|  |  | 4 | -0.993 | 1.248 | 0.370 | 0.426 |
|  |  | 5 | -1.238 | 1.177 | 0.290 | 0.293 |

**Table S12.** **3^rd^ wave (Sep 3 - Nov 12, 2020, USA):** results from multinomial logit model for all clusters. The estimated odds ratios (ORs) show the association of each risk factor with increasing/decreasing the relative log odds of being in each cluster compare to cluster 0 (MIR=0)

|  | **Variable** | **Cluster** | **Est.** | **SD** | **OR** | **P-value** |
| --- | --- | --- | --- | --- | --- | --- |
| **Comorbidities & Disorders** | CVD | 1 | -0.074 | 0.051 | 0.928 | 0.148 |
|  |  | 2 | 0.010 | 0.064 | 1.010 | 0.872 |
|  |  | 3 | 0.168 | 0.091 | 1.183 | 0.067 |
|  |  | 4 | -0.041 | 0.038 | 0.960 | 0.289 |
|  | Cardiomyopathy & myocarditis | **1** | **0.241** | **0.114** | **1.272** | **0.034** |
|  |  | 2 | -0.075 | 0.174 | 0.928 | 0.667 |
|  |  | 3 | -0.097 | 0.225 | 0.908 | 0.668 |
|  |  | **4** | **0.194** | **0.099** | **1.214** | **0.049** |
|  | Hypertensive heart disease | 1 | 0.110 | 0.059 | 1.117 | 0.062 |
|  |  | 2 | -0.042 | 0.089 | 0.959 | 0.637 |
|  |  | 3 | -0.150 | 0.139 | 0.861 | 0.279 |
|  |  | 4 | 0.050 | 0.047 | 1.051 | 0.290 |
|  | Peripheral vascular disease | 1 | 0.261 | 0.376 | 1.299 | 0.487 |
|  |  | 2 | 0.137 | 0.483 | 1.147 | 0.776 |
|  |  | 3 | 0.307 | 0.517 | 1.360 | 0.552 |
|  |  | 4 | -0.025 | 0.323 | 0.975 | 0.939 |
|  | Cerebrovascular | 1 | 0.091 | 0.058 | 1.095 | 0.116 |
|  |  | 2 | 0.038 | 0.071 | 1.039 | 0.594 |
|  |  | 3 | -0.107 | 0.097 | 0.898 | 0.268 |
|  |  | 4 | 0.066 | 0.044 | 1.068 | 0.132 |
|  | Diabetes | **1** | **0.144** | **0.060** | **1.155** | **0.016** |
|  |  | 2 | 0.038 | 0.083 | 1.038 | 0.649 |
|  |  | 3 | -0.234 | 0.155 | 0.791 | 0.130 |
|  |  | 4 | 0.093 | 0.048 | 1.098 | 0.053 |
|  | Hepatitis | **1** | **-0.604** | **0.030** | **0.547** | **<0.001** |
|  |  | **2** | **-1.967** | **0.018** | **0.140** | **<0.001** |
|  |  | **3** | **-0.565** | **0.023** | **0.568** | **<0.001** |
|  |  | **4** | **9.220** | **0.078** | **10099.235^*^** | **<0.001** |
|  | HIV/AIDS | 1 | 0.103 | 0.429 | 1.109 | 0.809 |
|  |  | **2** | **1.376** | **0.403** | **3.961** | **0.001** |
|  |  | **3** | **4.367** | **0.217** | **78.823** | **<0.001** |
|  |  | 4 | -0.581 | 0.382 | 0.559 | 0.128 |
|  | Tuberculosis | **1** | **1.171** | **0.067** | **3.224** | **<0.001** |
|  |  | **2** | **3.513** | **0.319** | **33.554** | **<0.001** |
|  |  | **3** | **1.952** | **0.031** | **7.045** | **<0.001** |
|  |  | **4** | **3.446** | **0.314** | **31.373** | **<0.001** |
|  | Lower respiratory infection | 1 | 0.038 | 0.028 | 1.038 | 0.172 |
|  |  | **2** | **0.080** | **0.033** | **1.084** | **0.016** |
|  |  | **3** | **0.120** | **0.055** | **1.128** | **0.028** |
|  |  | 4 | 0.036 | 0.022 | 1.036 | 0.104 |
|  | Interstitial lung disease & pulmonary sarcoidosis | 1 | 0.231 | 0.208 | 1.260 | 0.268 |
|  |  | **2** | **-0.644** | **0.311** | **0.525** | **0.038** |
|  |  | 3 | 0.105 | 0.448 | 1.111 | 0.814 |
|  |  | 4 | -0.101 | 0.170 | 0.904 | 0.551 |
|  | Asthma | 1 | -0.332 | 0.695 | 0.718 | 0.633 |
|  |  | 2 | 0.193 | 0.926 | 1.212 | 0.835 |
|  |  | **3** | **-0.866** | **0.281** | **0.421** | **0.002** |
|  |  | 4 | -0.139 | 0.539 | 0.870 | 0.796 |
|  | COPD | 1 | 0.009 | 0.020 | 1.009 | 0.660 |
|  |  | 2 | 0.018 | 0.027 | 1.018 | 0.502 |
|  |  | 3 | -0.017 | 0.051 | 0.983 | 0.733 |
|  |  | 4 | -0.013 | 0.016 | 0.987 | 0.415 |
|  | Ischemia | 1 | 0.096 | 0.051 | 1.100 | 0.059 |
|  |  | 2 | 0.019 | 0.064 | 1.019 | 0.769 |
|  |  | 3 | -0.168 | 0.093 | 0.846 | 0.071 |
|  |  | 4 | 0.046 | 0.038 | 1.047 | 0.231 |
|  | Mesothelioma | **1** | **2.234** | **0.410** | **9.334** | **<0.001** |
|  |  | **2** | **3.021** | **0.427** | **20.506** | **<0.001** |
|  |  | **3** | **1.291** | **0.073** | **3.637** | **<0.001** |
|  |  | **4** | **2.168** | **0.299** | **8.743** | **<0.001** |
|  | Tracheal cancer | **1** | **-0.071** | **0.027** | **0.932** | **0.008** |
|  |  | 2 | -0.022 | 0.035 | 0.978 | 0.532 |
|  |  | 3 | -0.068 | 0.066 | 0.934 | 0.303 |
|  |  | 4 | -0.022 | 0.022 | 0.978 | 0.306 |
|  | Leukemia | 1 | -0.065 | 0.331 | 0.937 | 0.844 |
|  |  | 2 | -0.008 | 0.424 | 0.992 | 0.985 |
|  |  | 3 | 0.211 | 0.819 | 1.235 | 0.796 |
|  |  | 4 | 0.241 | 0.270 | 1.273 | 0.372 |
|  | Pancreatic cancer | 1 | 0.509 | 0.289 | 1.664 | 0.078 |
|  |  | 2 | -0.188 | 0.387 | 0.828 | 0.626 |
|  |  | 3 | -0.052 | 0.678 | 0.949 | 0.939 |
|  |  | 4 | 0.101 | 0.242 | 1.107 | 0.675 |
|  | Drug use disorder | 1 | -0.061 | 0.066 | 0.941 | 0.354 |
|  |  | **2** | **-0.293** | **0.108** | **0.746** | **0.007** |
|  |  | 3 | 0.141 | 0.133 | 1.152 | 0.287 |
|  |  | 4 | -0.066 | 0.058 | 0.936 | 0.257 |
|  | Alcohol use disorder | **1** | **-0.436** | **0.174** | **0.647** | **0.012** |
|  |  | 2 | -0.081 | 0.140 | 0.922 | 0.561 |
|  |  | 3 | -0.753 | 0.438 | 0.471 | 0.086 |
|  |  | 4 | -0.200 | 0.118 | 0.818 | 0.088 |
| **Demographics & Social** | Female-AA% | **1** | **3.509** | **0.105** | **33.431** | **<0.001** |
|  |  | **2** | **-2.986** | **0.031** | **0.050** | **<0.001** |
|  |  | **3** | **-0.867** | **0.022** | **0.420** | **<0.001** |
|  |  | **4** | **3.132** | **0.123** | **22.921** | **<0.001** |
|  | Female-WA% | **1** | **-3.626** | **0.402** | **0.027** | **<0.001** |
|  |  | **2** | **-3.931** | **0.147** | **0.020** | **<0.001** |
|  |  | **3** | **-2.150** | **0.059** | **0.117** | **<0.001** |
|  |  | **4** | **-7.248** | **0.777** | **0.001** | **<0.001** |
|  | Male-AA% | **1** | **-2.008** | **0.100** | **0.134** | **<0.001** |
|  |  | **2** | **-2.479** | **0.038** | **0.084** | **<0.001** |
|  |  | **3** | **-2.453** | **0.029** | **0.086** | **<0.001** |
|  |  | **4** | **0.257** | **0.123** | **1.293** | **0.037** |
|  | Smokers% | **1** | **0.257** | **0.125** | **1.293** | **0.040** |
|  |  | 2 | -0.043 | 0.162 | 0.958 | 0.792 |
|  |  | 3 | -0.035 | 0.310 | 0.965 | 0.909 |
|  |  | 4 | 0.143 | 0.102 | 1.154 | 0.160 |
|  | Unemployed% | 1 | 0.109 | 0.154 | 1.115 | 0.479 |
|  |  | 2 | 0.209 | 0.193 | 1.233 | 0.278 |
|  |  | 3 | -0.122 | 0.362 | 0.885 | 0.736 |
|  |  | 4 | -0.162 | 0.125 | 0.850 | 0.196 |
|  | Income Rate | 1 | 0.326 | 0.308 | 1.386 | 0.289 |
|  |  | 2 | 0.229 | 0.336 | 1.257 | 0.497 |
|  |  | 3 | -1.310 | 0.806 | 0.270 | 0.104 |
|  |  | 4 | -0.210 | 0.236 | 0.811 | 0.374 |
|  | Uninsured% | 1 | 0.011 | 0.066 | 1.011 | 0.863 |
|  |  | 2 | -0.098 | 0.085 | 0.907 | 0.252 |
|  |  | 3 | 0.062 | 0.153 | 1.064 | 0.683 |
|  |  | 4 | -0.074 | 0.051 | 0.929 | 0.147 |
|  | Food insecurity | **1** | **-0.217** | **0.096** | **0.805** | **0.024** |
|  |  | 2 | -0.174 | 0.107 | 0.840 | 0.103 |
|  |  | 3 | 0.063 | 0.214 | 1.065 | 0.769 |
|  |  | 4 | -0.116 | 0.073 | 0.890 | 0.110 |
|  | Fair/Poor health | 1 | -0.200 | 0.129 | 0.819 | 0.121 |
|  |  | 2 | 0.108 | 0.166 | 1.114 | 0.514 |
|  |  | 3 | 0.199 | 0.299 | 1.221 | 0.504 |
|  |  | 4 | 0.010 | 0.101 | 1.010 | 0.923 |
| **Environmental** | Population density | **1** | **0.016** | **0.006** | **1.017** | **0.011** |
|  |  | 2 | 0.015 | 0.008 | 1.015 | 0.055 |
|  |  | 3 | 0.013 | 0.008 | 1.013 | 0.120 |
|  |  | **4** | **0.016** | **0.006** | **1.016** | **0.014** |
|  | AQI | 1 | 0.466 | 0.256 | 1.593 | 0.069 |
|  |  | **2** | **-0.329** | **0.160** | **0.720** | **0.040** |
|  |  | 3 | 0.119 | 0.073 | 1.127 | 0.104 |
|  |  | **4** | **0.320** | **0.112** | **1.378** | **0.004** |
|  | Temperature | 1 | 0.026 | 0.045 | 1.026 | 0.562 |
|  |  | 2 | -0.015 | 0.054 | 0.985 | 0.783 |
|  |  | **3** | **-0.252** | **0.108** | **0.777** | **0.020** |
|  |  | 4 | -0.056 | 0.036 | 0.946 | 0.117 |
|  | PM | 1 | -1.764 | 0.983 | 0.171 | 0.073 |
|  |  | 2 | 0.943 | 0.523 | 2.569 | 0.071 |
|  |  | **3** | **-0.558** | **0.053** | **0.572** | **<0.001** |
|  |  | **4** | **-1.052** | **0.386** | **0.349** | **0.006** |
| ^** Due to the sparsity of hepatitis mortality rate in these particular counties (during the 3rd wave), the odds ratio estimation of hepatitis is not reliable. One way around this issue is to categorize the hepatitis MR and use the categorical version of this variable in the multinomial model. However, we decided to avoide this approach to stay consistent with the rest of the results.^ | | | | | | |
|  |  |  |  |  |  |  |

**REFERENCES**

1 Huang, C. *et al.* Clinical features of patients infected with 2019 novel coronavirus in Wuhan, China. *The lancet* **395**, 497-506 (2020).

2 Halpin, D. M., Faner, R., Sibila, O., Badia, J. R. & Agusti, A. Do chronic respiratory diseases or their treatment affect the risk of SARS-CoV-2 infection? *Lanc. Resp. Med.* **8**, 436-438 (2020).

3 Onder, G., Rezza, G. & Brusaferro, S. Case-fatality rate and characteristics of patients dying in relation to COVID-19 in Italy. *JAMA* **323**, 1775-1776 (2020).

4 Covid, C. *et al.* Preliminary estimates of the prevalence of selected underlying health conditions among patients with coronavirus disease 2019—United States, February 12–March 28, 2020. *Morb. Mort. Wkly.* **69**, 382 (2020).

5 Organization, W. H. Coronavirus disease 2019 (COVID-19): situation report, 73. (2020).

6 Zhao, Q. *et al.* The impact of COPD and smoking history on the severity of Covid‐19: A systemic review and meta‐analysis. *J. Med. Virol.* (2020).

7 Guan, W.-j. *et al.* Clinical characteristics of coronavirus disease 2019 in China. *N. Engl. J. Med.* **382**, 1708-1720 (2020).

8 Alqahtani, J. S. *et al.* Prevalence, severity and mortality associated with COPD and smoking in patients with COVID-19: a rapid systematic review and meta-analysis. *PLoS One* **15**, e0233147 (2020).

9 Brake, S. J. *et al.* Smoking upregulates angiotensin-converting enzyme-2 receptor: a potential adhesion site for novel coronavirus SARS-CoV-2 (Covid-19). *MDP* (2020).

10 Zhao, Y. *et al.* Single-cell RNA expression profiling of ACE2, the putative receptor of Wuhan 2019-nCov. *BioRxiv* (2020).

11 Bhat, T. A., Panzica, L., Kalathil, S. G. & Thanavala, Y. Immune dysfunction in patients with chronic obstructive pulmonary disease. *Ann. Am. Thorac. Soc.* **12**, S169-S175 (2015).

12 Hendren, N. S., Drazner, M. H., Bozkurt, B. & Cooper, J., Leslie T. Description and proposed management of the acute COVID-19 cardiovascular syndrome. *Circulation* (2020).

13 Shi, S. *et al.* Association of cardiac injury with mortality in hospitalized patients with COVID-19 in Wuhan, China. *JAMA cardiol.* (2020).

14 Lippi, G., Lavie, C. J. & Sanchis-Gomar, F. Cardiac troponin I in patients with coronavirus disease 2019 (COVID-19): Evidence from a meta-analysis. *Prog. Cardiovasc. Dis.* (2020).

15 Hamming, I. *et al.* Tissue distribution of ACE2 protein, the functional receptor for SARS coronavirus. A first step in understanding SARS pathogenesis. *J. of Pathol.* **203**, 631-637 (2004).

16 Crackower, M. A. *et al.* Angiotensin-converting enzyme 2 is an essential regulator of heart function. *Nature* **417**, 822-828 (2002).

17 Chen, L., Li, X., Chen, M., Feng, Y. & Xiong, C. The ACE2 expression in human heart indicates new potential mechanism of heart injury among patients infected with SARS-CoV-2. *Cardiovasc. Res.* **116**, 1097-1100 (2020).

18 Tang, N., Li, D., Wang, X. & Sun, Z. Abnormal coagulation parameters are associated with poor prognosis in patients with novel coronavirus pneumonia. *J. Thromb. Haemost.* **18**, 844-847 (2020).

19 Danzi, G., Loffi, M., Galeazzi, G. & Gherbesi, E. Acute pulmonary embolism and COVID-19 pneumonia: a random association? *Eur. Heart J.* (2020).

20 Ammirati, E. *et al.* Clinical Presentation and Outcome in a Contemporary Cohort of Patients With Acute Myocarditis: Multicenter Lombardy Registry. *Circulation* **138**, 1088-1099 (2018).

21 Hufnagel, G. *et al.* The European Study of Epidemiology and Treatment of Cardiac Inflammatory Diseases (ESETCID) First Epidemiological Results. *Herz* **25**, 279-285 (2000).

22 Li, W. *et al.* Angiotensin-converting enzyme 2 is a functional receptor for the SARS coronavirus. *Nature* **426**, 450-454 (2003).

23 Zhou, G., Zhao, J. & Wang, S. Pathological study of impact of SARS coronavirus on heart and its conduction system in SARS patients. *MJCPLA* (1982).

24 Oudit, G. *et al.* SARS‐coronavirus modulation of myocardial ACE2 expression and inflammation in patients with SARS. *Eur. J. Clin. Invest.* **39**, 618-625 (2009).

25 Booth, C. M. *et al.* Clinical features and short-term outcomes of 144 patients with SARS in the greater Toronto area. *JAMA* **289**, 2801-2809 (2003).

26 Li, B. *et al.* Prevalence and impact of cardiovascular metabolic diseases on COVID-19 in China. *Clin. Res. Cardiol.*, 1-8 (2020).

27 South, A. M., Diz, D. I. & Chappell, M. C. COVID-19, ACE2, and the cardiovascular consequences. *Am. J. Physiol. Heart and Circ.* **318**, H1084-H1090 (2020).

28 Gupta, R., Ghosh, A., Singh, A. K. & Misra, A. Clinical considerations for patients with diabetes in times of COVID-19 epidemic. *Diabetes Metab. Syndr.* **14**, 211 (2020).

29 Yang, J. *et al.* Plasma glucose levels and diabetes are independent predictors for mortality and morbidity in patients with SARS. *Diabet. Med.* **23**, 623-628 (2006).

30 Schoen, K., Horvat, N., Guerreiro, N. F., de Castro, I. & de Giassi, K. S. Spectrum of clinical and radiographic findings in patients with diagnosis of H1N1 and correlation with clinical severity. *BMC Infect. Dis.* **19**, 1-8 (2019).

31 Song, Z. *et al.* From SARS to MERS, thrusting coronaviruses into the spotlight. *Viruses* **11**, 59 (2019).

32 Jain, V. & Yuan, J.-M. Systematic review and meta-analysis of predictive symptoms and comorbidities for severe COVID-19 infection. *medRxiv* (2020).

33 Wu, Z. & McGoogan, J. M. Characteristics of and important lessons from the coronavirus disease 2019 (COVID-19) outbreak in China: summary of a report of 72 314 cases from the Chinese Center for Disease Control and Prevention. *JAMA* **323**, 1239-1242 (2020).

34 Yang, X. *et al.* Clinical course and outcomes of critically ill patients with SARS-CoV-2 pneumonia in Wuhan, China: a single-centered, retrospective, observational study. *Lancet Resp. Med.* (2020).

35 Pal, R. & Bhansali, A. COVID-19, diabetes mellitus and ACE2: the conundrum. *Diabetes Res. Clin. Pract.* **162** (2020).

36 Geerlings, S. E. & Hoepelman, A. I. Immune dysfunction in patients with diabetes mellitus (DM). *FEMS Immunol. Med. Microbiol.* **26**, 259-265 (1999).

37 Jafar, N., Edriss, H. & Nugent, K. The effect of short-term hyperglycemia on the innate immune system. *Am. J. Med. Sci.* **351**, 201-211 (2016).

38 Guo, W. *et al.* Diabetes is a risk factor for the progression and prognosis of COVID‐19. *Diabetes Metab. Res. Rev.*, e3319 (2020).

39 Maddaloni, E. & Buzzetti, R. Covid‐19 and diabetes mellitus: unveiling the interaction of two pandemics. *Diabetes Metab. Res. Rev.*, e33213321 (2020).

40 Tikellis, C. & Thomas, M. Angiotensin-converting enzyme 2 (ACE2) is a key modulator of the renin angiotensin system in health and disease. *Int. J. Pept.* **2012** (2012).

41 Zou, Z. *et al.* Angiotensin-converting enzyme 2 protects from lethal avian influenza A H5N1 infections. *Nat. Commun.* **5**, 1-7 (2014).

42 Wu, C. *et al.* Risk factors associated with acute respiratory distress syndrome and death in patients with coronavirus disease 2019 pneumonia in Wuhan, China. *JAMA int. med.* (2020).

43 Iacobellis, G. COVID-19 and diabetes: can DPP4 inhibition play a role? *Diabetes Res. Clin. Pract.* **162** (2020).

44 Kulcsar, K. A., Coleman, C. M., Beck, S. E. & Frieman, M. B. Comorbid diabetes results in immune dysregulation and enhanced disease severity following MERS-CoV infection. *JCI insight* **4** (2019).

45 Li, K. *et al.* Mouse-adapted MERS coronavirus causes lethal lung disease in human DPP4 knockin mice. *PNAS* **114**, E3119-E3128 (2017).

46 Fan, C. *et al.* A human DPP4-knockin mouse’s susceptibility to infection by authentic and pseudotyped MERS-CoV. *Viruses* **10**, 448 (2018).

47 Conarello, S. L. *et al.* Mice lacking dipeptidyl peptidase IV are protected against obesity and insulin resistance. *PNAS* **100**, 6825-6830 (2003).

48 Lippi, G. & Plebani, M. Laboratory abnormalities in patients with COVID-2019 infection. *Clin. Chemist. Lab. Med.* **1** (2020).

49 Fan, Z. *et al.* Clinical features of COVID-19-related liver damage. *Clin. Gastroenterol. Hepatol.* (2020).

50 Chen, N. *et al.* Epidemiological and clinical characteristics of 99 cases of 2019 novel coronavirus pneumonia in Wuhan, China: a descriptive study. *The Lancet* **395**, 507-513 (2020).

51 Hashimoto, T. *et al.* ACE2 links amino acid malnutrition to microbial ecology and intestinal inflammation. *Nature* **487**, 477-481 (2012).

52 Chai, X. *et al.* Specific ACE2 expression in cholangiocytes may cause liver damage after 2019-nCoV infection. *BioRxiv* (2020).

53 Ong, J., Young, B. E. & Ong, S. COVID-19 in gastroenterology: a clinical perspective. *Gut* **69**, 1144-1145 (2020).

54 Muniyappa, R. & Gubbi, S. COVID-19 pandemic, coronaviruses, and diabetes mellitus. *Am. J. of Physiol. Endocrinol. Metab.* **318**, E736-E741 (2020).

55 Richardson, S. *et al.* Presenting characteristics, comorbidities, and outcomes among 5700 patients hospitalized with COVID-19 in the New York City area. *JAMA* (2020).

56 Ferguson, N. *et al.* Report 9: Impact of non-pharmaceutical interventions (NPIs) to reduce COVID19 mortality and healthcare demand. (2020).

57 Wang, D. *et al.* Clinical characteristics of 138 hospitalized patients with 2019 novel coronavirus–infected pneumonia in Wuhan, China. *JAMA* **323**, 1061-1069 (2020).

58 Stang, A., Standl, F. & Jöckel, K.-H. Characteristics of COVID-19 pandemic and public health consequences. *Herz*, 1 (2020).

59 Standl, F., Jöckel, K.-H. & Stang, A. COVID-19 and the need of targeted inverse quarantine. *Eur. J. Epidemiol.*, 1-2 (2020).

60 Dong, Y. *et al.* Epidemiology of COVID-19 among children in China. *Pediatrics* **145** (2020).

61 Emami, A., Javanmardi, F., Pirbonyeh, N. & Akbari, A. Prevalence of underlying diseases in hospitalized patients with COVID-19: a systematic review and meta-analysis. *Arc. Acad. Emerg. Med.* **8** (2020).

62 Wenham, C., Smith, J. & Morgan, R. COVID-19: the gendered impacts of the outbreak. *The Lancet* **395**, 846-848 (2020).

63 Feng, Z. The Novel Coronavirus Pneumonia Emergency Response Epidemiology Team. *China CDC Weekly* **2**, 113-122 (2020).

64 Cai, H. Sex difference and smoking predisposition in patients with COVID-19. *The Lancet Resp. Med.* **8**, e20 (2020).

65 Atto, B. *et al.* New therapeutic targets for the prevention of infectious acute exacerbations of COPD: role of epithelial adhesion molecules and inflammatory pathways. *Clin. Sci.* **133**, 1663-1703 (2019).

66 Lawrence, H., Hunter, A., Murray, R., Lim, W. & McKeever, T. Cigarette smoking and the occurrence of influenza–Systematic review. *J. Infect.* **79**, 401-406 (2019).

67 Eapen, M. S., Sharma, P. & Sohal, S. S. Mitochondrial dysfunction in macrophages: a key to defective bacterial phagocytosis in COPD. *Eur. Respiratory Soc.* (2019).

68 Sohal, S. S. Inhaled corticosteroids and increased microbial load in COPD: potential role of epithelial adhesion molecules. *Eur. Respir. J.* **51**, 1702257 (2018).

69 Zhi, K. *et al.* Trends in cigarette smoking among older male adults in China: An urban–rural comparison. *J. Appl. Gerontol.* **38**, 884-901 (2019).

70 Li, F., Li, W., Farzan, M. & Harrison, S. C. Structure of SARS coronavirus spike receptor-binding domain complexed with receptor. *Science* **309**, 1864-1868 (2005).

71 Wang, J., Luo, Q., Chen, R., Chen, T. & Li, J. Susceptibility analysis of COVID-19 in smokers based on ACE2. (2020).

72 Lippi, G. & Henry, B. M. Active smoking is not associated with severity of coronavirus disease 2019 (COVID-19). *Eur. J. Intern. Med.* (2020).

73 Wang, J., Tang, K., Feng, K. & Lv, W. High temperature and high humidity reduce the transmission of COVID-19. *Available at SSRN 3551767* (2020).

74 Wang, M. *et al.* Temperature significant change COVID-19 Transmission in 429 cities. *MedRxiv (2020).*

75 Zhu, Y. & Xie, J. Association between ambient temperature and COVID-19 infection in 122 cities from China. *Sci. Total Environ.*, 138201 (2020).

76 Wang, M. *et al.* Temperature significant change COVID-19 Transmission in 429 cities. *MedRxiv* (2020).

77 Bannister-Tyrrell, M., Meyer, A., Faverjon, C. & Cameron, A. Preliminary evidence that higher temperatures are associated with lower incidence of COVID-19, for cases reported globally up to 29th February 2020. *MedRxiv* (2020).

78 Qi, H. *et al.* COVID-19 transmission in Mainland China is associated with temperature and humidity: A time-series analysis. *Sci. Total Environ.*, 138778 (2020).

79 Jüni, P. *et al.* Impact of climate and public health interventions on the COVID-19 pandemic: a prospective cohort study. *CMAJ* **192**, E566-E573 (2020).

80 Sobur, M. A. *et al.* Temperature and relative humidity are not major contributing factor on the occurrence of COVID-19 pandemic: An observational study in 57 countries. *MedRxiv* (2020).

81 Wu, X., Nethery, R. C., Sabath, B. M., Braun, D. & Dominici, F. Exposure to air pollution and COVID-19 mortality in the United States. *MedRxiv* (2020).

82 Morales, K. F., Paget, J. & Spreeuwenberg, P. Possible explanations for why some countries were harder hit by the pandemic influenza virus in 2009–a global mortality impact modeling study. *BMC Infect. Dis.* **17**, 642 (2017).

83 Clay, K., Lewis, J. & Severnini, E. What explains cross-city variation in mortality during the 1918 influenza pandemic? Evidence from 438 US cities. *Econ. Hum. Biol.* **35**, 42-50 (2019).

84 Clay, K., Lewis, J. & Severnini, E. Pollution, infectious disease, and mortality: evidence from the 1918 Spanish influenza pandemic. *Hist.* **78**, 1179-1209 (2018).

85 Kaan, P. M. & Hegele, R. G. Interaction between respiratory syncytial virus and particulate matter in guinea pig alveolar macrophages. *Am. J. Respir. Cell Mol. Biol.* **28**, 697-704 (2003).

86 Lambert, A. L., Trasti, F. S., Mangum, J. B. & Everitt, J. I. Effect of preexposure to ultrafine carbon black on respiratory syncytial virus infection in mice. *Toxicol. Sci.* **72**, 331-338 (2003).

87 Ciencewicki, J. & Jaspers, I. Air pollution and respiratory viral infection. *Inhal. Toxicol.* **19**, 1135-1146 (2007).

88 Ye, Q., Fu, J.-f., Mao, J.-h. & Shang, S.-q. Haze is a risk factor contributing to the rapid spread of respiratory syncytial virus in children. *Env. Sci. Pollut. Res.* **23**, 20178-20185 (2016).

89 Mollalo, A., Vahedi, B. & Rivera, K. M. GIS-based spatial modeling of COVID-19 incidence rate in the continental United States. *Sci. Total Environ.*, 138884 (2020).

90 Mollalo, A., Rivera, K. M. & Vahedi, B. Artificial Neural Network Modeling of Novel Coronavirus (COVID-19) Incidence Rates across the Continental United States. *Int. J. Environ. Res. Public Health* **17**, 4204 (2020).

91 Setti, L. *et al.* The potential role of particulate matter in the spreading of COVID-19 in Northern Italy: first evidence-based research hypotheses. *MedRxiv* (2020).

92 Bontempi, E. First data analysis about possible COVID-19 virus airborne diffusion due to air particulate matter (PM): the case of Lombardy (Italy). *Environ. Res.*, 109639 (2020).

93 Fattorini, D. & Regoli, F. Role of the chronic air pollution levels in the Covid-19 outbreak risk in Italy. *Env. Pollut.*, 114732 (2020).

94 Conticini, E., Frediani, B. & Caro, D. Can atmospheric pollution be considered a co-factor in extremely high level of SARS-CoV-2 lethality in Northern Italy? *Env. pollut.*, 114465 (2020).
